# Estrogen-mediated gut microbiome alterations influence sexual dimorphism in metabolic syndrome in mice

**DOI:** 10.1186/s40168-018-0587-0

**Published:** 2018-11-13

**Authors:** Kanakaraju Kaliannan, Ruairi C. Robertson, Kiera Murphy, Catherine Stanton, Chao Kang, Bin Wang, Lei Hao, Atul K. Bhan, Jing X. Kang

**Affiliations:** 1 Laboratory of Lipid Medicine and Technology, Department of Medicine, Massachusetts General Hospital and Harvard Medical School, 149 -13th Street, Boston, MA 02129 USA; 2 School of Microbiology, University College Cork, Cork, Ireland; 3 Teagasc Moorepark Food Research Centre, Fermoy, Co., Cork, Ireland; 4 APC Microbiome Institute, University College Cork, Cork, Ireland; 5 Research Center for Nutrition and Food Safety, Institute of Military Preventive Medicine, Third Military Medical University, Chongqing Key Laboratory of Nutrition and Food Safety, Chongqing Medical Nutrition Research Center, Chongqing, People’s Republic of China; 6 Department of Pathology, Massachusetts General Hospital and Harvard Medical School, Boston, MA 02114 USA

**Keywords:** Estrogen, Gut microbiome, Obesity, Metabolic syndrome, Isoflavones, Chronic inflammation

## Abstract

**Background:**

Understanding the mechanism of the sexual dimorphism in susceptibility to obesity and metabolic syndrome (MS) is important for the development of effective interventions for MS.

**Results:**

Here we show that gut microbiome mediates the preventive effect of estrogen (17β-estradiol) on metabolic endotoxemia (ME) and low-grade chronic inflammation (LGCI), the underlying causes of MS and chronic diseases. The characteristic profiles of gut microbiome observed in female and 17β-estradiol-treated male and ovariectomized mice, such as decreased *Proteobacteria* and lipopolysaccharide biosynthesis, were associated with a lower susceptibility to ME, LGCI, and MS in these animals. Interestingly, fecal microbiota-transplant from male mice transferred the MS phenotype to female mice, while antibiotic treatment eliminated the sexual dimorphism in MS, suggesting a causative role of the gut microbiome in this condition. Moreover, estrogenic compounds such as isoflavones exerted microbiome-modulating effects similar to those of 17β-estradiol and reversed symptoms of MS in the male mice. Finally, both expression and activity of intestinal alkaline phosphatase (IAP), a gut microbiota-modifying non-classical anti-microbial peptide, were upregulated by 17β-estradiol and isoflavones, whereas inhibition of IAP induced ME and LGCI in female mice, indicating a critical role of IAP in mediating the effects of estrogen on these parameters.

**Conclusions:**

In summary, we have identified a previously uncharacterized microbiome-based mechanism that sheds light upon sexual dimorphism in the incidence of MS and that suggests novel therapeutic targets and strategies for the management of obesity and MS in males and postmenopausal women.

**Electronic supplementary material:**

The online version of this article (10.1186/s40168-018-0587-0) contains supplementary material, which is available to authorized users.

## Background

Metabolic syndrome (MS) is a cluster of metabolic abnormalities including obesity, visceral adiposity, hyperinsulinemia, hyperglycemia, hypertension, and hypercholesterolemia [1]. It is a leading health issue facing western societies owing to the high sucrose, high saturated fat content, and elevated omega-6/omega-3 fatty acid ratio of the western diet (WD) [2]. Sexual dimorphism in obesity and metabolic dysfunction are observed in both experimental animal models of MS [3–5] and in humans [6]. In fact, in many rodent models, insulin resistance occurs rarely in females or exclusively in males [3]. Moreover, protection from severe high-fat diet (HFD)-induced obesity and MS in C57BL/6 female mice precludes the interrogation of disease pathogenesis in a sex-independent manner [7]. Sex steroid hormones are believed to underlie sexual dimorphism in metabolic outcomes in response to stressors such as the WD, with estrogens theorized to protect women until menopause [8]. Supporting this position, the prevalence of MS is higher in men than in similarly aged pre-menopausal women [6] and a higher level of adiposity is required in women to elicit metabolic disturbances [9]. Conversely, following the menopause, women tend to accumulate visceral fat and become more insulin resistant, with a consequent increase in the risk of type 2 diabetes [10]. An increasing body of evidence suggests that estrogens also have important beneficial effects on body fat and metabolism in males [10, 11].

The gut microbiota comprises trillions of bacteria that contribute to nutrient acquisition and energy regulation [12, 13]. Growing evidence indicates that obesity is closely linked with low-grade chronic inflammation (LGCI), which can lead to MS [14, 15]. In addition, changes in the composition of the gut microbiota are known to be associated with the development of obesity and its associated metabolic disorders [16]. Interestingly, an increased ratio of the major phyla *Firmicutes* and *Bacteroidetes* (FIR/BAC ratio) and depletion of several bacterial species (e.g., *Akkermansia mucinophilia*) can promote the development of obesity in both dietary and genetic models of obesity in mice [17–19]. Other studies in animal models of obesity suggest that obesity-induced gut dysbiosis caused by either environmental or genetic factors increase populations of bacteria that produce the endotoxin lipopolysaccharide (LPS) [14] and decrease LPS-suppressing bacteria [20, 21]. This process leads to impaired gut barrier integrity and release of LPS from intestinal gram-negative bacteria into the bloodstream [14, 22] which in turn leads to Toll-like receptor 4 (TLR4)-mediated metabolic endotoxemia (ME), LGCI and insulin resistance in obese mice [23, 24]. Moreover, chronic injection of LPS in mice causes mild obesity and insulin resistance [23], highlighting a possible role for microbiota-derived LPS in obesity-induced inflammation.

The causative role of the gut microbiota in the context of MS is well characterized [14, 20, 21], but the role of sexual dimorphism on the composition of gut microbiota in the context of MS, and the associated mechanisms underlying such differences, are still unclear. Here, we report that sexual dimorphism in MS is associated with estrogen-mediated changes in the gut microbiome, ME and LGCI, and that 17β-estradiol (17β-E) (E2) treatment prevents MS in male and ovariectomized (OVX) mice by altering gut microbiome and intestinal alkaline phosphatase (IAP), a major gut microbiota-modifying enzyme. Our results shed light on distinct male and female profiles for gut microbiome, IAP, and markers of ME and LGCI that may contribute to sexual dimorphism in MS, revealing new possibilities for preventing and controlling human obesity-related metabolic dysfunction in males and postmenopausal women.

## Results

### Sex differences in ME and LGCI are associated with sexual dimorphism in MS

**Fig. 1:**
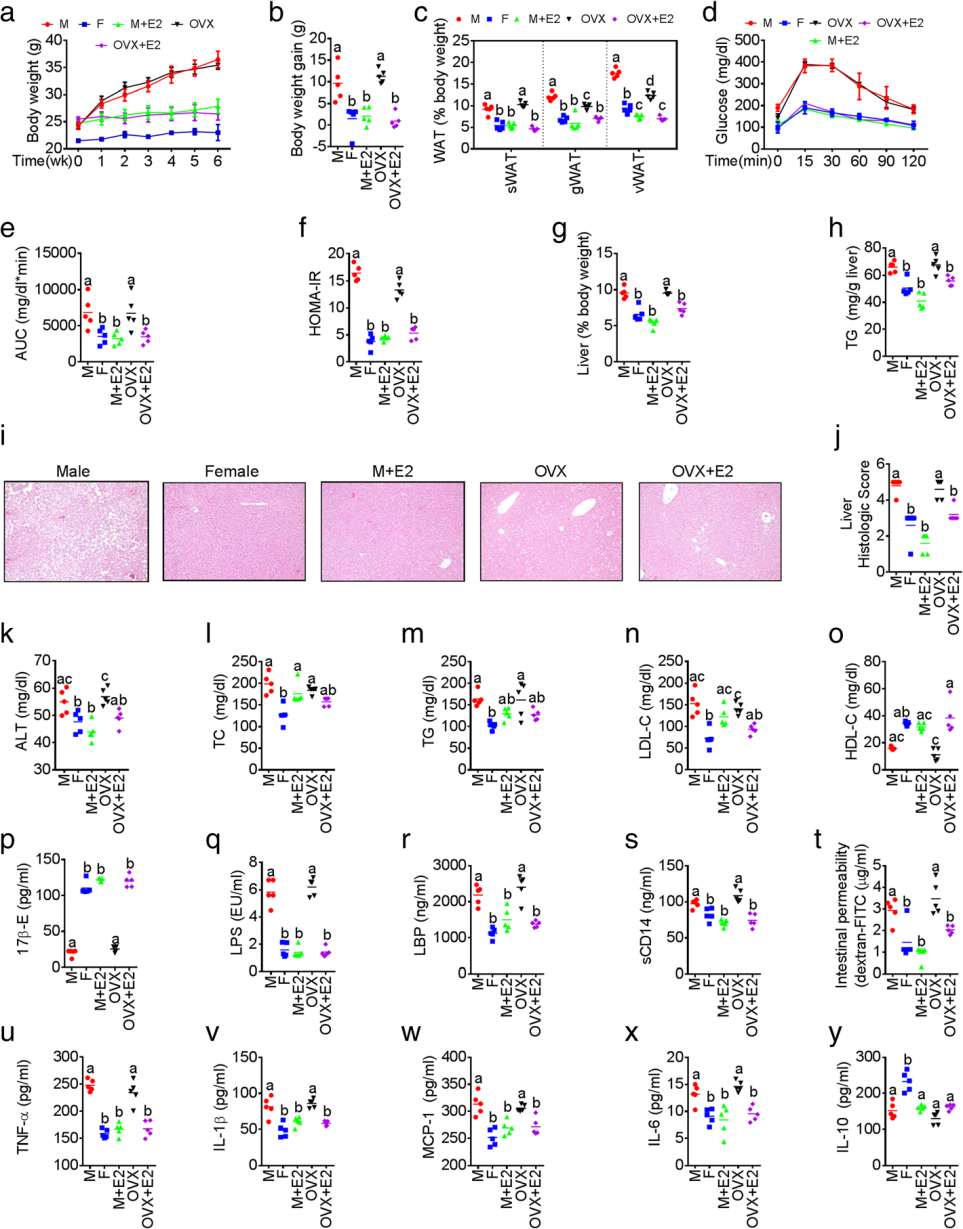
Sexual differences in ME and LGCI are associated with sexual dimorphism in obesity and MS. Male (M), normal female (F), and ovariectomized (OVX) female mice were divided in to five groups (M, F, M+E2, OVX, and OVX+E2; *n* = 5/group) and were fed western diet (WD) ± 17β-estradiol (E2) in the drinking water for 6 weeks. Markers for sexual dimorphism in metabolic syndrome (MS) (**a**–**o**), metabolic endotoxemia (ME) (**q**–**t**), and low-grade chronic inflammation (LGCI) (**u**–**y**) were studied. **a** Weekly body weight. **b** Body weight gain. **c** White adipose tissue (WAT) weight. **d** Glucose tolerance test (GTT). **e** GTT area under curve (AUC). **f** Homeostatic model assessment-insulin resistance (HOMA-IR). **g** Liver weight. **h** Liver triglyceride. **i** Hematoxylin and eosin-stained liver specimens showing histological evidences for fatty liver. **j** Fatty liver score. Serum levels of alanine aminotransferase (ALT) (**k**), total cholesterol (TC) (**l**), triglyceride (TG) (**m**), low-density lipoprotein cholesterol (LDL-C) (**n**), high-density lipoprotein cholesterol (HDL-C) (**o**), E2 levels (**p**), lipopolysaccharides (LPS) (**q**), LPS-binding proteins (LBP) (**r**), and soluble CD14 (sCD14) (**s**). **t** Serum levels of FITC-dextran macromolecules indicating intestinal permeability differences. Serum levels of cytokines such as TNF-α (**u**), IL-1β (**v**), MCP-1 (**w**), IL-6 (**x**), and IL-10 (**y**). Data are expressed as mean ± SE. Box plots (box showing the median, and the 25th and 75th percentiles, and the whiskers of the graph show the largest and smallest values) were used. Data with different superscript letters are significantly different (*P* < 0.05). Ordinary or repeated measures one-way ANOVA followed by Tukey’s multiple comparisons test

We found that relative to males (*n* = 11), female mice (*n* = 11) had significantly lower levels of markers of WD-induced obesity, including gross appearance of mice, intra-abdominal and gonadal fat distribution, body weight gain, and white adipose tissues accumulation) (Additional file 1: Figure S1a-c). Noticeably, although there were no differences in energy intake between males and females (Additional file 1: Figure S1d), relative to males, females had improved MS parameters such as glucose intolerance (glucose tolerance test with area under the curve and HOMA-IR) (Additional file 1: Figure S1e), non-alcoholic fatty liver disease (Additional file 1: Figure S1F-I), and dyslipidemia (total serum cholesterol, triglyceride, HDL-C, LDL-C, and atherogenic index) (Additional file 1: Figure S1j-n). We then investigated whether oral 17β-E (E2) administration ameliorated WD-induced MS in male and ovariectomized (OVX) mice (*n* = 5 per group). Interestingly, MS parameters were more evident in OVX mice compared to normal female (F) mice, and the administration of 17β-E significantly suppressed the development of MS in the male (male+E2) and OVX (OVX+E2) groups (Fig 1a–o and Additional file 1: Figure S1y-z), irrespective of energy intake (Additional file 1: Figure S1x). Notably, there were no significant differences in MS parameters between the female and male+E2 groups (Fig. 1a–o and Additional file 1: Figure S1y-z). This was accompanied with elevated levels of serum 17β-E concentrations in male+E2 and OVX+E2 groups treated with 17β-E compared to control male and OVX groups respectively (Additional file 1: Figure S1p). We investigated whether sexual dimorphism in MS is associated with sex-specific differences in the markers of ME [serum LPS, LPS-binding protein (LBP), soluble CD14 (sCD14), and intestinal permeability] and LGCI [tumor necrosis factor-α (TNF-α), interleukin-1β (IL-1β), IL-6, monocyte chemoattractant protein-1 (MCP-1) and IL-10], and whether estrogen plays a key role in this context. Surprisingly, we found significant differences in the markers of ME (Fig. 1q–t and Additional file 1: Figure S1o-r) and LGCI (Fig. 1u–y and Additional file 1: Figure S1s-w) between WD-fed male and female mice. Lack of endogenous estrogen support in the OVX mice induced significantly elevated levels of markers of ME (Fig. 1q–t) and LGCI (Fig. 1u–y) compared to the female group. Moreover, 17β-E treatment reduced the occurrence of ME and LGCI in the M+E2 and OVX+E2 groups to levels equal to those female mice (Fig. 1q–r).

### Gut microbiome mediates the development of metabolic syndrome in a sex-specific manner

**Fig. 2:**
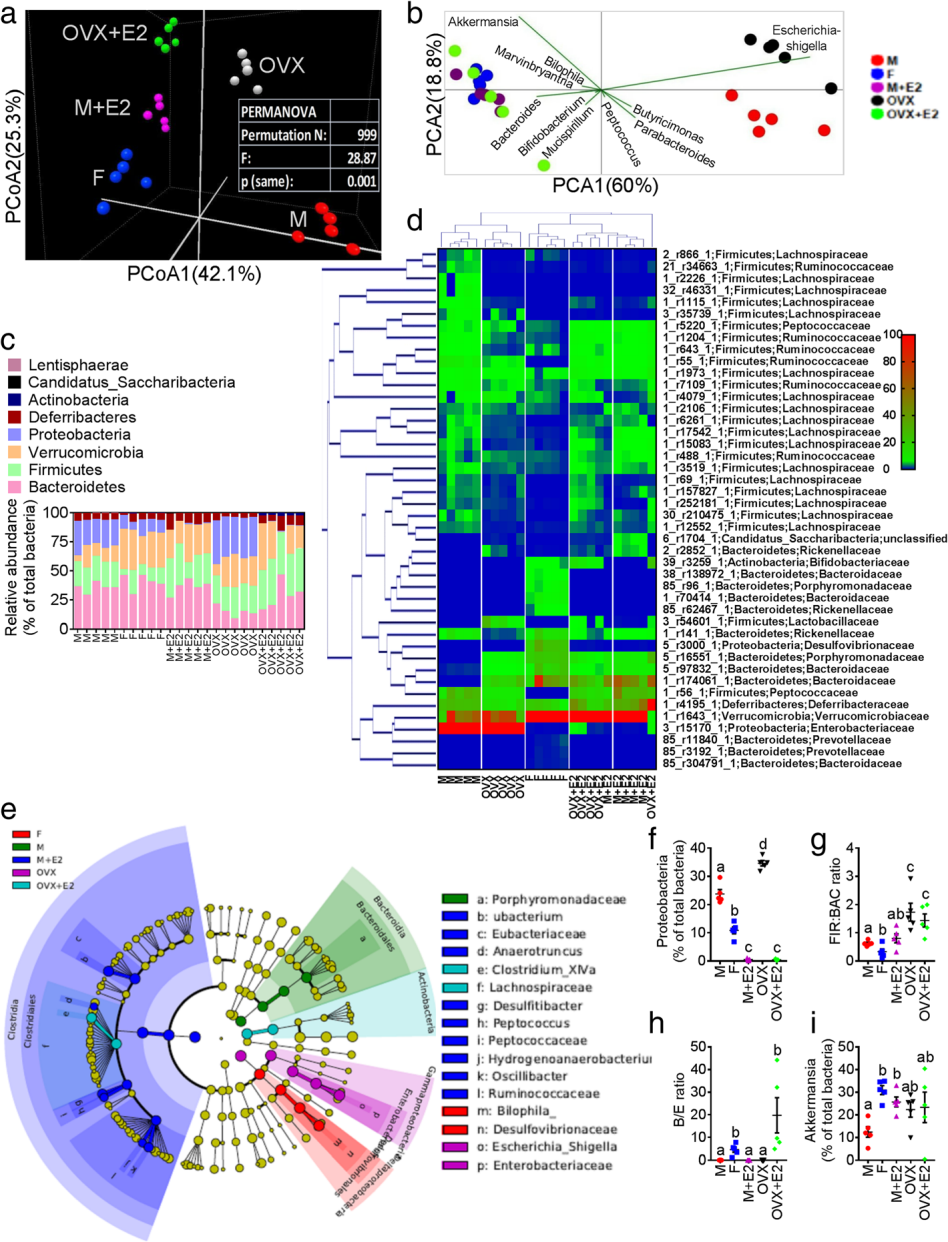
Estrogen alters the gut microbiome. Male (M), normal female (F), and ovariectomized (OVX) female mice were divided in to five groups (M, F, M + E2, OVX, and OVX+E2; *n* = 5/group) and were fed western diet (WD) ± 17β-estradiol (E2) in the drinking water for 6 weeks. **a** β-diversity analysis of whole microbiota relative abundance (RA) using principal coordinate analysis (PCOA) with Bray-Curtis dissimilarity index (BCD) followed by permutational multivariate analysis of variance (PERMANOVA) significance test. **b** Similarity percentage (SIMPER) analysis with BCD was used to identify the specific genera with the greatest contribution to the differences observed between the groups, followed by principal component analysis (PCA) (variance-covariance type) showing the top 10 operational taxonomic unit (OTU) scores included as vectors. The magnitude and direction correspond to the weights. **c** Analysis at the phylum level using RA (%). **d** Hierarchical clustering with a heat map shows the RA of representative OTUs (those with greatest difference between five groups) group means from each family selected for *P* < 0.05, obtained with differential abundance analysis. The OTUs are shown as OTU number, phylum and family. **e** Cladogram generated from linear discriminant analysis (LDA) effect size (LEfSe) showing the relationship between taxon (the levels represent, from the inner to outer rings, phylum, class, order, family, and genus). **f**–**i** Scatter plots showing the *phylum Proteobacteria*, *Firmicutes* (FIR) to *Bacteroidetes* (BAC) ratio, *Bifidobacterium* (B) to *Enterobacteriacea* (E) ratio and RA of *genus Akkermansia* (%). Data was shown as mean ± SEM. Data with different superscript letters are significantly different (*P* < 0.05). One-way ANOVA followed by Tukey’s multiple comparisons test

Given that metabolic endotoxemia is commonly derived from gut dysbiosis, we next used high-throughput 16S rRNA gene sequencing to determine whether sexual dimorphism exist in gut microbiome, and whether 17β-E treatment affected the microbiome composition. V3-V4 16S rRNA gene sequencing was performed on fecal samples collected from M, F, M+E2, OVX, and OVX+E2 groups (*n* = 5 per group) that were fed a WD. Principal coordinate (PCoA) analysis of a common β-diversity index (Bray-Curtis distance) applied on whole microbiota abundance identified a distinct clustering of microbiota composition between male and OVX female and F/OVX+E2/M+E2 groups along the primary ordination axis (axis 1), results are shown in Fig. 2a and Additional file 1: Table S1. Next, taxa which are primarily responsible for an observed difference between groups were analyzed using the SIMPER test (similarity percentage analysis), which identified 10 taxa that were annotated at the genus level. PCoA showed that gram-negative LPS-producing *Escherichia/Shigella* largely contributed to M/OVX groups, whereas LPS-suppressing *Akkermansia muciniphila* [14, 25] and *Bifidobacterium* [14, 26] were associated with the female and 17β-E-treated M/OVX groups (Fig. 2b). At the phylum level, the majority of the bacterial phyla identified in the fecal samples were encompassed by *Bacteriodetes*, *Firmicutes*, *Proteobacteria*, and *Verrucomicrobia*, as shown in Fig. 2c. Furthermore, the relative abundance (RA) of taxa, which showed false discovery rate (FDR)-corrected *P* value < 0.05 with differential expression analysis conducted on whole microbiota profile, were expressed as heat map (Fig. 2d and Additional file 1: Table S2), including hierarchical clustering (HCN). HCN is a clustering technique for graphically summarizing the inter-sample relationships in the form of a dendrogram. HCN also clearly separated the male/OVX samples as a single cluster from the other three groups (F, M+E2, OVX+E2) (Additional file 1: Figure S2a). In order to further identify microbial taxa that serve as biomarkers for different groups, we performed liner discriminate analysis (LDA) coupled with effect size measurements (LEfSe). A cladogram (Fig. 2e) generated from LEfSe analysis showed the relationship between taxon and biomarker taxa (LDA score > 2 and a significance of *P* < 0.05 determined by the Wilcoxon signed-rank test) (Additional file 1: Figure S2b). Notably, in accordance with the SIMPER test, the LPS-producing phylum *Proteobacteria* and its members (*class_γ-Proteobacteria, family_Enterobacteriacea and genus_Escherichia/Shigella*) were higher in the OVX group (Additional file 1: Figure S2b). We also demonstrated that a number of well-studied microbiota-based markers of obesity and MS associated with LGCI were affected by estrogen. These included the *phylum Proteobacteria* [14]; *Firmicutes* to *Bacteroidetes* ratio (FIR/BAC ratio—a known marker of obesity [14, 17]; *Bifidobacterium/Enterobacteriaceae ratio* (B/E)—a well-established marker of colonization resistance to opportunistic pathogens [27]; and the genus *Akkermansia*, which has been shown to reduce fat mass gain and WAT macrophage infiltration and improve gut barrier function and glucose metabolism [28]. Sexual dimorphism was clearly identified with respect to all four markers, with significantly lower *Proteobacteria* abundance, decreased FIR/BAC ratios, higher B/E ratios, and increased *Akkermansia* abundance in normal females compared to normal male and OVX females (Fig. 2f–i). 17β-E treatment significantly reduced *Proteobacteria* and elevated *Akkermansia* in the male (M+E2) (Fig. 2f, i) and significantly reduced the *Proteobacteria* and increased the B/E ratio in OVX females (OVX+E2) (Fig. 2f, h). In addition, resulting taxa identified with pairwise comparison analyses at different taxonomic levels are summarized in the Additional file 1: Table S3. Next, we employed PICRUSt to predict the metagenomes and determine the changes in microbial metabolic pathways (KEGG) across different groups [29]. Male/OVX groups were segregated from the other three groups using PCoA applied on predicted functional pathways (Additional file 1: Figure S2c). According to LEfSe analysis, LPS-related functional pathways were significantly lower in the female, M+E2 and OVX+E2 groups compared to male and OVX groups (Additional file 1: Figure S2d-f). The α-diversity profile for groups reached stable values as indicated by the observed plateaus seen for each group in the Rarefaction curves (Additional file 1: Figure S3a-e). Together, these results highlight the sex specific shifts in gut microbiome that occurred upon WD and 17β-E treatment.

**Fig. 3:**
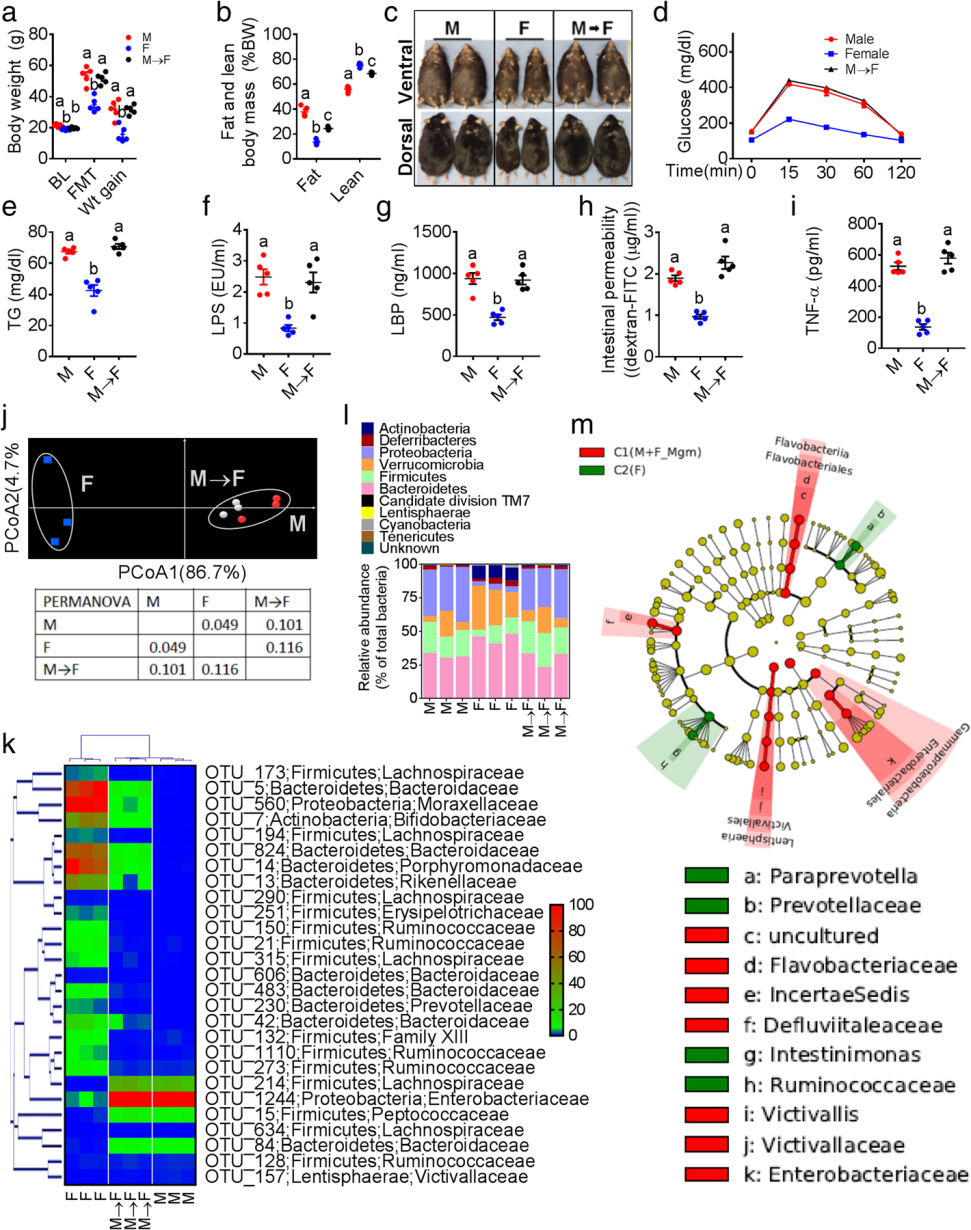
Male mice fecal microbiota-transplant transfers MS to female recipients. Fecal microbiota transplantation (FMT) with fecal content from donor male (M; *n* = 5) mice fed WD was performed on female recipient mice (F → M; *n* = 5) pretreated with an antibiotic cocktail. Normal female (F; *n* = 5) mice were maintained as a control group. **a**–**e** Markers of obesity (baseline (BL) and after FMT body weight and body weight gain in the end (**a**), fat and lean body mass (% body weight) (**b**), gross appearance (dorsal and ventral aspects have been shown to differentiate the female from male sex) of male, female, and F → M mice (**c**), glucose tolerance test results (**d**), and serum triglyceride levels ®. **f**–**h** Markers of metabolic endotoxemia such as serum lipopolysaccharide (LPS) concentration (**f**) and LPS-binding proteins (LBP) concentrations (**g**) and intestinal permeability to FITC-dextran macro molecules (**h**). **i** Serum TNF-α. **j** β-diversity analysis using principal coordinate analysis (PCOA) with Bray-Curtis dissimilarity index followed by PERMANOVA significance test. **k** Hierarchical clustering with a heat map shows the representative OTU (those with greatest difference between groups) group means from each family selected for *P* < 0.05, obtained with differential abundance analysis. **l** Phylum-level analysis using the relative abundance (RA). **m** Cladogram generated from LEfSe analysis showing the relationship between biomarker taxa (the levels represent, from the inner to outer rings, phylum, class, order, family, and genus). *®*®Data was shown as mean ± SEM. Data with different superscript letters are significantly different (*P* < 0.05). Ordinary or repeated measures one-way ANOVA followed by Tukey’s multiple comparisons test

We next used fecal microbiota transplantation (FMT) and antibiotic (ABX)-induced depletion to investigate whether gut microbiota were necessary to mediate sexual dimorphism in MS. FMT with fecal content from donor male mice fed WD for 10 weeks was performed on female 10-week-old recipient mice pretreated with an antibiotic cocktail [19]. Unexpectedly, male mice fecal-transplant induced obesity (Fig. 3a–c) and glucose intolerance (Fig. 3d and Additional file 1: Figure S3f) and elevated serum TG (Fig. 3e), and markers of ME (Fig. 3f–h) and LGCI (Fig. 3i) in female recipients, with no differences between male and M → F groups (*n* = 5 per group). Moreover, fecal β-diversity microbiota analysis (Fig. 3j) and HCN with heat-map analysis (Fig. 3k) showed that the M and M → F samples were clustered together and were separated from the female control group (*n* = 3 per group). M and M → F groups presented with higher *Proteobacteria* and *Firmicutes* and lower *Verrucomicrobia* and *Bacteroidetes* compared to females (Fig. 3l). LEfSe showed *γ-Proteobacteria* and *Enterobacteriacea* for M and M → F groups analyzed as a single cluster (Fig. 3m and Additional file 1: Figure S3g). Higher RA of both *Proteobacteria* and FIR/BAC ratios, and lower B/E ratio and *Akkermansia muciniphila* abundance (Additional file 1: Figure S2g-j) were also observed with the M → F group, while there were no significant differences in the α-diversity measures (Additional file 1: Figure S3h-l). Principal component analysis (PCA) with predicted metabolic pathways clustered the M and M → F groups (Additional file 1: Figure S2k), and significantly higher abundance of LPS-related functions were found with LEfSe analysis (Additional file 1: Figure S2l). Finally, all of the abovementioned results were associated with undetectable (< 6.6 pg/mL) serum E2 levels in the M → F(Additional file 1: Figure S3m) group after FMT.

**Fig. 4:**
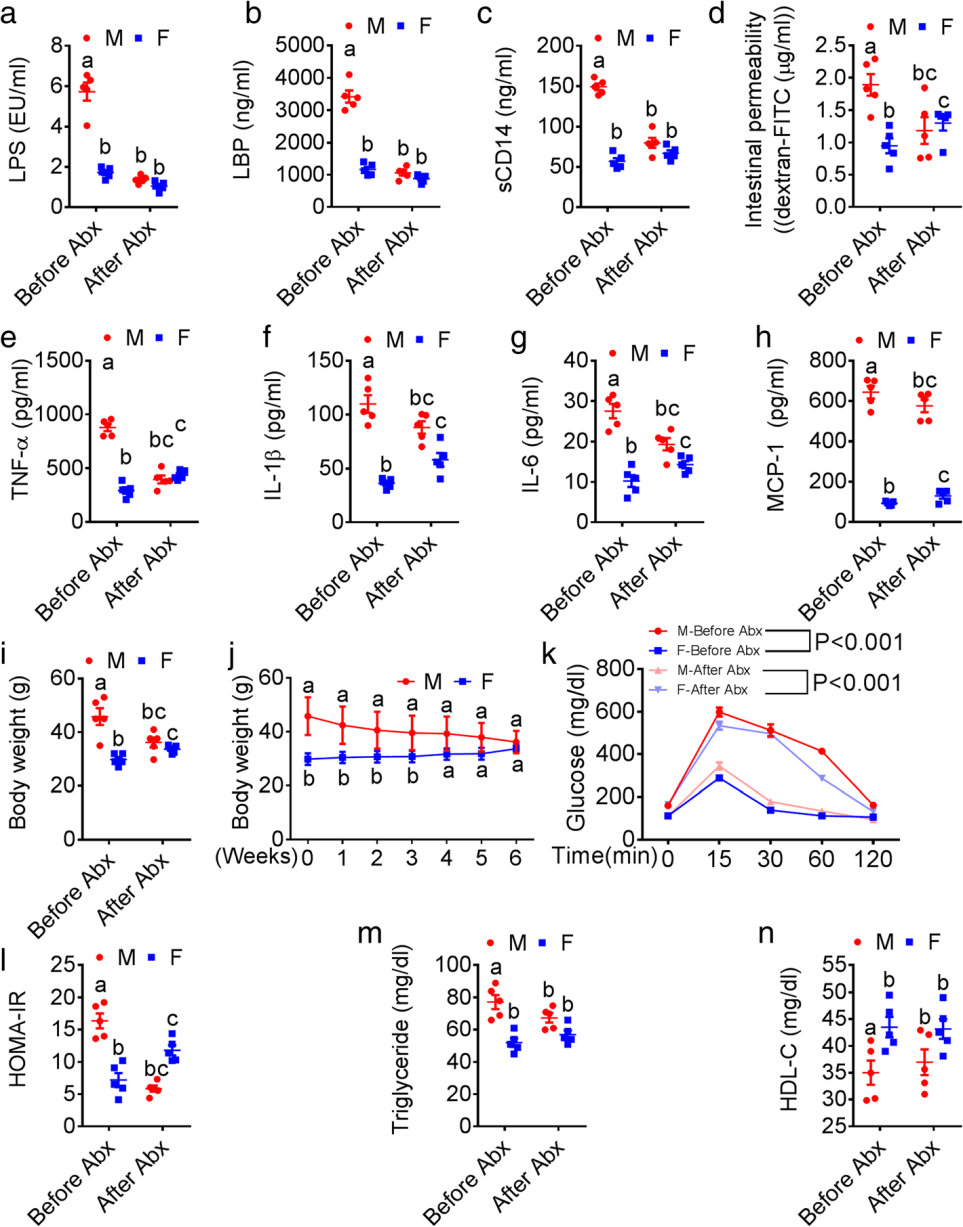
Antibiotic treatment alleviates sexual dimorphism in MS. 10-week-old male (*n* = 5) and female (*n =* 5) mice maintained on chow diet were switched to a WD until the age of 20 weeks to induce sexual dimorphism in MS. Then, a broad-spectrum antibiotic cocktail (ABX) containing ampicillin (1 g/L), vancomycin (500 mg/L), neomycin sulfate (1 g/L) (added to drinking water), and metronidazole (100 mg/kg) (orally gavaged every 12 h) was administered for next 6 weeks to deplete the gut microbiota. **a**–**d** Sexual dimorphism in markers of metabolic endotoxemia (serum LPS, LBP, sCD14, and intestinal permeability). **e**–**h** Markers of low-grade chronic inflammation (TNF-α, IL-1β, IL-6, MCP-1, and IL-10). **i**–**n** Features of metabolic syndrome (body weight, glucose tolerance test, insulin resistance (HOMA-IR), serum triglycerides and HDL-C). Data (M vs. F and Before Abx vs. After Abx) were analyzed at the baseline (after 20 weeks of WD feeding) and after ABX treatment. Data was shown as mean ± SEM. Data with different superscript letters are significantly different (*P* < 0.05). Ordinary or repeated measures two-way ANOVA followed by Sidak’s multiple comparisons test

To further explore the causative role of gut microbiota, we next depleted the gut microbiota [14] in mice using a well-established ABX cocktail. Male and female mice (*n* = 5/group) were fed WD for 10 weeks to induce MS. As expected, we found appreciable sexual dimorphism in the markers of ME (Fig. 4a), LGCI (Fig. 4b and Additional file 1: Figure S4a), and MS (Fig. 4c and Additional file 1: Figure S4b-d). Mice then received the ABX cocktail in drinking water for next 6 weeks. Strikingly, in addition to elimination of sexual dimorphism in markers of ME (Fig. 4a–d), LGCI (Fig. 4e–h and Additional file 1: Figure S4a), and MS (Fig. 4i–n and Additional file 1: Figure S4b-d), female mice showed significantly elevated levels of gut permeability (Fig. 4d), LGCI (Fig. 4e–h), body weight gain (Fig. 4i–j), glucose intolerance (Fig. 4k and Additional file 1: Figure b), HOMA-IR (Fig. 4i), and serum TG (Fig. 4m). Interestingly, female mice showed significantly lower serum E2 levels after ABX treatment compared to before ABX treatment (Additional file 1: Figure S4e). In combination, the results of these FMT and ABX experiments suggest that the gut microbiome mediates sexual dimorphism in the MS mainly through regulating the development ME and LGCI.

### Isoflavones produce microbiome modifying effects similar to estrogen and reverse MS in male mice

**Fig. 5:**
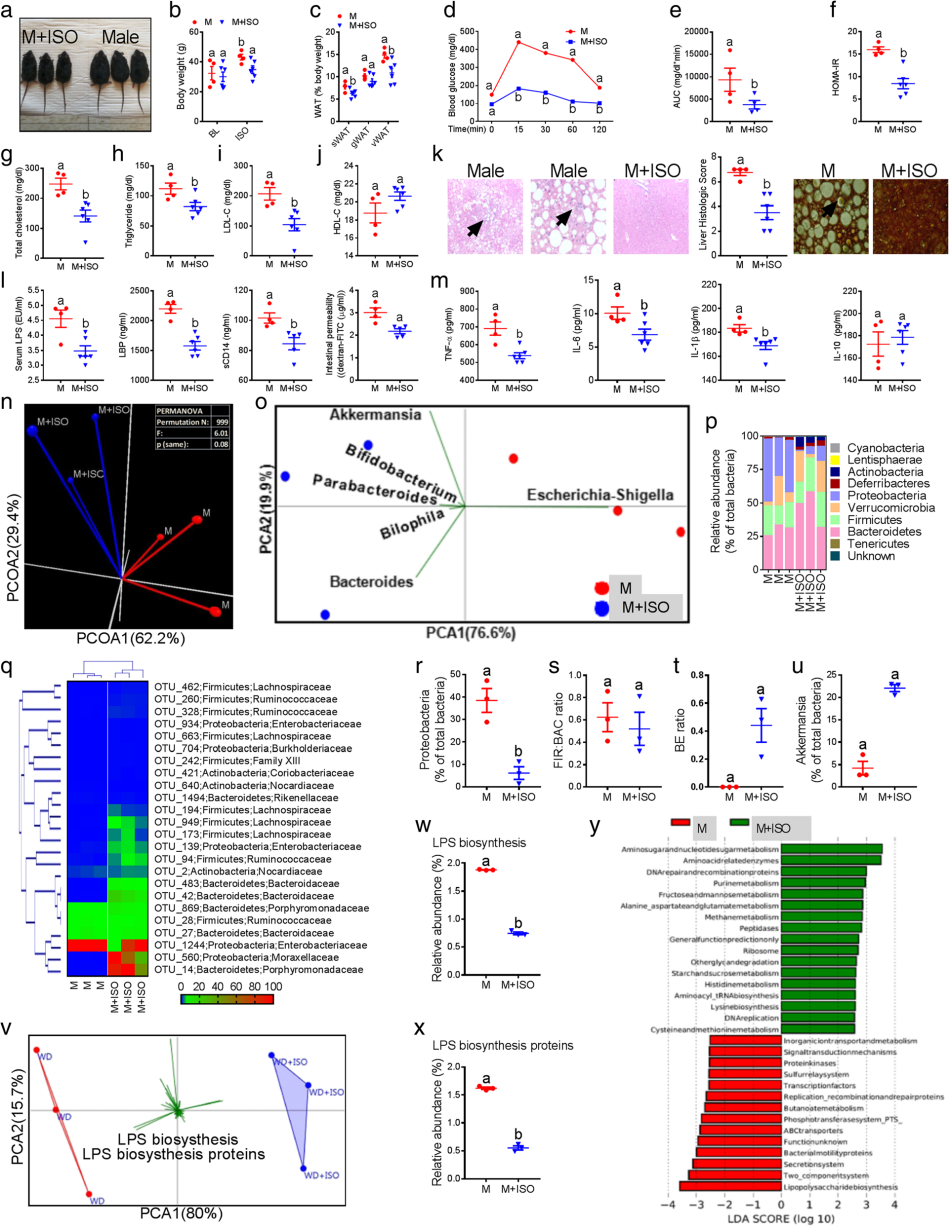
Isoflavones produce similar microbiome-modifying effects as estrogens and reverse MS. Male mice with obesity and metabolic syndrome features were divided into Male (*n* = 4) and Male+ISO (*n* = 6) groups. Male+ISO group received supplementation of both genistein (G) and daidzein with a WD. **a**–**k** Metabolic syndrome markers such as gross appearance and body weight (**a**-**b**) and white adipose tissue (WAT) weight (**c**), glucose tolerance test curves with area under the curve (AUC) (**d**, **e**) and insulin resistance (HOMA-IR) (**f**), serum total cholesterol, triglycerides, LDL-C and HDL-C (**g**–**j**) histological evidences showing non-alcoholic fatty liver disease features (arrow heads indicating inflammatory cells infiltration, Mallory bodies and mild fibrosis) (**k**). **l** Markers of metabolic endotoxemia (serum LPS, LBP, sCD14, and intestinal permeability). **m** Markers of low-grade chronic inflammation (TNF-α, IL-6, IL-1β, and IL-10). **n** β-diversity analysis using principal coordinate analysis (PCOA) with Bray-Curtis dissimilarity index (BCD) followed by PERMANOVA significance test. **o** SIMPER with BCD analysis followed by principal component analysis (PCA) (variance-covariance type) showing the top six operational taxonomic unit (OTU) scores included as vectors. **p** Analysis at the phylum level using RA (%). **q** Hierarchical clustering with a heat map shows the relative abundance (RA) of representative OTUs (those with greatest difference between groups) group means from each family selected for *P* < 0.05, obtained with differential abundance analysis. **r**–**u** Box plots showing the *Proteobacteria*, *Firmicutes* (FIR) to *Bacteroidetes* (BAC) ratio, *Bifidobacterium* (B) to *Enterobacteriacea* (E) ratio, and RA of genus *Akkermansia*. **v** RA of predicted microbial genes was identified using PICRUSt analysis followed by PCA (variance-covariance type) and the resulting scores were included as vectors. **w**, **x** RA of lipopolysaccharide (LPS) biosynthesis and LPS biosynthesis proteins. **y** LDA scores derived from LEfSe analysis conducted on predicted microbial genes identified by PICRUSt, showing the biomarker functions. Data was shown as mean ± SEM. Data with different superscript letters are significantly different (*P* < 0.05). Student’s *t* test or repeated measures one-way ANOVA followed by Tukey’s multiple comparison test

To explore whether estrogen-like compounds can produce similar effects to those we found in the 17β-E-treated male, we used isoflavones (ISO) such as genistein (G) and daidzein (D). Male mice (*n* = 10) were fed WD for 4 months to induce severe obesity and MS and then treated with or without G and D isoflavones for 5 weeks. Interestingly, ISO treatment significantly reversed the WD-induced obesity (Fig. 5a–c), glucose intolerance and HOMA-IR (Fig. 5d–f), dyslipidemia (Fig. 5g–j), and NAFLD (including formation of Mallory bodies and mild fibrosis induced by WD) (Fig. 5k) despite similar energy intake between the male (*n* = 4) and male+ISO (*n* = 6) groups (Additional file 1: Figure S5a). As expected, the reversal of MS parameters was associated with lower levels of markers of ME (Fig. 5l) and LGCI (Fig. 5m). Furthermore, we found higher mRNA levels of estrogen receptor (ER)-α and no changes with ER-β in the duodenal tissues of ISO-treated male mice (Additional file 1: Figure S5b-c). Moreover, principal coordinate β-diversity analysis applied on entire fecal microbiome population distinctly clustered the Male+ISO and Male samples on axis one (Fig. 5n). Interestingly, similar to 17β-E, SIMPER test showed a higher contribution of *Escherichia-Shigella* to the M group whereas *Akkermansia, Bifidobacterium*, and *Bacteroides* were associated with the M+ISO group (Fig. 5o). At the phylum level, ISO treatment significantly reduced the RA of *Proteobacteria* and increased *Actinobacteria* and *Verrucomicrobia* (Fig. 5p). Differential expression with HCN analysis also grouped the M+ISO samples and the RA of major bacterial groups as shown in the heat map (Fig. 5q). In addition, significantly lower *Proteobacteria* levels (Fig. 5r), higher B/E ratios (Fig. 5t), increased *Akkermansia* levels (Fig. 5u), and significantly greater α-diversity measures (Additional file 1: Figure S5b-f) were observed in the M+ISO group, although no differences were observed with FIR/BAC ratios (Fig. 5s). PCA of predicted functional metabolic pathways also segregated the M+ISO group from the male samples (Fig. 5v). In addition, the RA of LPS-related functional pathways were significantly lower in M+ISO group compared to male group (Fig. 5w, x), which was also confirmed by LEfSe analysis (Fig. 5y). These data suggest that dietary supplementation of phytoestrogens such as ISO may exert effects similar to those of 17β-E on the microbiome, ME, and LGCI in males to prevent MS.

### Intestinal alkaline phosphatase (IAP) drives sexual dimorphism in gut microbiota

**Fig. 6:**
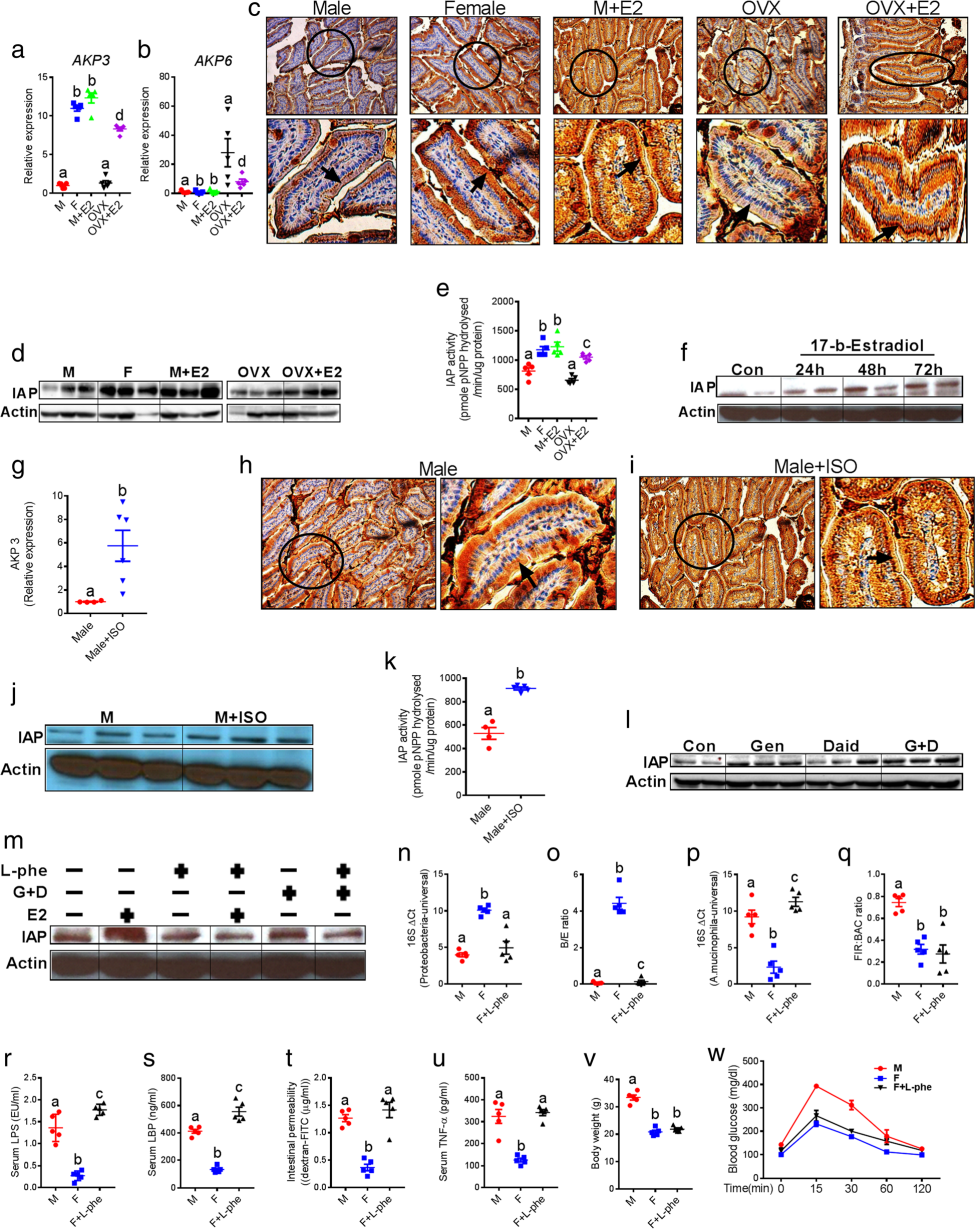
Intestinal alkaline phosphatase (IAP) mediates sexual dimorphism in gut microbiota in the context of MS. **a**, **b** mRNA expression of two major IAP isozymes AKP3 and AKP6 in the duodenum of five groups (male, female, male+E2, OVX, and OVX+E2; *n =* 5 per group). **c** Immunohistochemical (IHC) localization of IAP in the duodenum. The enlarged view of single villus (circled in the main figures) has also been placed under each picture with an arrow head indicating the location of IAP in a circular fashion in the tip of small intestinal villus. **d** Western blot (WB) analysis showing the protein levels of IAP. **e** Duodenal tissue total IAP activity. **f** WB analysis of IAP using cell lysates collected from differentiated Caco-2 cell with enterocytes-like features after stimulating them (in vitro) with 17β-estradiol (10 nM) in a time-dependent manner. **g** AKP3 mRNA expression of male (M) and male +ISO (M+ISO) groups. **h**, **i** Representative IHC staining pictures showing the IAP expression. **j** WB analysis showing the protein levels of IAP. **k** Total IAP activity. **l** In vitro stimulation of Caco-2 cells with 25 μM concentration of either genistein (G) or daidzein (D) or G+D combination, followed by WB analysis of IAP. **m** In vitro stimulation of Caco-2 cells in the presence or absence of L-phenylalanine (10 mM), G+D (25 μM), and 17β-E (10 nM), followed by WB analysis of IAP. q-PCR-based quantification of *Proteobacteria*, *Bifidobacterium* (B) to *Enterobacteriacea* (E) ratio *and* relative abundance of *Akkermansia* and *Firmicutes* (FIR) to *Bacteroidetes* (BAC) ratio (**n**–**q**), markers of metabolic endotoxemia (serum LPS, LBP and intestinal permeability) (**r**–**t**), low-grade chronic inflammation (TNF-α) (**u**), and markers of metabolic syndrome (body weight and GTT) (**v**, **w**) in the male (M), female (F), and F+L-phe (10 mM)-treated groups. Data was shown as mean ± SEM. Box plots (box showing the median, and the 25th and 75th percentiles, and the whiskers of the graph show the largest and smallest values) were also used. Data with different superscript letters are significantly different (*P* < 0.05). Student’s *t* test or ordinary or repeated measures one-way ANOVA followed by Tukey’s multiple comparisons test

It has been well established that endogenous antimicrobial peptides (eAMP) (e.g., IAP and Reg3γ) maintain normal gut homeostasis and regulate gut microbiota composition [14, 30]. Next, we examined the extent to which sexual dimorphism in gut microbiome was driven by differences in AMP levels between males and females, and whether estrogen status affected endogenous eAMP production. Among the eAMPs analyzed (Fig. 6a, b and Additional file 1: Figure S5i-j), we found significant differences only in IAP between groups based on sex and estrogen treatment (Fig. 6a, b). Recently, we and others have shown the gut microbiome-modifying effects of intestinal alkaline phosphatase (IAP), especially with respect to LPS-related and gram-positive bacterial groups [14, 31–33]. In addition to in vitro cell culture studies, duodenal tissues of the study groups (Fig. 2) were analyzed for IAP after 6 weeks of 17β-E treatment. We found upregulated mRNA expression levels of *Akp3* (a major IAP isozyme) (Fig. 6a), downregulated expression of the *Akp6* IAP isozyme (Fig. 6b), higher villus-associated enterocytes expression of IAP with immunohistochemical staining (Fig. 6c), elevated IAP protein levels (Fig. 6d), and elevated IAP enzymatic activity in the duodenum (Fig. 6e) of female and 17β-E-treated male and OVX groups compared to normal male and OVX mice. Moreover, the stimulation of differentiated Caco2 cells with 17β-E (10 nM) significantly elevated IAP levels in a time-dependent manner (Fig. 6f). Similarly, elevated mRNA expression of *Akp3* (Fig. 6g, with no detection of expression of *Akp6)*, higher immunochemical staining of IAP (Fig. 6h, i), elevated IAP protein levels (Fig. 6j), and elevated IAP activity (Fig. 6k) were observed in the duodenum of M+ISO group compared to male group after 5 weeks of ISO treatment. In addition, in vitro stimulation of Caco2 cells with 25 μM concentration of either genistein (G) or daidzein (D) or G+D combination also resulted in increased IAP protein levels (Fig. 6l). We also examined the effects of IAP inhibition using L-phenylalanine (L-phe), a specific non-competitive inhibitor [14, 32, 34, 35] of IAP. L-phe (10 mM) pretreatment of Caco2 cells blocked the elevation of IAP protein found with 17β-E and ISO treatment (Fig. 6m). Further analysis using samples collected from in vivo (17β-E and ISO experiments in mice) and in vitro (Caco-2 cells) experiments indicated that both 17β-E and ISO significantly upregulated the mRNA levels of nuclear transcription factor KLF4 (gut-enriched Krüppel-like factor) (Additional file 1: Figure S5k-l). There was a clear trend of increased mRNA levels of Cdx1 (caudal-type homeobox-1) transcription factor (Additional file 1: Figure S5m-n), both KLF4 and Cdx1 are major transcription factors that target IAP [36]. To further verify whether IAP is one of the primary factors responsible for the differences in the gut microbiota between male and female C57BL/6 mice, we examined the effects of IAP inhibition on the status of key microbiota changes. After 8 weeks of WD and L-phe (10 mM) treatment, we found that the L-phe-treated female mice exhibited a significant increase in LPS-producing *Proteobacteria*, decrease of B/E ratio, increase of the relative abundance (RA) of *Akkermansia muciniphila* and no differences in FIR/BAC ratio (Fig. 6n–q) compared to the untreated female (F) group (*n* = 5 per group). Accordingly, the L-phe-treated female group showed significantly higher levels of markers of metabolic endotoxemia (Fig. 6r–t), LGCI (Fig. 6u), and glucose intolerance (Fig. 6v with no differences in body weight gain (Fig. 6w). These results support the notion that sexual dimorphism in endogenous IAP activity might partially drive differences in gut microbiota between male and female, and 17β-E and ISO-treated C57BL/6 mice.

### Network interactions reveal host-microbiome interactions (HMI) driven by estrogen status

**Fig. 7:**
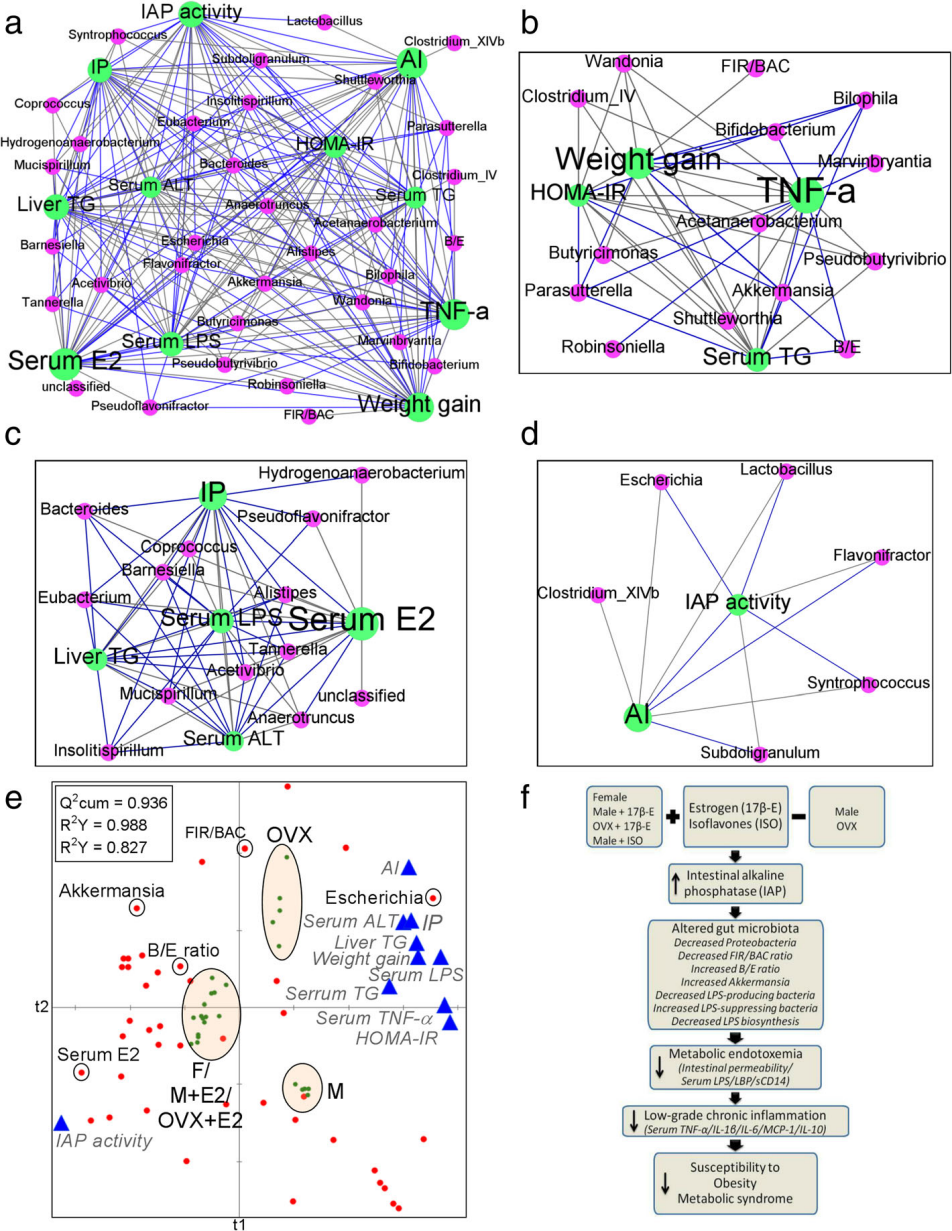
Network interactions reveal host-microbiome interactions (HMI) driven by estrogen status. **a**–**d** The HMI network (**a**) was first built from Spearman’s nonparametric rank correlation coefficient (*P* < 0.05) between host parameters and microbial genera. Each node was colored according to the “data type” and sized based on “betweenness centrality,” which quantifies the influence of a node in connecting other nodes within network values. Edges (lines) represent statistically significant correlations and are colored light black for positive and blue for negative correlations. Next, nodes were grouped as modules (a set nodes connected to each other by many links, while connected by few links to nodes of other groups) and the first three largest modules of the network were taken to show the relationship between specific genera and host parameters (**b**-**d**). **e** Partial least square (PLS) regression loading score plot. The plot illustrates the association of between host parameters (dependent variables colored blue) and microbial genera (explanatory variables colored red). Samples from five different groups were observations (green dots). Leave one-out cross-validation (LOO-CV) was applied. The global goodness of fit (Q^2^cum) and the predictive quality of the models (R^2^Y and R^2^X cum) values were inserted on the top left corner of the figure. **f** Diagram illustrating a proposed mechanism. Endogenous estrogens in the female mice, exogenous supplementation of 17β-estradiol (17β-E) to male (M+17β-E) and ovariectomized female (OVX+17β-E) mice and dietary supplementation of isoflavones (ISO) to male mice upregulate nuclear transcription factors (KLF4) that target IAP, which, in turn, increases the endogenous IAP activity in the gut. Elevated IAP activity leads to decreased *Proteobacteria* and *Firmicutes* to *Bacteroidetes* ratio (FIR/BAC), increased *Bifidobacterium* to *Enterobacteriacea* ratio (B/E), and the abundance of *Akkermansia* genera, decreasing LPS-producing bacteria (e.g., *Proteobacteria*) while increasing LPS-suppressing bacteria (e.g., *Bifidobacterium* and *Akkermansia mucinophila*). These changes lower LPS production (LPS biosynthesis and related proteins) and intestinal permeability, resulting in reduced metabolic endotoxemia (serum LPS/LPS binding proteins/soluble CD14). The subsequent reduction of inflammatory cytokines leads to the suppression of low-grade chronic inflammation and metabolic syndrome. AI, atherogenic index; TG, triglyceride; IAP, intestinal alkaline phosphatase; E2, 17β-estradiol

Integrating significant microbial associations detected from OTU tables with metadata measurements of interest using correlation network analysis not only will provide us with valuable insights into the dynamics of the interactions between external factors and the microbial community, but also can help us understand how the detected relationships might change when additional variables are taken into account [37]. The HMI network (Fig. 7a) was first built from Spearman’s nonparametric rank correlation coefficient (*P* < 0.05) between host parameters and microbial genera. Each node was colored according to the “data type” and sized based on “betweenness centrality,” which quantifies the influence of a node in connecting other nodes within network values. Edges (lines) represent statistically significant correlations, and are colored light black for positive and blue for negative correlations. Next, nodes were grouped as modules and the first three largest modules of the network were taken to show the relationship between specific genera and host parameters (Fig. 7b, d). Partial least square (PLS) regression loading score plot (Fig. 7e) illustrates the association of between host parameters (dependent variables colored blue) and microbial genera (explanatory variables colored red). Samples from five different groups were observations (green dots). Accordingly, a diagram illustrating a proposed mechanism has been developed (Fig. 7f). Endogenous estrogens in the female mice, exogenous supplementation of 17β-E to male and ovariectomized female mice, and dietary supplementation of ISO to male mice upregulate KLF4 transcription factor that target IAP, which, in turn, increases the endogenous IAP activity in the gut. Elevated IAP activity leads to decrease *Proteobacteria* and FIR/BAC, increase B/E, and the abundance of *Akkermansia* genera, decreasing LPS-producing bacteria (e.g., *Proteobacteria*) while increasing LPS-suppressing bacteria (e.g., *Bifidobacterium* and *Akkermansia mucinophila*). These changes lower LPS production and IP, resulting in reduced ME. The subsequent reduction of inflammatory cytokines leads to the suppression of LGCI and MS.

## Discussion

To date, the mechanisms underlying the reported sexual dimorphism in MS have remained enigmatic. The present study demonstrates for the first time that sex-dependent effects on the gut microbiome mediate sexual dimorphism in MS in C57BL/6 mice and sex-specific expression and activity of IAP, a major gut microbiota-modifying factor [31–33]. Given the causative role of ME originating from dysbiotic gut microbiota and LGCI induced by ME in the context of WD-induced obesity and MS [14–16], the novel findings in our study are as follows: (1) sex-specific differences in the gut microbiota composition (e.g., *Proteobacteria*, FIR/BAC ratio, B/E ratio, and *Akkermansia*) and functions (e.g., LPS biosynthesis and LPS-related proteins) and markers of ME and LGCI induced by WD are associated with sexual dimorphism in MS. (2) Male mice are markedly susceptible to ME and LGCI and female mice are exclusively protected from ME and LGCI. (3) Gut microbiota, especially LPS-related bacteria, mediate the sexual dimorphism in MS, reflected by the fact that male mice microbiota transplants induced ME, LGCI, and MS in the female recipients and that ABX abolished the sexual dimorphism and worsened the MS markers in female mice. (4) 17β-E induces gut microbiome changes, which is associated with lower susceptibility to WD-induced ME, LGCI, and MS in the male and OVX mice. Moreover, 17β-E-induced gut microbiome changes and protection against MS are associated with elevation of activity and expression of IAP. (5) Estrogen-like compounds (e.g., isoflavones) had gut microbiome-modifying effects similar to estrogens and prevented WD-induced ME, LGCI, and MS in male mice by elevating IAP, indicating that dietary supplementation of isoflavones could be a potential alternative to 17β-E to treat men and postmenopausal women who are affected by obesity and MS. (6) IAP could largely mediate the 17β-E and ISO-induced gut microbiome changes, and this might be due to the E2-mediated upregulation of transcription factors that target IAP such as KLF4 and CDX1.

It is important to note that the major gut microbiota findings (changes in *Proteobacteria,* FIR/BAC ratio, B/E ratio, and *Akkermansia*) reported here regarding sexual dimorphism have been studied extensively in animals and also in the humans in the context of obesity and MS [17–19]. Data obtained from animal models identified consistent differences in the two major bacterial phyla, with a significant increase in *Firmicutes* and decrease in *Bacteroidetes* levels in genetically obese mice compared to wild-type mice despite similarities in their diet and activity levels [16]. Consistent with pre-clinical data, numerous human studies have consistently demonstrated that the FIR/BAC ratio is specifically increased in obese people [17–19]. Metabolic endotoxemia derived from gut dysbiosis is central to the pathogenesis of chronic low-grade inflammation, a factor underlying many current chronic diseases [9, 22]. Metabolic endotoxemia can be determined by the abundance of bacteria affecting LPS production and gut barrier function. It is therefore conceivable that the 17β-E-induced marked reductions in LPS-producing bacteria (e.g., *Proteobacteria*) and increases in LPS-suppressing bacteria (e.g., *Bifidobacterium* and *A. mucinophila*) [14, 25–27] significantly suppressed the development of endotoxemia and inflammation. Recent clinical studies have shown that LPS-producing bacteria are abundant in obese subjects with type 2 diabetes [14, 16, 38–40]. It has also been shown that male mice fed a WD rich in milk fat and omega-6 fatty acids exhibit overgrowth of LPS-producing *Proteobacteria* and reduction of LPS-suppressing *Bifidobacterium spp* [27, 41, 42]. Along these lines, the WD diet used in the present study induced a dramatic increase in *Proteobacteria* and a decrease in *Bifidobacterium* in male and OVX mice (Fig. 3 and 5). In this context, decreasing the abundance of LPS-producing bacteria and increasing the LPS-suppressing bacteria may be a key mechanism for the reduction of metabolic endotoxemia. Another potential mechanism contributing to the reduction of serum LPS may be a decrease in gut permeability, due to the observed elevation of gut barrier-protecting bacteria such as *Bifidobacterium* [14, 43] by estrogen in our study.

In postmenopausal women and in female animal models, lower estrogen levels are associated with increased visceral adiposity [10] and estrogen replacement improves glucose-insulin homeostasis [44]. We have shown here for the first time the effects of E2 replacement on gut microbiota and metabolic endotoxemia in OVX mice, which mimics the postmenopausal state. There is growing evidence for a fundamental role of estrogen in the regulation of obesity and related metabolic disorders in males [11, 45], and recent data from rodent studies suggest that hepatic estrogen signaling has a key role in the prevention of high-fat diet-induced insulin resistance in males. However, it is not known whether estrogen treatment in males protects MS by modulating gut microbiota. Our novel results show that estrogen treatment in males is associated with the modulation of gut microbiota and improvement in ME and LGCI, which is associated with improvements in weight management and obesity-induced metabolic changes (Fig. 7), supporting the concept that estrogen plays an important role in the control of serum LPS levels by affecting LPS-related gut microbiota.

IAP is an endogenous antimicrobial peptide with numerous physiological functions [46, 47]. It is highly expressed in the small intestine, secreted from apical enterocytes into the lumen in microvilli vesicles, and travels to the large intestine [48]. IAP is known to inhibit the growth of *E. coli* and gram-negative bacteria by dephosphorylating LPS located in the outer membrane [32, 48–51]. IAP is also able to dephosphorylate ATP [33], which has been shown to reduce the survival of gram-positive bacteria (46), and to support the growth of gram-negative bacteria such as *E. coli* [52]. Oral IAP supplementation has also been shown to prevent *E. coli* overgrowth [53]. We recently found in our fat-1 mice model (transgenic mice with elevated tissue n-6/n-3 fatty acid ratio) that elevated endogenous IAP activity and expression is associated with lower levels of LPS-producing bacteria and higher levels of LPS-suppressing bacteria [14]. Moreover, we found that inhibition of IAP by phenylalanine, a frequently used specific inhibitor of endogenous IAP activity [14, 15, 34, 35], caused ME, LGCI, and MS by increasing the growth of LPS-producing *Proteobacteria* and reducing the growth of LPS-suppressing *Bifidobacterium spp* in a mouse model of elevated endogenous IAP activity [14, 15]. It is clear that IAP expression in the intestine is a critical determinant of the gut microbiota profile. Our findings indicate that IAP may be a primary factor partially mediating the effects of estrogen on gut microbiota because IAP inhibition in the female mice led to the development of ME, LGCI, and MS, associated with dramatic increase of *Proteobacteria* and reduction of the B/E ratio and *A. mucinophila* abundance (Fig. 6n–q). The mechanism by which estrogen may modulate IAP expression is possibly due to its regulatory effect on the KLF4 transcription factor, which has been shown to target IAP [36].

Isoflavones (ISO) are non-steroidal compounds that can bind to both ER-α and ER-β due to their ability to mimic the conformational structure of estradiol [54, 55], and thereby imitate the actions of estrogens on target tissues [56]. Isoflavones are found in many legumes and are particularly abundant in soy products. Genistein (G) and daidzein (D), two major soy isoflavone glucosides, are present at high concentrations in soybeans and soybean-derived products and are a major source of xenoestrogen exposure in both humans (e.g., soy-based formula for infants, tofu) and animals (most commercially available diets). G and D are widely used as dietary supplements in the USA for various presumed health benefits [57]. Ferguson et al. [58] administered a low-dose endotoxin (LPS 1 ng/kg) to induce postprandial transient endotoxemia in young, healthy volunteers and found that subjects with a high-isoflavone diet were protected against inflammation-induced decline in insulin sensitivity. Meals high in fat, or fat and simple carbohydrates, are known to induce metabolic endotoxemia [59], as characterized by increased circulating markers of inflammation, and hypothesized to be linked to transient bacteremia due to reduced gut barrier function [59]. Most importantly, ISO has been shown to have E2 mimetic effects in preventing ovariectomy-induced metabolic dysfunctions [60], adipose deposition [61], hypertriglyceridemia, and hepatic status [62] in animal studies. Isoflavones have been shown to improve intestinal barrier integrity [63] and reduce colitis in animal models [64] potentially through modulation of the gut microbiome [65]. ISO may therefore confer protection against diet-induced metabolic dysfunction and reduce the development of insulin resistance in males and post-menopausal women, therefore supporting our proposal that ISO could be an effective alternative to E2 treatment.

Although we claim to explain the metabolic sexual dimorphism by effects of E2 through gut microbiota, we acknowledge that many other studies in the field showed that the major impact of estrogens on the metabolism are through estrogen receptor expression in metabolic tissues [66–69]. Clinical trials revealed that hormone replacement therapy (HRT) in postmenopausal women reduced the features of MS and inflammation [70]. Conversely, findings from Women’s Health Initiative clinical trials (WHI-CT) [71] did not support use of HRT for chronic disease prevention [72]. However, the WHI-CT results were based on a group of women who were much older than those normally treated with HRT and who had other numerous risk factors [73]. Noticeably, there were very limited surveys performed addressing the effects on the metabolic syndrome components in postmenopausal women [74]. Early treatment with low-dosage HRT in healthy perimenopausal women was found to have beneficial effects on the components of metabolic syndrome and could decrease the risk of cardiovascular events [74] since the absolute risk of CVD events were markedly lower in younger, compared to older, women [71].

Although our results from FMT (Fig. 3) and antibiotics (Fig. 4) experiments contradict the protective effect of endogenous estrogen against obesity in female mice, it is well known that antibiotic usage [75] or gut dysbiosis [76] impacts estrogen metabolism mediated by microbiota Consequently, changes in circulating levels of estrogen (Additional file 1 Figure S3m and Additional file 1: Figure S4e) were found in female mice that received male microbiota transplants or antibiotic treatment. We hypothesize that estrogen-mediated gut microbiome changes may be the cause for sex differences in obesity and MS in this study. It is important to note the estrogen-mediated sex differences and the role of the microbiome have been linked in other disease conditions (e.g., autoimmunity) [77]. The bacterial changes reported here are similar to previous studies where ovariectomy or estrogen supplementation was performed. A study with OVX animals showed elevated *Firmicutes* to *Bacteroidetes* ratio [78] and *Escherichia coli* [79] compared to normal females. Likewise, it was shown that E2 supplementation elevated the relative abundance of *Akkermansia* and *Bifidobacterium* in the male and OVX mice respectively [80–82]. Estrogen inhibited the overgrowth of *Proteobacteria* and *E. coli* and decreased the levels of LBS and LBP under simulated microgravity [83]. In addition, E2 supplementation in male [84] or OVX mice [61] prevented obesity and MS, and genistein prevented obesity and metabolic dysfunction in the mice, which was similar to E2 supplementation in the same study [60, 61].

## Conclusions

Our novel data demonstrate that the gut microbiome mediates sexual dimorphism in MS. Overall measures of correlation, pairwise correlations, and multivariate correlation analyses between the microbiota and host parameters that we performed provided novel insight into the host–microbiota system in the context of sexual dimorphism in WD-induced MS. Estrogen or estrogen-like compounds induced elevated IAP levels likely by upregulating the function of the KLF4 transcription factor that targets IAP and subsequent gut microbiome changes lower LPS production and gut permeability, resulting in reduced ME and systemic LGCI with subsequent reduction in the susceptibility to develop WD-induced MS in estrogen-treated males and post-menopausal women. Because exogenous estrogen administration to male causes deleterious effects (e.g., feminization and cardiac dysfunction) [85], compounds with estrogen-like activity (e.g., isoflavones and 17α-estradiol [85]) and non-feminizing effects may represent an alternative approach to the management of obesity and MS in males. Understanding the molecular basis of estrogen-mediated changes in IAP activity and gut microbiome may provide new approaches to the management of obesity-associated metabolic disease in men and menopausal women with estrogen deficiency, a condition that can last approximately 30 years of a woman’s life [86].

## Methods

### Animals and diets

All the mice used in this study were wild-type (WT) on a C57BL/6 background and bred at the Massachusetts General Hospital (MGH) animal facility or purchased from Charles River Laboratories. Mice were housed in a biosafety level 2 room in hard top cages with two or three mice per cage. Mice were maintained in a temperature-controlled room (22–24 °C) with a 12-h light/12-h dark diurnal cycle and allowed for food and water ad libitum. Diets used in this study were either normal chow diet (CD) (Laboratory Rodent Diet 5001) from LabDiet or western diet (WD) (D12079B) from Research diets, Inc., NJ, USA. All animal procedures in this study were carried out in accordance with the guidelines approved by the MGH Subcommittee on Research Animal Care.

### Animal experiments

Body weight and food intake were measured every week for all mice. All food was replaced weekly to avoid contamination. For collection of blood samples, mice were fasted for 6 h during the light phase period and blood was taken from the facial vein unless otherwise specified. After 6 h fasting, animals used in this study were euthanized by i.p. injection of pentobarbital (200 mg/kg). Several experiments with special treatments are described below.

1. *Determination of sexual dimorphism in metabolic endotoxemia, low-grade chronic inflammation, and metabolic syndrome*: Male (M) WT (*n* = 11) and female (F) (*n* = 11) mice were weaned and switched to western diet (WD) until the age of 20 weeks to induce severe WD-induced obesity and MS. Both groups were subjected to analysis of markers of metabolic endotoxemia (ME) (including LPS, LBP, sCD14, and intestinal permeability), systemic low-grade chronic inflammation (LGCI) (including TNF-α, IL-1β, IL-6, MCP-1, and IL-10), and metabolic syndrome (MS) (including weight gain, glucose tolerance test with area under the curve, insulin resistance index assessed by HOMA-IR, serum lipid profile including total cholesterol (TC), triglyceride (TG), low-density lipoprotein-C (LDL-C) and high-density lipoprotein-C (HDL-C) and atherogenic index, non-alcoholic fatty liver score and serum aspartate transaminase (AST) and alanine transaminase (ALT), and analysis of fecal microbiota by 16S rRNA gene sequencing and serum LPS levels by LAL assay. The mice were then euthanized and white adipose tissue (WAT), which includes visceral (vWAT), subcutaneous (sWAT), and epididymal (eWAT) fat pads, and liver weights were taken. A portion of the liver was stored in 10% formalin for histological analysis. Tissues were snap frozen in liquid nitrogen and then stored at − 80 °C for future analyses.
2. *Determination of 17β-estradiol effects on gut microbiome, metabolic endotoxemia, low-grade chronic inflammation, and metabolic syndrome*: Ten-week-old male (M), female (F), and ovariectomized (OVX) mice maintained on control diet were purchased from Charles River Laboratories and divided in to five groups (*n* = 5/group), and were fed WD and 17β-estradiol (E2) from week 11 to week 17. The five groups are (1) M, (2) F, (3) M+E2, (4) OVX, and (5) OVX+E2. E2 (Sigma, USA) was prepared and given to groups 3 and 5 in the drinking water as described previously [87]. E2 was dissolved in 95% ethanol (5 mg/mL). Solubilized E2 was added to the drinking water to produce concentration of 4000 ng E2/mL water with final ethanol concentration of 0.1%. Groups 1, 2, and 4 received a normal drinking water bottle with 0.1% ethanol. The water bottles were changed every week for all the groups. After 6 weeks, mice were subjected to analysis for markers of ME and systemic LGCI and MS as mentioned in section 1. The mice were then euthanized and WAT and liver weights were taken. A portion of the liver and duodenum was stored in 10% formalin for histological analysis. Tissues were snap frozen in liquid nitrogen and then stored at − 80 °C for future analyses.
3. *Fecal microbiota transplantation (FMT)*: Mice from each group were individually housed for receiving antibiotic treatment to the end of the experiment. Fecal microbiota in place of cecal microbiota was transplanted for convenience and in order to minimize the number of animals used as donors. FMT with fecal content from donor male (M) mice that were fed WD for 10 weeks was performed on female 10-week-old mice [19]. Before the microbial transplantation, recipient mice (M → F) were treated with a 200 μL antibiotic cocktail (ampicillin, 1 g/l; metronidazole, 1 g/l; vancomycin, 0.5 g/l; neomycin, 0.5 g/l) (Sigma, USA) administrated by oral gavage once a day for 3 days. During the last 8 h of antibiotic treatment, mice were fed a WD to facilitate subsequent colonization. Fresh fecal pellets from donors were immediately weighed and placed into Ringer’s solution and then diluted to 10 mg/mL. Immediately after diluting the fecal materials, fecal solutions were gavaged (200 μL per mouse) to 4-h-fasted female recipients. Control groups of mice (males and females) were force-fed with 200 μL of transfer buffer alone to eliminate the effects of gavage per se. Two days later, these mice received another gavage to exclude possibility of any unsuccessful inoculation. We conducted this FMT procedure once a week for the next 3 weeks. After the first microbiota gavage, all mice were fed a WD for 20 weeks. Measurement of body composition (fat mass) was performed using nuclear magnetic resonance (NMR) technique (minispec Body Composition Analyzer based on Time Domain NMR) that provides noninvasive and rapid measurement without anesthetics. Fecal samples from these three groups (M/F/M → F; *n* = 5 per group) were collected after WD feeding for 20 weeks and three fecal samples from each group were subjected to 16S rRNA sequencing.
4. *Antibiotic (ABX) treatment*: 10-week-old male (*n* = 5) and female (*n* = 5) mice maintained on chow diet were switched to a WD until the age of 20 weeks to induce sexual dimorphism in MS. After collecting serum and stool for baseline measurements, both males and females receiving a WD started receiving a broad spectrum antibiotic cocktail (ABX) containing ampicillin (1 g/l), vancomycin (500 mg/l), neomycin sulfate (1 g/l) (added to the drinking water), and metronidazole (100 mg/kg) (orally gavaged every 12 h) for 6 weeks to deplete the gut microbiota [88]. Validation of successful depletion of gut microbiota after the antibiotic treatment was performed as described previously [88]. Briefly, bacterial cultivation (both aerobic and anaerobic bacteria) of feces was performed on day 24 of the antibiotic treatment mice as described previously [14]. The detection limit of the assay (successful depletion) was defined as 1 cfu/mg feces. In addition, the bacterial genomic DNA was extracted from fresh stool samples of these mice and 16S rRNA gene copies for all bacteria (Table S4) was measured with qPCR method as described below. Both bacterial cultivation (cfu/mg feces) and qPCR results (Ct values) were compared with results from pre-antibiotic treatment fecal samples to confirm a significant depletion of gut microbiota. Mice with antibiotic treatment were subjected to analysis for markers of ME and systemic LGCI and MS as mentioned in section 1.
5. *Analysis of the effects of isoflavones (ISO) on gut microbiome, ME, LGCI, and MS*: Ten-week-old male (M) mice were fed a WD for 4 months to induce MS and then divided into two groups: group 1: M, (*n* = 4), and group 2: M+ISO (*n* = 6). ISO such as genistein and daidzein (Cayman, USA) were supplemented at 0.1% in the WD, the M group was fed a WD, and the M+ISO group was fed a WD supplemented with ISO for the next 5 weeks. Mice were then subjected to analysis for markers of ME and systemic LGCI and MS as mentioned in section 1. The mice were then euthanized and white adipose tissue (WAT) and liver weights were taken. A portion of the liver and duodenum was stored in 10% formalin for histological analysis. Tissues were snap frozen in liquid nitrogen and then stored at − 80 °C for future analyses.
6. *Analysis of the effects of intestinal alkaline phosphatase (IAP) inhibition on gut microbiome, ME, LGCI, and metabolic abnormalities*: To determine the effects of inhibiting endogenous IAP activity, 12-week-old WT mice on a WD were divided into three groups ((male (M), female (F), and F+L-phe)) (*n* = 5 per group) and allowed to drink autoclaved water alone or water containing 10 mM L-phenylalanine (L-phe) (Sigma, USA). After 8 weeks, the serum and fecal samples were collected to analyze the markers of ME, LGCI, and MS.

### Extraction of genomic DNA and profiling of the 16S rRNA gene by next generation

#### Sequencing

##### Fecal DNA extraction and 16S rRNA gene sequencing

Bacterial genomic DNA was extracted from fresh stool samples (100–180 mg) using the QIAamp DNA Stool Mini Kit (Qiagen, Valencia, CA), following the manufacturer’s instructions. To increase effectiveness, the lysis temperature was increased to 95 °C. Eluted DNA was treated with RNase and analyzed using a Nanodrop spectrophotometer (Biotek, Winooski, VT). Sample concentration and purity was determined by absorbance at 260 nm and the A260/A280 ratio, respectively. DNA samples packed with dry ice were shipped to APC Microbiome Institute (University College Cork, Cork, Ireland), and samples were sequenced as previously mentioned [37]. Briefly, V3–V4 amplicons for Illumina sequencing were generated according to the 16S metagenomic sequencing library protocol (Illumina). An initial PCR reaction utilized primers specific for amplification of the V3–V4 region of the 16S rRNA gene, (Forward primer 5′TCGTCGGCAGCGTCAGATGTGTATAAGAGACAGCCTACGGGNGGCWGCAG; reverse primer 5′ GTCTCGTGGGCTCGGAGATGTGTATAAGAGACAGGACTACHVGGGTATCTAATCC). PCR product clean-up and purification was achieved using the Agencourt AMPure XP system (Labplan, Dublin, Ireland). A second PCR incorporated a unique indexing primer pair for each sample (Illumina Nextera XT indexing primers, Illumina, Sweden). The products were again purified using the Agencourt AMPure XP system. Samples were quantified using the Qubit broad range DNA quantification assay kit (Bio-Sciences, Dublin, Ireland). Following quantification, samples were pooled in equimolar amounts (4 nM) and sequenced at Clinical Microbiomcs, Copenhagen, Denmark, using Illumina MiSeq 2 × 300 bp paired end sequencing.

##### a)Bioinformatics

Three hundred-base pair paired-end reads were assembled using FLASH with parameters of a minimum overlap of 20 bp and a maximum overlap of 120 bp [89]. The QIIME suite of tools, v1.8.0, was used for further processing of paired-end reads, including quality filtering based on a quality score of > 25 and removal of mismatched barcodes and sequences below length thresholds [90]. Denoising, chimera detection, and operational taxonomic unit (OTU) grouping were performed in QIIME using USEARCH v7 [91]. Taxonomic ranks were assigned by alignment of OTUs using PyNAST to the SILVA SSURef database release 111 [92]. Generation of α and β diversities and analysis and visualization of principal coordinate analysis (PCoA) plots were performed using PAST and XLSTAT software. The α-diversity of each group was calculated based on the annotated data using the diversity indices of the PAST version 2.17 software program [93]. Based on a non-parametric two-sample *t*-test using the default number of Monte Carlo permutations (999), comparative analyses of the group-specific α-diversity indices were performed. Ordinations are the dimensional-reduction techniques which are commonly used to visualize complex relationships between communities between groups (β-diversity). Dimensional reduction of the Bray-Curtis distance between microbiome samples using PCoA ordination method (PAST software) was done and significant differences among groups were tested with permutational multivariate analysis of variance (PERMANOVA), a multivariate non-parametric one-way ANOVA, which utilizes the sample-to-sample Bray-Curtis distance matrix directly. Taxa which were primarily responsible for an observed difference between groups were identified by SIMPER (similarity percentage analysis) method and their contribution to groups (between and within groups) were analyzed using the PCA variance-covariance type ordination (PAST software) method. Differential abundance analysis (non-parametric ANOVA with Benjamini-Hochberg FDR-corrected *P* values < 0.05) was performed on the RA of microbiota data at different levels of taxonomy to identify taxa with FDR-corrected *P* values < 0.05 (XLSTAT software; Addinsoft, USA) [94] and then their RA (normalized to percentage) were shown by a heat map with hierarchal clustering (HCN) analysis [95] using GraphPad Prism version 7.01 (La Jolla, CA). Linear discriminant analysis (LDA) effect size (LEfSe) is a biomarker discovery and explanation tool for high-dimensional data. It couples statistical significance with biological consistency and effect size estimation [96]. Microbiota-based biomarker discoveries were done with LEfSe using the online galaxy server (https://huttenhower.sph.harvard.edu/galaxy/), and the LDA scores derived from LEfSe analysis [96] were used to show the relationship between taxon using a cladogram (circular hierarchical tree) of significantly increased or decreased bacterial taxa in the gut microbiota between groups. Levels of the cladogram represent, from the inner to outer rings, phylum, class, order, family, and genus. Color codes indicate the groups, and letters indicate the taxa that contribute to the uniqueness of the corresponding groups at an LDA of > 2.0. Unweighted pair-group method with arithmetic means (UPGMA) hierarchical clustering analysis diagram based on Bray-Curtis distance matrix was obtained using PAST version 3.11. Class trees were used to demonstrate similarity between samples, the clustering tree branch length was a measure of the cluster effect.

##### b)Putative metagenome identification

Microbial functions were predicted using 16S ribosomal RNA sequencing and phylogenetic reconstruction of unobserved states (PICRUSt) software (version 1.0.0) as described [29]. The predicted genes and functions were aligned to the KEGG database (version 66.1, May 1, 2013). PCA and PERMANOVA statistics were applied to check whether the groups were clustered according to predicted gene enrichments for microbial functions. LEfSe analysis [96] was utilized to determine significant putative KEGG orthologs and pathway analyses [96].

### Lipopolysaccharide (LPS) concentration

Serum LPS concentrations were measured with a Toxin Sensor Chromogenic Limulus Amebocyte Lysate (LAL) Endotoxin Assay Kit (GenScript, Piscataway, NJ), following the manufacturer’s instructions [15]. Briefly, serum samples were diluted 10- to 50-fold with endotoxin-free water, adjusted to the recommended pH, and heated for 10 min at 70 °C to minimize inhibition or enhancement by contaminating proteins. LAL reagents were added to serum and incubated at 37 °C for 45 min, and the absorbance was read at 545 nm. All samples were validated for recovery and internal coefficient variation using known amounts of LPS.

### Intestinal permeability

Intestinal permeability was determined as previously described [15]. Briefly, mice were gavaged with phosphate buffered saline (PBS, pH 7.2) containing 600 mg/kg body weight FITC-dextran (40 kDa, Sigma-Aldrich, USA). Blood samples (120 μL) were collected after 90 min. Serum was diluted with an equal volume of PBS, and fluorescence intensity was measured using a fluorospectrophotometer (excitation wavelength 480 nm and emission wavelength 520 nm; Perkin-Elmer, Waltham, MA). Serum FITC-dextran concentrations were calculated from a standard curve of serially diluted FITC-dextran in PBS.

### Measurement of intestinal alkaline phosphatase (IAP) level and activity

Small intestinal IAP specific activity (as it relates to protein) was measured as previously described [15] and expressed as picomoles pNPP hydrolyzed/min/μg of protein. Briefly, thoroughly washed duodenal tissues were homogenized with lysis buffer (150 mM NaCl, 10 mM Tris·HCl, pH 7.5, 1% sodium deoxycholate, 1% Nonidet P-40, 10 mM EDTA, 0.1% SDS, including protease inhibitor mixture; Sigma) followed by incubation on ice for 30 min. Thereafter, the homogenates were centrifuged twice at 4 °C at 15,000*g* for 15 min, and the supernatants were collected to determine IAP activity as well as protein concentration. The Coomassie Blue Protein Assay (Bradford) kit from Fisher Scientific was used for protein quantification. For IAP assay, 25 μL of supernatant was mixed with 175 μL phosphatase assay reagent containing 5 mM of p-nitrophenyl phosphate (pNPP) followed by determining optical density at 405 nm. The specific activity of the enzyme was expressed as picomoles pNPP hydrolyzed/min/μg of protein. Protein concentration in a specific sample was determined using the protein assay reagents from Fisher Scientific.

### Cell culture experiments

The human colon carcinoma cell line (Caco-2) was obtained from American Type Culture Collection (ATCC) (Rockville, MD) and cells were cultured in Dulbecco’s modified Eagle’s medium (DMEM) (Corning Inc., NY, USA) containing 4.5 g/l glucose, 4 mmol/l l-glutamine, and 1 mmol/l sodium pyruvate, and supplemented with 10% fetal bovine serum (Cell Applications, Inc., San Diego, CA), 100 U/mL penicillin, and 100 μg/mL streptomycin (Gibco, NY, USA) in a humidified atmosphere of 5% CO_2_ at 37 °C. They were routinely subcultured when they were about 80% confluent. The culture medium was changed every other day. Cells were always > 90% viable, as shown by trypan blue (Invitrogen, Carlsbad, CA, USA) exclusion. Cells were passaged every 3–4 days by treatment with 0.1% trypsin (Gibco) and 0.04% ethylenediaminetetraacetic acid (EDTA) and then plated at a density of 1.3–2 × 10^4^ cells/cm^2^. Cells at passage number 17 were used for the experiments. All assays were done using only differentiated Caco-2. Cells were seeded in to six-well plates at 2 × 10^4^ cells per well and treated with vehicle (ethanol or DMSO) or 10 nM 17β-estradiol (E2) for 24, 48, and 72 h or each 25 μM genistein (G) or daidzein (D) or G+D mixture for 72 h. In a subset of experiments, Caco-2 cells were pre-treated with L-phenylalanine (10 mM) for 24 h and then they were treated with either vehicle or each 25 μM G+D mixture or 10 nM E2 for 72 h. Medium was removed and cells were washed twice with ice-cold PBS, scraped, lysed in Trizol (Invitrogen), and stored at − 80 °C until mRNA was isolated or homogenized with 200 μL radioimmunoprecipitation assay (RIPA) buffer (50 mM Tris-HCl, pH 7.4, 150 mM NaCl, 1% Triton X-100, 1% sodium deoxycholate, 0.1% sodium dodecyl sulfate) containing protease inhibitor cocktail (Sigma), incubated on ice for 30 min, centrifuged at 14,000*g* for 10 min at 4 °C and the supernatant was collected and stored at − 80 °C for western blotting analysis.

### Western blotting analysis of IAP

Western blotting on tissues and Caco-2 cell lysates was performed as previously described [14]. After thawed, the protein samples derived from Caco-2 cells, the homogenates were centrifuged at 15,000*g* at 4 °C for 15 min, and the supernatants were collected. The duodenum section of small intestinal tissues was harvested and cut open longitudinally and luminal contents were removed. The tissues were washed with PBS and homogenized with liquid nitrogen, and homogenates were mixed with RIPA buffer, incubated on ice for 30 min, and centrifuged at 14,000*g* for 10 min at 4 °C, and the supernatant was collected. Protein concentration of Caco-2 and tissue homogenates was quantified by the Coomassie blue protein assay (Thermo Scientific, Rockford, IL, USA) using bovine serum albumin (BSA) as the standard. Proteins (30 μg) were resolved on SDS-PAGE gels and transferred onto nitrocellulose membranes (Osmonics, Minnetonka, MN, USA). The membranes were blocked with 5% nonfat dry milk in Tris-buffered saline with 0.05% Tween 20 (TBS-T) for 1 h at room temperature and then probed with IAP primary antibodies (GTX27322, GeneTex, San Antonio, TX, USA) in 5% non-fat dry milk in TBS-T at 4 °C overnight. After washing three times in TBS-T, the blots were further incubated with the corresponding secondary antibodies conjugated with horseradish peroxidase for 1 h at room temperature (Santa Cruz Biotechnology, Santa Cruz, CA). Chemiluminescence was detected with Pierce ECL western blotting substrate (Thermo Scientific, Rockford, IL, USA) and visualized by ChemiDoc MP Imaging System (Bio-Rad, Hercules, CA, USA).

### Immunohistochemical analysis of IAP

Formalin-fixed duodenal tissues and IAP primary antibodies (GTX27322, GeneTex) [14] were given to MGH core (Boston, MA). Prepared IHC slides were analyzed under light microscope and images of IAP staining and localization was taken by using × 20 magnifications. All pictures were taken with the same exposure conditions without autoscaling.

### RV coefficient

The RV coefficient was calculated between the microbial genera (FDR-corrected *P* value < 0.05) and the host parameters (markers of ME, LGCI and MS). The RV coefficient is a multivariate generalization of the Pearson correlation coefficient [97].

### Correlation network analysis

Network-based analytical approaches have the potential to help disentangle complex host-microbe interactions [98]. Pairwise correlations between each microbiota (genera that are present at < 0.1% relative abundance in > 75% samples have been removed to avoid detecting spurious correlations among low-abundance OTUs) and host parameter (markers of ME, LGCI, and MS) were calculated using Spearman’s nonparametric rank correlation coefficient [99]. Using those significant (*P* < 0.05) correlation coefficients, a correlation network (Fruchterman Reingold and label adjust layout) was built where nodes represent either a microbiota or a host parameter. For each microbiota and a host parameter, an undirected edge was added between the corresponding nodes in the correlation network. Edges (light black links indicate positive and blue links indicate negative associations) represent statistically significant correlations (*P* < 0.05). Correlations were calculated using the PAST software version 2.17 and the network was visualized in Gephi Graph Visualization and Manipulation software version 0.9.2 [100]. Nodes were colored based on “data type” and sized based on “betweeness centrality (BC).” BC is a network centrality measure that quantifies the influence of a node in connecting other nodes in a network. It represents the fraction of all shortest paths in the network that pass through a given node. The nodes with the highest BC are usually known as highly central or hubs. A “module or component” in the network is a set of nodes connected to each other by many links, while connected by few links to nodes of other groups, so modules are elementary units of any biological network, and their identification and characterization provides us with more information about the local interaction patterns in the network and their contribution to the overall structure, connectivity, and function of the network. Modules are biologically important when considered as isolated, taxonomic, evolutionary, or functional modules. High modularity indicates that the network has dense connections within certain groups of nodes and sparse connections between these groups.

### Multivariate statistical analysis

Partial least square regression (PLS-R) was used to associate the microbial composition to host parameters including jackknife-based variable selection [98]. PLS-R is recommended in cases of regression where the number of explanatory variables is high, and where it is likely that the explanatory variables are correlated. Leave one-out cross-validation (LOO-CV) was applied. The Q^2^ cumulated index (Q2_cum_) measures the global goodness of fit and the predictive quality of the models. Q2_cum_ is also used to test the validity of the model against over-fitting. The cumulated R^2^Y and R^2^X cum that corresponds to the correlations between the explanatory (X) and dependent (Y) variables with the components are very close to one with two components in all the models. This indicates that the two components generated by the PLR-R summarize well both the Xs and the Ys. The results are also presented in PLS scatter plots for subject clustering and variables. The R^2^ (coefficient of determination) indicates the % of variability of the dependent variable (Y) which is explained by the explanatory variables (X). Parameters (variable importance in the projection values 1 or > 1.0) contributing to the multivariate PLS models were compared with the corresponding identified modules (Fig. 7b–d) in the correlation networks. All analyses were performed using precise algorithm in the XLSTAT software version 2017.6.

### Statistical analysis

Data was shown as mean ± standard error of mean (SEM). Box-plots (box showing the median, and the 25th and 75th percentiles, and the whiskers of the graph show the largest and smallest values) were also used to express the data. Unpaired Student’s *t* test was performed for experiments having only two groups. Either ordinary or repeated measures one-way or two-way analysis of variance (ANOVA) with Tukey’s or Sidak’s multiple comparisons post-test were used for experiments having more than two groups. If unequal variance was detected, data were analyzed using non-parametric tests. Differences were considered significant at *P* < 0.05. Statistical analyses, including heat-map preparation, were performed using GraphPad Prism version 7.01 (GraphPad Software, La Jolla, CA). Differential expression analysis on 16S sequencing data was conducted using XLSTAT software program [94]. Multivariate statistical analyses and power analyses (alpha = 0.05; effect size = 0.8) were conducted using PAST (version 2.17) [93] and XLSTAT (version 2017.6) software products.

## Additional file

Additional file 1: **Figure S1.** Sexual dimorphism in the MS and ME and LGCI. **Figure S2.** Hierarchical clustering and predicted functional analysis. **Figure S3.** α-and β diversity analysis of fecal microbiota profile. **Figure S4.** Markers of low-grade inflammation and metabolic syndrome. **Figure S5.** Diversity analysis and antimicrobial peptides mRNA expression. **Figure S6.** The ingredients of Western diet. **Table S1.** Pairwise comparison of study groups with permutational multivariate analysis of variance. **Table S2.** Differential abundance analysis of fecal microbiota profile. **Table S3.** Pairwise differential abundance analysis between groups. **Table S4.** Primer sets used for real-time quantitative PCR. (DOC 13997 kb)
