# Supplementary material for: Estrogen-mediated gut microbiome alterations influence sexual dimorphism in metabolic syndrome in mice
Source: Microbiome. 2018 Nov 13;6:205. doi: 10.1186/s40168-018-0587-0 (PMC6234624; doi:10.1186/s40168-018-0587-0)
Supplement: Supplementary file 1 — Figure S1. Sexual dimorphism in the MS and ME and LGCI. Figure S2. Hierarchical clustering and predicted functional analysis. Figure S3. α-and β diversity analysis of fecal microbiota profile. Figure S4. Markers of low-grade inflammation and metabolic syndrome. Figure S5. Diversity analysis and antimicrobial peptides mRNA expression. Figure S6. The ingredients of Western diet. Table S1. Pairwise comparison of study groups with permutational multivariate analysis of variance. Table S2. Differential abundance analysis of fecal microbiota profile. Table S3. Pairwise differential abundance analysis between groups. Table S4. Primer sets used for real-time quantitative PCR. (DOC 13997 kb) [file 40168_2018_587_MOESM1_ESM.doc]

**Supplementary Information**

**Supplementary methods**

**Plasma lipid profiling and liver function testing.** Serum total cholesterol (TC) and triglycerides (TG) were measured by the Clinical Pathology Laboratory at the MGH Center for Comparative Medicine. Serum high-density lipoprotein cholesterol (HDL-C) was enzymatically determined using a kit from Pointe Scientific (Canton, MI) following the manufacturer’s instructions. The concentration of low-density lipoprotein cholesterol (LDL-C) was calculated using the Friedewald equation: LDL cholesterol = total cholesterol – HDL cholesterol–TG/5, and the atherogenic index (TC-HDL-C/HDL-C) were calculated as described previously . Serum alanine aminotransferase (ALT) and aspartate aminotransferase (AST) were measured using a kit from B100 Scientific (Austin, TX), following the manufacturer’s instructions.

**Glucose tolerance test (GTT).** GTT was performed in nonanesthetized mice as described previously. Briefly, mice were fasted for 6 h in the day time, fasting blood sugar was measured, and glucose [1.0 g/kg body weight, 20% (wt/vol) glucose solution] was administered by i.p. injection. Small blood samples (microliters) were drawn from the tip of the incised tail at 15, 30, 60, 90, and 120 min to measure blood glucose levels. Twenty microliters of blood was collected to assess serum insulin concentration. Glucose tolerance was assessed by calculating the incremental area under the curve (AUC) of each GTT. The homeostasis model assessment of insulin resistance [HOMA-IR =glucose (mg/dL) × insulin (mU/L)/405] was calculated as an insulin sensitivity index .

**Hepatic triglyceride measurement.** Total hepatic triglycerides (TG) were measured as previously described previously . Briefly, frozen tissues (~100 mg) were homogenized in a 1-mL solution containing 5% NP‐40 (Sigma Aldrich, St. Louis, MO) in water. Samples were heated to 80‐100°C for 2‐5 min until the NP‐40 solution became cloudy. Samples were cooled to room temperature and heating was repeated to solubilize all TGs. Samples were centrifuged for 2 min at top speed to remove insoluble material. Supernatants were diluted 10-fold with deionized water. TG content was determined using a triglyceride quantification kit (BioVision, Mountain View, CA) following the manufacturer’s protocol.

**Liver histopathology and hepatic steatosis scoring.** Formalin fixed liver samples were stained with hematoxylin and eosin or Masson's trichrome stain (MGH Core, Boston, MA) and examined by an independent experienced pathologist (Dr. Bhan AK) blinded to group assignment. Hepatic steatosis was graded based on the number and size of stained fat droplets: 0 (none); 1+ (<5%); 2+ (6-33%); 3+ (34-66%); 4+ (>66%); 5+ (>66% plus very large fat globules). Lobular inflammation was graded based on the number of foci: 0 (none); 1 (<2foci); 2 (2-4foci); 3 (>4foci); Individual score for fatty liver and inflammation was combined and expressed as liver histopathological injury score. Images were taken by using either 10x or 40x magnification.

**Measurement of cytokine levels and other circulating factors.** Serum levels of TNF-α, IL-1β, IL-6, MCP-1, and IL-10 were determined using Bio-Plex immunoassays formatted for magnetic beads (Bio-Rad), following the manufacturer’s instructions. Xponent software (Luminex, Austin, TX) was used to acquire and analyze data. Enzyme-linked immunosorbent assay (ELISA) kits were used to determine serum levels of 17β-estradiol (Cayman Chemical Company, Ann Arbor, MI,), Lipopolysaccaride binding Protein (LBP) (NeoBioLab, Cambridge, MA), soluble CD14 (sCD14) ((MyBioSource, San Diego, CA) and insulin (Crystal Chem, Downers Grove, IL), according to the manufacturers’ instructions. Blood glucose was measured using Contour blood glucose meter and strips (Bayer HealthCare, Montville, NJ).

**Gene expression analysis by semi-quantitative PCR.** Total RNA was isolated from Caco-2 cell lysates and tissue samples of small intestine (duodenum) (~50 mg) using TRIzol reagent (Invitrogen Life Technologies, Grand Island, NY), following the manufacturer’s instructions. RNA concentrations and purity were estimated by determining the A260/A280 ratio with a Nanodrop spectrophotometer (Biotek). Reverse transcription of mRNA was performed using the iScript cDNA Synthesis Kit (Bio-Rad). qPCR was carried out using SYBR Green in a PRISM 9000 Light Cycler (Applied Biosystems), following the manufacturer’s instructions. Primers (**Supplementary Table 4**) used in this study , were synthesized by Invitrogen, USA. All samples were processed in triplicate and normalized to β -actin. The expression levels were calculated using the Δ Δ Ct method after correcting for differences in PCR efficiencies, and values were expressed relative to those of the control group.

**Quantitative real-time PCR (qRT-PCR) assessment of 16S rRNA genes.** Absolute quantification of *phylum* *Firmicutes and Bacteroidetes****,*** *family Enterobacteriacea and genus Bifidobacterium****,*** relative quantification (RQ) of *Proteobacteria* *phylum* and *Akkermansia mucinophila* *species* were performed as previously described. Briefly, qRT-PCR was performed with a PRISM 9000 Light Cycler (Applied Biosystems, USA) using the iTaq universal SYBR Green Supermix (Bio-Rad, USA) and group-specific primers (**Supplementary Table 4**). Primer specificity and limits of detection were determined as previously described . Samples and standards were run in duplicate in total reaction volumes of 20 μl/well, containing 500 nM primers and 40 ng genomic DNA. Amplification and data acquisition was performed according to the protocol provided with the SYBR Green (Bio-Rad, Hercules, CA). Genomic DNA from reference strains; BEI Resources, Manassas, VA) were converted to 16S rRNA and serially diluted to generate standard curves. The 16S rRNA copy number was determined for each genomic standard as previously described . Ratios were calculated using 16S rRNA gene copy numbers per gram of stool. Data analysis was performed using MxPro QPCR software (v4.10, Agilent). For RQ, the Ct values from *Proteobacteria* and *A. mucinophila* were normalized to 16S to generate Δ-Ct values and then the Δ-Ct values were compared between groups. For example, A higher Δ-Ct value indicates lower abundance of the *A. mucinophila*. Melting-point curve analysis confirmed the specificity of the amplification products.

**Supplementay figures and figure legends**

Fig S 1

**Figure S1 Sexual dimorphism in the MS and ME and LGCI**. **.** In experiment one, male (M) and female (F) C57BL/6 mice (n=11/group) were fed Western diet (WD) for 20 weeks, and in experiment two, male, normal female and ovariectomized (OVX) female mice were divided in to 5groups (M/F/M+E2/OVX/OVX+E2) and were fed WD ± 17β-estradiol (E2) in the drinking water for 6 weeks (n=5/group). Sexual dimorphism in markers of metabolic syndrome (MS) (**A-N**), metabolic endotoxemia (ME) (**O-R**) and low-grade chronic inflammation (LGCI) (**S-W**) were studied. **(A)** Gross appearance of mice and intra-abdominal and gonadal fat distribution. (**B**) Body weight. (**C**) Subcutaneous (s), gonadal (g) and visceral (v) white adipose tissue (WAT) weight. (**D**) Energy intake. **(E)** Glucose intolerance curve (GTT), area under the curve (AUC) and Homeostatic model assessment of insulin resistance (HOMA-IR). (**F**) Hematoxylin and Eosin (H&E) stained liver specimens showing differences in the fatty liver and infiltration of inflammatory cells (aero head). (**G**) Liver histological score. Serum levels of ALT (**H**), AST (**I**), total cholesterol (TC) (**J**), triglycerides (TG) (**K**), LDL-C (**L**) and HDL-C (**M**). (**N**) Atherogenic index. Serum levels of lipopolysaccharides (LPS) (**O**), LPS-binding proteins (LBP) (**P**) and soluble CD14 (sCD14) (**Q**). (**R**) Serum levels of FITC-dextran macromolecules indicating intestinal permeability differences. Serum levels of cytokines such as TNF-α (**S**), IL-1β (**T**), MCP-1 (**U**), IL-6 (**V**) and IL-10 (**W**). (**X**) Energy intake. (**Y**) Atherogenic index. (**Z**) Serum AST levels. Data are expressed as mean ± SEM. Box-plots (box showing the median, and the 25th and 75th percentiles, and the whiskers of the graph show the largest and smallest values) were used for figures X,Y and Z. Data with different superscript letters are significantly different (*P* < 0.05). Student’s T-TEST or one way ANOVA followed by Tukey’s multiple comparisons test or two way ANOVA followed by Sidak's multiple comparisons test

Fig S 2


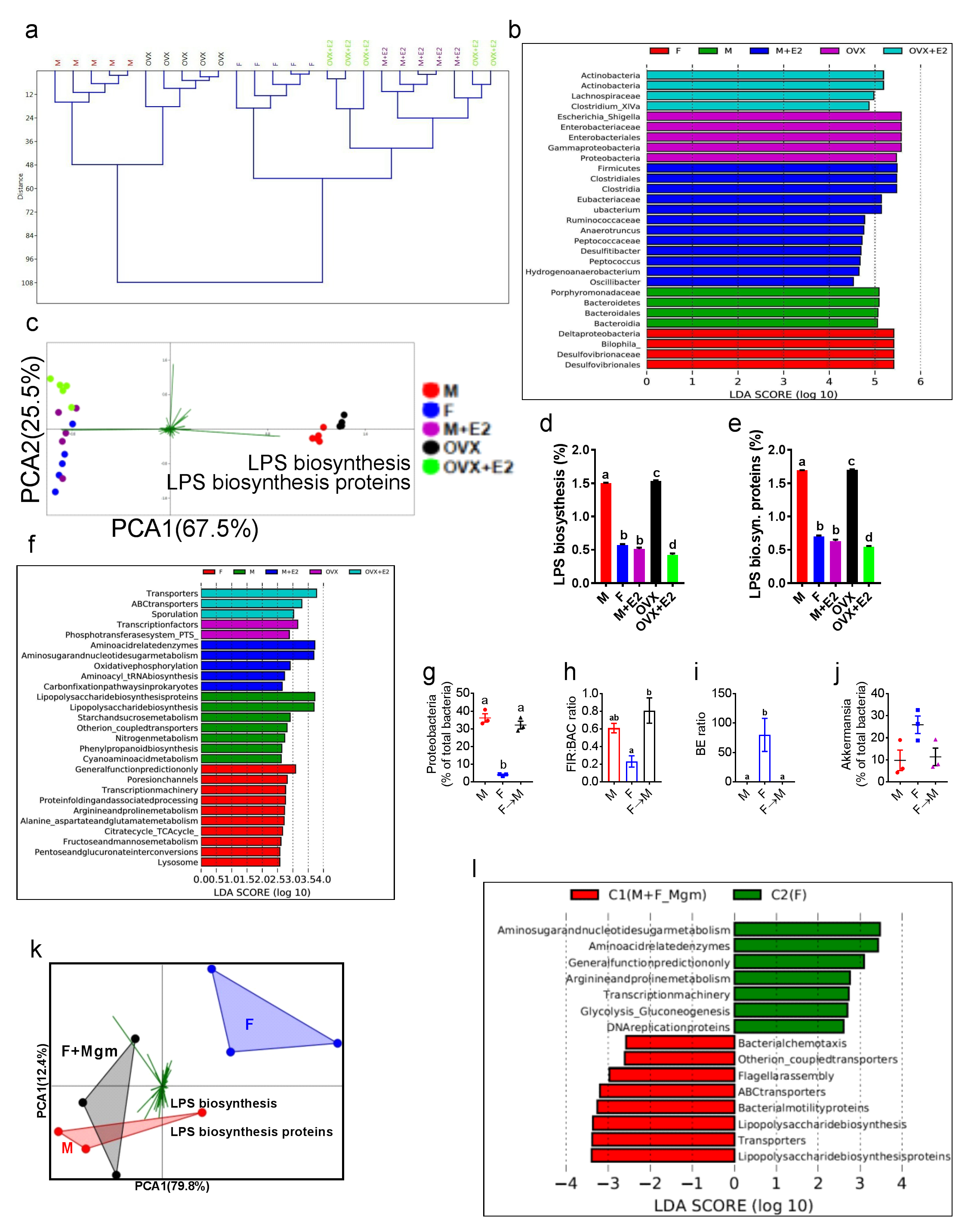


**Figure S2 Hierarchical clustering and predicted functional analysis.** Nonweighted group the average method (unweighted pair-group method with arithmetic means, UPGMA) clustering analysis diagram based on Bray-Curtis distance matrix for male (M), female (F), Ovariectomized female (OVX) and 17β-estradiol (E2) treated male (M+E2) and OVX female (OVX+E2) groups. Class tree is used to demonstrate similarity between samples, through the clustering tree branch length measure cluster effect. n=5/per group. (**b**) LDA scores derived from LEfSe analysis, showing the biomarker taxa (LDA score of >2 and a significance of *P* < 0.05 determined by the Wilcoxon signed-rank test). (**c**) RA of predicted microbial genes related to metabolism was identified using PICRUSt analysis. PCA (variance-covariance type) was then applied and the resulting scores were included as vectors. Among them, lipopolysaccharide (LPS) biosynthesis and related proteins were depicted as they expressed highest contribution to M and OVX groups. (**d-e**) RA of predicted bacterial genes involved in LPS biosynthesis and LPS biosynthesis proteins. (**g**) LDA scores derived from LEfSe analysis applied on RA of predicted microbial genes identified using PICRUSt analysis, showing the biomarker functions. (**g-j**) Scatter plots showing the RA of *Proteobacteria* (**g**), *Firmicutes* (FIR) to *Bacteroidetes* (BAC) ratio (**h**), *Bifidobacterium* (B) to *Enterobacteriacea* (E) ratio (**i**) and RA of genus *Akkermansia* (**j**). (**k**) RA of predicted microbial genes was identified using PICRUSt analysis. PCA (variance-covariance type) was then applied and the resulting scores were included as vectors. Among them, lipopolysaccharide (LPS) biosynthesis and related proteins were depicted as they expressed highest contribution to M and F+Mgm groups. (**l**) LDA scores derived from LEfSe analysis applied on RA of predicted microbial genes identified using PICRUSt analysis, showing the biomarker functions for clusters 1(M and F+Mgm) and 2 (F) identified with PCA analysis. Data with different superscript letters are significantly different (*P* < 0.05). Ordinary one way ANOVA followed by Tukey’s multiple comparisons test.

Fig S 3

**Figure S3 α-and β diversity analysis of fecal microbiota profile.** (**a**) Rarefaction curves for individual samples from male (M), female (F), Ovariectomized female (OVX) and 17β-estradiol (E2) treated male (M+E2) and OVX female (OVX+E2) groups (n=5). (**a**) Rarefaction curves (RFCs) with X axis representing number of sequences and Y axis representing number of observed taxa. (**b-e**) α-diversity (within group variations) measures ((Observed taxa **(b**), Chao-1 (**c**), Shannon (**d**) and Simpson (**e**)) for above mentioned groups. (**f**) Area under the curve (AUC) of glucose tolerance test conducted with male (M), female (F) and female with male fecal microbiota transplant (F+Mgm) groups (n=3). (**g**) LDA scores derived from LEfSe analysis, showing the biomarker taxa (LDA score of >2 and a significance of *P* < 0.05 determined by the Wilcoxon signed-rank test) for clusters 1(M and F+Mgm) and 2 (F) identified with PCA analysis. α-diversity measures (**h-k**), RFCs (**l**) and serum E2 levels (**m**) of M/F/F+Mgm groups. Data shown as mean ± SEM. Box-plots (box showing the median, and the 25th and 75th percentiles, and the whiskers of the graph show the largest and smallest values) were also used. Data with different superscript letters are significantly different (*P* < 0.05). Non-parametric two-sample *t*-test using Monte Carlo permutations (999) or one way or two way ANOVA followed by Tukey’s multiple comparisons test.

**Fig S4**

**
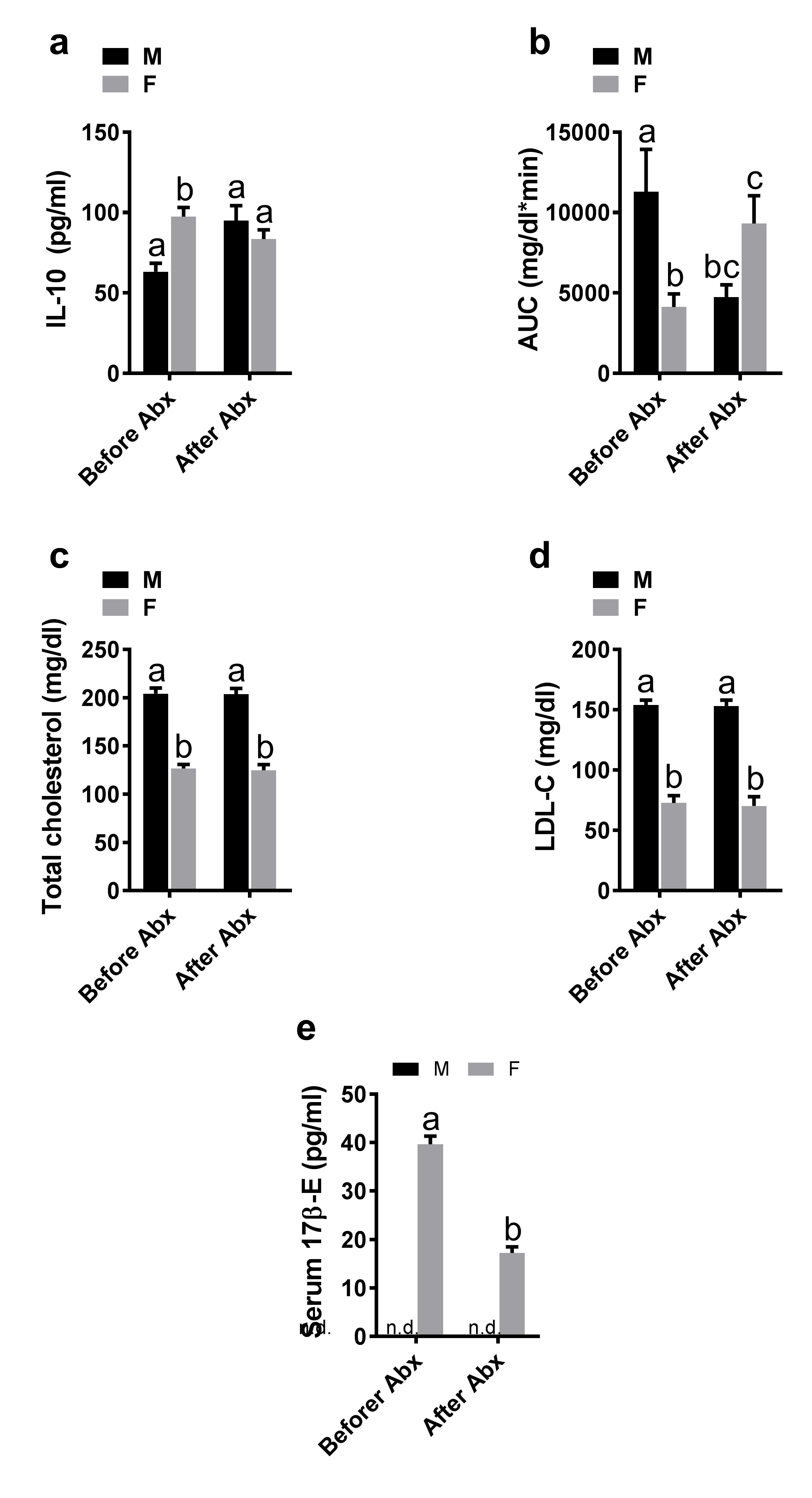
**

**Figure S4 Markers of low-grade inflammation and metabolic syndrome.** (**a**) Serum Interleukin 10 (IL-10) levels. (**b**) Glucose tolerance test area under the curve (AUC). (**c**) Serum total cholesterol. (**d**) Serum LDL-C. Data showed as mean ± SEM. (**e**) Serum E2 levels. Data with different superscript letters are significantly different (*P* < 0.05). Repeated measures two way ANOVA followed by Tukey’s multiple comparisons test. M, male; F, female; Abx, Antibiotics; n.d., not detectable.

Fig S 4

**Figure S5 Diversity analysis and antimicrobial peptides mRNA expression.** (**a**) Energy intake of Western diet fed male (M) (n=4) and male mice (M+ISO) received isoflavones (ISO) (n=6) with WD. (**b-c**) mRNA levels of estrogen receptor (ER)-α (**b**) and ER-β (**c**) in the duodenal tissues. α-diversity measures (**d-g**) and rarefaction curves (**h**) for M and M+ISO groups. **(i-j**) Messenger RNA expression of anti-microbial peptides (Reg3γ-, mBD-2, α-defensin, Ang4 and BPI) in the duodenal tissues of male (M) and M + Isoflavones groups and male (M), female (F), Ovariectomized female (OVX) and 17β-estradiol (E2) treated male (M+E2) and OVX female (OVX+E2) groups (n=5). (**j-k**) Duodenal tissue mRNA levels of KLF4. (**l-m**) Duodenal tissue mRNA levels of CDX1. Data shown as mean ± SEM. Data with different superscript letters are significantly different (*P* < 0.05). Student’s T-TEST or non-parametric two-sample *t*-test with Monte Carlo permutations (999) or One way ANOVA followed by Tukey’s multiple comparisons test.

**Fig. S6. The ingredients of Western diet.**

**Supplementary Tables with Legends**

**Table S1. Pair wise comparison of study groups with permutational multivariate analysis of variance.**

PERMANOVA, permutational multivariate analysis of variance; M, male; F, female; E2, 17β-estradiol; OVX, ovariectomized; n=5/ group. PERMANOVA with Bray-Curtis distance matrix was applied on the relative abundance. P<0.05 is considered significant.

**Table S2. Differential abundance analysis of fecal microbiota profile**.

| **Taxonomy** | **Features** | **p-value** | **FDR<0.05** | **M** | **F** | **M+E2** | **OVX** | **OVX+E2** |
| --- | --- | --- | --- | --- | --- | --- | --- | --- |
| *Phylum* | Lentisphaerae | 0.001 | Yes | 0.075 | 0.000 | 0.000 | 0.000 | 0.000 |
| *Phylum* | Actinobacteria | 0.001 | Yes | 0.013 | 0.387 | 0.009 | 0.000 | 2.236 |
| *Phylum* | Proteobacteria | 0.001 | Yes | 22.284 | 10.937 | 0.491 | 34.812 | 0.431 |
| *Phylum* | Firmicutes | 0.002 | Yes | 22.035 | 11.793 | 28.058 | 23.793 | 36.633 |
| *Phylum* | Candidatus_Saccharibacteria | 0.002 | Yes | 0.000 | 0.000 | 0.264 | 0.021 | 0.035 |
| *Phylum* | Deferribacteres | 0.008 | Yes | 5.958 | 5.464 | 8.644 | 3.566 | 8.153 |
| *Phylum* | Bacteroidetes | 0.010 | Yes | 35.843 | 40.795 | 37.322 | 15.448 | 28.124 |
| *Phylum* | Verrucomicrobia | 0.043 | Yes | 11.953 | 31.198 | 25.639 | 25.447 | 24.387 |
| *Class* | Flavobacteriia | 0.001 | Yes | 0.255 | 0.000 | 0.000 | 0.000 | 0.000 |
| *Class* | Lentisphaeria | 0.001 | Yes | 0.075 | 0.000 | 0.000 | 0.000 | 0.000 |
| *Class* | Actinobacteria | 0.001 | Yes | 0.013 | 0.387 | 0.009 | 0.000 | 2.236 |
| *Class* | γ-Proteobacteria | 0.001 | Yes | 21.553 | 0.104 | 0.009 | 34.661 | 0.233 |
| *Class* | Negativicutes | 0.001 | Yes | 0.025 | 0.003 | 0.028 | 0.000 | 0.000 |
| *Class* | δ-Proteobacteria | 0.001 | Yes | 0.934 | 9.685 | 0.050 | 0.060 | 0.170 |
| *Class* | α-Proteobacteria | 0.001 | Yes | 0.003 | 0.580 | 0.340 | 0.029 | 0.038 |
| *Class* | Clostridia | 0.002 | Yes | 21.698 | 11.488 | 27.539 | 18.798 | 35.705 |
| *Class* | β-Proteobacteria | 0.002 | Yes | 0.000 | 0.327 | 0.091 | 0.031 | 0.019 |
| *Class* | unclassified | 0.002 | Yes | 0.000 | 0.000 | 0.264 | 0.021 | 0.035 |
| *Class* | ε-Proteobacteria | 0.004 | Yes | 0.008 | 0.008 | 0.000 | 0.000 | 0.000 |
| *Class* | Bacilli | 0.007 | Yes | 0.237 | 0.151 | 0.202 | 4.492 | 0.736 |
| *Class* | Deferribacteres | 0.007 | Yes | 5.958 | 5.464 | 8.644 | 3.566 | 8.153 |
| *Class* | Bacteroidia | 0.010 | Yes | 35.564 | 40.795 | 37.322 | 15.448 | 28.124 |
| *Class* | Verrucomicrobiae | 0.046 | Yes | 11.953 | 31.198 | 25.639 | 25.447 | 24.387 |
| *Order* | Flavobacteriales | 0.001 | Yes | 0.255 | 0.000 | 0.000 | 0.000 | 0.000 |
| *Order* | Victivallales | 0.001 | Yes | 0.075 | 0.000 | 0.000 | 0.000 | 0.000 |
| *Order* | Bifidobacteriales | 0.001 | Yes | 0.003 | 0.381 | 0.000 | 0.000 | 2.230 |
| *Order* | Enterobacteriales | 0.001 | Yes | 21.553 | 0.104 | 0.009 | 34.658 | 0.233 |
| *Order* | Selenomonadales | 0.001 | Yes | 0.025 | 0.003 | 0.028 | 0.000 | 0.000 |
| *Order* | Rhodospirillales | 0.001 | Yes | 0.000 | 0.580 | 0.337 | 0.029 | 0.038 |
| *Order* | Desulfovibrionales | 0.001 | Yes | 0.000 | 7.584 | 0.009 | 0.006 | 0.022 |
| *Order* | Clostridiales | 0.002 | Yes | 21.698 | 11.488 | 27.539 | 18.798 | 35.705 |
| *Order* | Burkholderiales | 0.003 | Yes | 0.000 | 0.327 | 0.091 | 0.031 | 0.019 |
| *Order* | unclassified | 0.003 | Yes | 0.000 | 0.000 | 0.264 | 0.021 | 0.035 |
| *Order* | Campylobacterales | 0.005 | Yes | 0.008 | 0.008 | 0.000 | 0.000 | 0.000 |
| *Order* | Lactobacillales | 0.008 | Yes | 0.237 | 0.151 | 0.202 | 4.492 | 0.736 |
| *Order* | Deferribacterales | 0.009 | Yes | 5.958 | 5.464 | 8.644 | 3.566 | 8.153 |
| *Order* | Bacteroidales | 0.012 | Yes | 35.564 | 40.795 | 37.322 | 15.448 | 28.124 |
| *Order* | Bdellovibrionales | 0.012 | Yes | 0.934 | 2.101 | 0.041 | 0.053 | 0.156 |
| *Order* | Coriobacteriales | 0.020 | Yes | 0.009 | 0.006 | 0.009 | 0.000 | 0.008 |
| *Order* | Rickettsiales | 0.028 | Yes | 0.003 | 0.000 | 0.003 | 0.000 | 0.000 |
| *Order* | Pseudomonadales | 0.028 | Yes | 0.001 | 0.000 | 0.000 | 0.001 | 0.000 |
| *Order* | Verrucomicrobiales | 0.046 | Yes | 11.953 | 31.198 | 25.639 | 25.447 | 24.387 |
| *Family* | Defluviitaleaceae | 0.001 | Yes | 0.000 | 0.000 | 0.013 | 0.000 | 0.000 |
| *Family* | Cryomorphaceae | 0.001 | Yes | 0.255 | 0.000 | 0.000 | 0.000 | 0.000 |
| *Family* | Victivallaceae | 0.001 | Yes | 0.075 | 0.000 | 0.000 | 0.000 | 0.000 |
| *Family* | Bifidobacteriaceae | 0.001 | Yes | 0.003 | 0.381 | 0.000 | 0.000 | 2.230 |
| *Family* | Enterobacteriaceae | 0.001 | Yes | 21.553 | 0.104 | 0.009 | 34.658 | 0.233 |
| *Family* | Veillonellaceae | 0.002 | Yes | 0.025 | 0.003 | 0.028 | 0.000 | 0.000 |
| *Family* | Rhodospirillaceae | 0.002 | Yes | 0.000 | 0.580 | 0.337 | 0.029 | 0.038 |
| *Family* | Eubacteriaceae | 0.002 | Yes | 0.475 | 1.655 | 1.592 | 0.066 | 0.204 |
| *Family* | Desulfovibrionaceae | 0.002 | Yes | 0.000 | 7.584 | 0.009 | 0.006 | 0.022 |
| *Family* | Porphyromonadaceae | 0.003 | Yes | 24.375 | 15.630 | 13.949 | 5.552 | 11.491 |
| *Family* | Peptococcaceae_1 | 0.003 | Yes | 6.256 | 0.132 | 7.546 | 3.514 | 4.756 |
| *Family* | Incertae_Sedis_XI | 0.003 | Yes | 0.008 | 0.000 | 0.000 | 0.000 | 0.000 |
| *Family* | Marinilabiliaceae | 0.003 | Yes | 0.000 | 0.000 | 0.006 | 0.000 | 0.000 |
| *Family* | Clostridiales_Incertae_Sedis_XI | 0.003 | Yes | 0.009 | 0.000 | 0.000 | 0.000 | 0.000 |
| *Family* | Sutterellaceae | 0.003 | Yes | 0.000 | 0.327 | 0.091 | 0.031 | 0.019 |
| *Family* | unclassified | 0.003 | Yes | 0.000 | 0.000 | 0.264 | 0.021 | 0.035 |
| *Family* | Ruminococcaceae | 0.004 | Yes | 5.656 | 2.922 | 6.090 | 4.963 | 10.697 |
| *Family* | Helicobacteraceae | 0.005 | Yes | 0.008 | 0.008 | 0.000 | 0.000 | 0.000 |
| *Family* | Rikenellaceae | 0.005 | Yes | 1.695 | 4.423 | 3.208 | 0.528 | 2.243 |
| *Family* | Clostridiales_Incertae_Sedis_XIII | 0.007 | Yes | 0.021 | 0.000 | 0.055 | 0.019 | 0.001 |
| *Family* | Lachnospiraceae | 0.007 | Yes | 9.525 | 6.508 | 11.975 | 9.679 | 20.657 |
| *Family* | Lactobacillaceae | 0.008 | Yes | 0.164 | 0.120 | 0.201 | 4.470 | 0.698 |
| *Family* | Deferribacteraceae | 0.009 | Yes | 5.958 | 5.464 | 8.644 | 3.566 | 8.153 |
| *Family* | Prevotellaceae | 0.011 | Yes | 0.000 | 0.047 | 0.003 | 0.000 | 0.006 |
| *Family* | Bdellovibrionaceae | 0.013 | Yes | 0.934 | 2.101 | 0.041 | 0.053 | 0.156 |
| *Family* | Clostridiaceae_4 | 0.015 | Yes | 0.003 | 0.000 | 0.000 | 0.000 | 0.000 |
| *Family* | Peptostreptococcaceae | 0.015 | Yes | 0.000 | 0.000 | 0.000 | 0.000 | 0.003 |
| *Family* | Streptococcaceae | 0.015 | Yes | 0.047 | 0.042 | 0.001 | 0.019 | 0.028 |
| *Family* | Coriobacteriaceae | 0.021 | Yes | 0.009 | 0.006 | 0.009 | 0.000 | 0.008 |
| *Family* | Bacteroidaceae | 0.025 | Yes | 9.220 | 20.666 | 20.147 | 8.559 | 14.385 |
| *Family* | Rickettsiaceae | 0.029 | Yes | 0.003 | 0.000 | 0.003 | 0.000 | 0.000 |
| *Family* | Moraxellaceae | 0.029 | Yes | 0.001 | 0.000 | 0.000 | 0.001 | 0.000 |
| *Family* | Verrucomicrobiaceae | 0.049 | Yes | 11.953 | 31.198 | 25.639 | 25.447 | 24.387 |
| *Family* | Clostridiales_Incertae_Sedis_XII | 0.049 | Yes | 0.001 | 0.003 | 0.001 | 0.000 | 0.019 |
| *Genus* | Defluviitalea | 0.002 | Yes | 0.000 | 0.000 | 0.013 | 0.000 | 0.000 |
| *Genus* | Butyricimonas | 0.002 | Yes | 7.776 | 0.000 | 0.000 | 0.000 | 0.000 |
| *Genus* | Wandonia | 0.002 | Yes | 0.255 | 0.000 | 0.000 | 0.000 | 0.000 |
| *Genus* | Victivallis | 0.002 | Yes | 0.075 | 0.000 | 0.000 | 0.000 | 0.000 |
| *Genus* | Tannerella | 0.002 | Yes | 0.000 | 0.000 | 1.982 | 0.808 | 1.595 |
| *Genus* | Bifidobacterium | 0.002 | Yes | 0.003 | 0.381 | 0.000 | 0.000 | 2.230 |
| *Genus* | Escherichia | 0.002 | Yes | 21.544 | 0.003 | 0.006 | 34.658 | 0.233 |
| *Genus* | Dorea | 0.002 | Yes | 0.015 | 0.035 | 0.145 | 0.418 | 1.047 |
| *Genus* | Enterobacter | 0.002 | Yes | 0.019 | 0.094 | 0.000 | 0.000 | 0.000 |
| *Genus* | Barnesiella | 0.002 | Yes | 0.001 | 0.009 | 8.635 | 3.375 | 7.521 |
| *Genus* | Anaerosinus | 0.003 | Yes | 0.025 | 0.003 | 0.028 | 0.000 | 0.000 |
| *Genus* | Insolitispirillum | 0.003 | Yes | 0.000 | 0.580 | 0.337 | 0.029 | 0.038 |
| *Genus* | Johnsonella | 0.003 | Yes | 0.023 | 0.000 | 0.001 | 0.000 | 0.035 |
| *Genus* | Oscillibacter | 0.003 | Yes | 0.676 | 0.488 | 1.324 | 2.181 | 2.969 |
| *Genus* | Eubacterium | 0.003 | Yes | 0.469 | 1.655 | 1.588 | 0.066 | 0.201 |
| *Genus* | Parabacteroides | 0.003 | Yes | 15.813 | 15.618 | 2.705 | 1.368 | 2.375 |
| *Genus* | Anaerotruncus | 0.003 | Yes | 0.937 | 0.208 | 0.226 | 0.459 | 0.396 |
| *Genus* | Subdoligranulum | 0.003 | Yes | 0.000 | 0.113 | 0.013 | 0.003 | 0.000 |
| *Genus* | Bilophila | 0.003 | Yes | 0.000 | 7.584 | 0.002 | 0.006 | 0.019 |
| *Genus* | Clostridium_IV | 0.003 | Yes | 2.233 | 0.566 | 1.019 | 0.729 | 1.894 |
| *Genus* | Ruminococcus2 | 0.003 | Yes | 0.563 | 0.135 | 0.211 | 0.154 | 0.657 |
| *Genus* | Roseburia | 0.003 | Yes | 0.072 | 0.009 | 0.019 | 0.000 | 0.003 |
| *Genus* | Coprobacillus | 0.003 | Yes | 0.000 | 0.019 | 0.000 | 0.000 | 0.000 |
| *Genus* | Dethiosulfatibacter | 0.003 | Yes | 0.008 | 0.000 | 0.000 | 0.000 | 0.000 |
| *Genus* | Clostridium_III | 0.003 | Yes | 0.008 | 0.000 | 0.000 | 0.000 | 0.000 |
| *Genus* | Alkalitalea | 0.003 | Yes | 0.000 | 0.000 | 0.006 | 0.000 | 0.000 |
| *Genus* | Paraprevotella | 0.003 | Yes | 0.000 | 0.047 | 0.000 | 0.000 | 0.000 |
| *Genus* | Sporanaerobacter | 0.003 | Yes | 0.009 | 0.000 | 0.000 | 0.000 | 0.000 |
| *Genus* | Pseudobutyrivibrio | 0.003 | Yes | 0.289 | 0.000 | 0.000 | 0.000 | 0.000 |
| *Genus* | Parasutterella | 0.003 | Yes | 0.000 | 0.327 | 0.091 | 0.031 | 0.019 |
| *Genus* | Acetivibrio | 0.003 | Yes | 0.013 | 0.009 | 0.252 | 0.000 | 0.075 |
| *Genus* | Peptococcus | 0.004 | Yes | 6.035 | 0.003 | 6.920 | 3.161 | 4.313 |
| *Genus* | unclassified | 0.004 | Yes | 0.000 | 0.000 | 0.264 | 0.021 | 0.035 |
| *Genus* | Desulfitibacter | 0.004 | Yes | 0.359 | 0.132 | 0.626 | 0.322 | 0.651 |
| *Genus* | Pseudoflavonifractor | 0.006 | Yes | 0.692 | 0.862 | 2.265 | 1.076 | 2.937 |
| *Genus* | Lactonifactor | 0.006 | Yes | 0.014 | 0.000 | 0.009 | 0.000 | 0.000 |
| *Genus* | Helicobacter | 0.006 | Yes | 0.008 | 0.008 | 0.000 | 0.000 | 0.000 |
| *Genus* | Alistipes | 0.006 | Yes | 1.695 | 4.423 | 3.208 | 0.528 | 2.243 |
| *Genus* | Sporobacter | 0.006 | Yes | 0.025 | 0.003 | 0.025 | 0.025 | 0.009 |
| *Genus* | Clostridium_XlVa | 0.006 | Yes | 3.765 | 1.667 | 4.325 | 2.716 | 6.122 |
| *Genus* | Flavonifractor | 0.007 | Yes | 0.230 | 0.340 | 0.521 | 0.247 | 1.431 |
| *Genus* | Coprococcus | 0.007 | Yes | 0.135 | 0.069 | 0.038 | 0.068 | 0.189 |
| *Genus* | Shuttleworthia | 0.007 | Yes | 0.972 | 0.021 | 0.214 | 0.239 | 0.277 |
| *Genus* | Anaerovorax | 0.008 | Yes | 0.021 | 0.000 | 0.055 | 0.019 | 0.001 |
| *Genus* | Marvinbryantia | 0.008 | Yes | 2.362 | 4.055 | 6.228 | 3.797 | 10.427 |
| *Genus* | Lactobacillus | 0.008 | Yes | 0.164 | 0.120 | 0.201 | 4.470 | 0.698 |
| *Genus* | Parasporobacterium | 0.010 | Yes | 0.206 | 0.000 | 0.151 | 0.094 | 0.132 |
| *Genus* | Saccharofermentans | 0.010 | Yes | 0.000 | 0.000 | 0.008 | 0.003 | 0.000 |
| *Genus* | Prevotella | 0.010 | Yes | 0.000 | 0.000 | 0.003 | 0.000 | 0.006 |
| *Genus* | Alkalibacter | 0.010 | Yes | 0.006 | 0.000 | 0.000 | 0.000 | 0.001 |
| *Genus* | Mucispirillum | 0.010 | Yes | 5.958 | 5.464 | 8.644 | 3.566 | 8.153 |
| *Genus* | Acetanaerobacterium | 0.011 | Yes | 0.176 | 0.013 | 0.013 | 0.016 | 0.028 |
| *Genus* | Syntrophococcus | 0.011 | Yes | 0.519 | 0.208 | 0.037 | 1.186 | 0.815 |
| *Genus* | Vampirovibrio | 0.014 | Yes | 0.934 | 2.101 | 0.041 | 0.053 | 0.156 |
| *Genus* | Lactococcus | 0.015 | Yes | 0.047 | 0.042 | 0.001 | 0.019 | 0.025 |
| *Genus* | Butyricicoccus | 0.015 | Yes | 0.003 | 0.006 | 0.003 | 0.000 | 0.016 |
| *Genus* | Hydrogenoanaerobacterium | 0.015 | Yes | 0.324 | 0.245 | 0.434 | 0.091 | 0.469 |
| *Genus* | Holdemania | 0.015 | Yes | 0.008 | 0.000 | 0.003 | 0.000 | 0.009 |
| *Genus* | Odoribacter | 0.015 | Yes | 0.000 | 0.003 | 0.000 | 0.000 | 0.000 |
| *Genus* | Streptococcus | 0.015 | Yes | 0.000 | 0.000 | 0.000 | 0.000 | 0.003 |
| *Genus* | Thermotalea | 0.015 | Yes | 0.003 | 0.000 | 0.000 | 0.000 | 0.000 |
| *Genus* | Alkalibaculum | 0.015 | Yes | 0.000 | 0.000 | 0.003 | 0.000 | 0.000 |
| *Genus* | Clostridium_XI | 0.015 | Yes | 0.000 | 0.000 | 0.000 | 0.000 | 0.003 |
| *Genus* | Kandleria | 0.015 | Yes | 0.003 | 0.000 | 0.000 | 0.000 | 0.000 |
| *Genus* | Clostridium_sensu_stricto | 0.015 | Yes | 0.000 | 0.000 | 0.001 | 0.000 | 0.000 |
| *Genus* | Robinsoniella | 0.016 | Yes | 0.038 | 0.126 | 0.261 | 0.154 | 0.384 |
| *Genus* | Papillibacter | 0.018 | Yes | 0.000 | 0.000 | 0.003 | 0.013 | 0.001 |
| *Genus* | Allobaculum | 0.019 | Yes | 0.013 | 0.007 | 0.005 | 0.000 | 0.000 |
| *Genus* | Enterorhabdus | 0.021 | Yes | 0.009 | 0.006 | 0.009 | 0.000 | 0.008 |
| *Genus* | Bacteroides | 0.025 | Yes | 9.220 | 20.666 | 20.147 | 8.559 | 14.381 |
| *Genus* | Cellulosilyticum | 0.029 | Yes | 0.006 | 0.000 | 0.003 | 0.000 | 0.000 |
| *Genus* | Proteus | 0.029 | Yes | 0.000 | 0.001 | 0.003 | 0.000 | 0.000 |
| *Genus* | Orientia | 0.029 | Yes | 0.003 | 0.000 | 0.003 | 0.000 | 0.000 |
| *Genus* | Desulfovibrio | 0.029 | Yes | 0.000 | 0.000 | 0.003 | 0.000 | 0.003 |
| *Genus* | Anaerorhabdus | 0.029 | Yes | 0.000 | 0.000 | 0.000 | 0.001 | 0.001 |
| *Genus* | Acinetobacter | 0.029 | Yes | 0.001 | 0.000 | 0.000 | 0.001 | 0.000 |
| *Genus* | Moryella | 0.033 | Yes | 0.079 | 0.079 | 0.009 | 0.002 | 0.038 |
| *Genus* | Clostridium_XlVb | 0.036 | Yes | 0.145 | 0.098 | 0.208 | 0.198 | 0.075 |
| *Genus* | Akkermansia | 0.048 | Yes | 11.953 | 31.198 | 25.639 | 25.447 | 24.387 |
| *Genus* | Acidaminobacter | 0.049 | Yes | 0.001 | 0.003 | 0.001 | 0.000 | 0.019 |

M, male; F, female; E2, 17β-estradiol; OVX, ovariectomized; n=5/ group. Differential abundance analysis (non parametric ANOVA with false discovery rate (FDR) corrected p<0.05 for multiple comparisons) was conducted using relative abundance (RA) calculated from whole microbiota OTUs table. Table shows the mean RA for each group. Taxa with FDR corrected p value of >0.05 are considered non significant.

**Table S3. Pair wise differential abundance analysis between groups.**

PHYLUM

|  | Mean1 | Mean2 |  |  |  |
| --- | --- | --- | --- | --- | --- |
| **Taxonomy** | **Male (M)** | **Female (F)** | **Difference** | **P value** | **FDR value** |
| Lentisphaerae | 0.074860 | 0.000000 | 0.074860 | 0.000119 | 0.000127 |
| Actinobacteria | 0.012790 | 0.398200 | -0.385400 | 0.000134 | 0.000127 |
| Proteobacteria | 23.760000 | 10.880000 | 12.880000 | 0.000181 | 0.000127 |
| Verrucomicrobia | 12.400000 | 30.970000 | -18.570000 | 0.000279 | 0.000147 |
| Firmicutes | 22.050000 | 12.230000 | 9.825000 | 0.005265 | 0.002211 |
| **Taxonomy** | **Male (M)** | **M+E2** | **Difference** | **P value** | **FDR value** |
| Proteobacteria | 23.760000 | 0.515200 | 23.240000 | 0.000000 | 0.000001 |
| Lentisphaerae | 0.074860 | 0.000000 | 0.074860 | 0.000119 | 0.000187 |
| Verrucomicrobia | 12.400000 | 25.710000 | -13.310000 | 0.003043 | 0.003195 |
| Deferribacteres | 5.904000 | 9.384000 | -3.480000 | 0.022101 | 0.014011 |
| Candidatus_Saccharibacteria | 0.000000 | 0.297600 | -0.297600 | 0.022239 | 0.014011 |
| **Taxonomy** | **Male (M)** | **OVX** | **Difference** | **P value** | **FDR value** |
| Bacteroidetes | 35.800000 | 15.100000 | 20.690000 | 0.000076 | 0.000123 |
| Actinobacteria | 0.012790 | 0.000000 | 0.012790 | 0.000078 | 0.000123 |
| Lentisphaerae | 0.074860 | 0.000000 | 0.074860 | 0.000119 | 0.000125 |
| Proteobacteria | 23.760000 | 34.620000 | -10.860000 | 0.000353 | 0.000278 |
| Deferribacteres | 5.904000 | 4.123000 | 1.781000 | 0.025648 | 0.016158 |
| Verrucomicrobia | 12.400000 | 22.330000 | -9.931000 | 0.040036 | 0.021019 |
| Candidatus_Saccharibacteria | 0.000000 | 0.039420 | -0.039420 | 0.080771 | 0.036347 |
| Firmicutes | 22.050000 | 23.780000 | -1.730000 | 0.110673 | 0.043578 |
| **Taxonomy** | **Male (M)** | **OVX+E2** | **Difference** | **P value** | **FDR value** |
| Proteobacteria | 23.760000 | 0.450200 | 23.310000 | 0.000000 | 0.000001 |
| Firmicutes | 22.050000 | 36.830000 | -14.780000 | 0.000000 | 0.000001 |
| Actinobacteria | 0.012790 | 2.244000 | -2.231000 | 0.000003 | 0.000003 |
| Lentisphaerae | 0.074860 | 0.000000 | 0.074860 | 0.000119 | 0.000094 |
| Candidatus_Saccharibacteria | 0.000000 | 0.033760 | -0.033760 | 0.008006 | 0.005044 |
| Deferribacteres | 5.904000 | 8.249000 | -2.344000 | 0.091508 | 0.048042 |
| **Taxonomy** | **F** | **M+E2** | **Difference** | **P value** | **FDR value** |
| Proteobacteria | 10.880000 | 0.515200 | 10.360000 | 0.000027 | 0.000056 |
| Actinobacteria | 0.398200 | 0.008808 | 0.389400 | 0.000125 | 0.000132 |
| Firmicutes | 12.230000 | 27.660000 | -15.430000 | 0.010869 | 0.007608 |
| Deferribacteres | 5.090000 | 9.384000 | -4.294000 | 0.020658 | 0.009340 |
| Candidatus_Saccharibacteria | 0.000000 | 0.297600 | -0.297600 | 0.022239 | 0.009340 |
| Verrucomicrobia | 30.970000 | 25.710000 | 5.261000 | 0.108895 | 0.038113 |
| **Taxonomy** | **F** | **OVX** | **Difference** | **P value** | **FDR value** |
| Proteobacteria | 10.880000 | 34.620000 | -23.740000 | 0.000000 | 0.000001 |
| Actinobacteria | 0.398200 | 0.000000 | 0.398200 | 0.000106 | 0.000138 |
| Bacteroidetes | 40.430000 | 15.100000 | 25.330000 | 0.000131 | 0.000138 |
| Firmicutes | 12.230000 | 23.780000 | -11.560000 | 0.002935 | 0.002311 |
| Verrucomicrobia | 30.970000 | 22.330000 | 8.641000 | 0.055296 | 0.034837 |
| Candidatus_Saccharibacteria | 0.000000 | 0.039420 | -0.039420 | 0.080771 | 0.042405 |
| **Taxonomy** | **F** | **OVX+E2** | **Difference** | **P value** | **FDR value** |
| Actinobacteria | 0.398200 | 2.244000 | -1.846000 | 0.000018 | 0.000026 |
| Firmicutes | 12.230000 | 36.830000 | -24.600000 | 0.000020 | 0.000026 |
| Proteobacteria | 10.880000 | 0.450200 | 10.430000 | 0.000025 | 0.000026 |
| Candidatus_Saccharibacteria | 0.000000 | 0.033760 | -0.033760 | 0.008006 | 0.006305 |
| Deferribacteres | 5.090000 | 8.249000 | -3.159000 | 0.066589 | 0.041951 |
| Bacteroidetes | 40.430000 | 28.870000 | 11.560000 | 0.091963 | 0.048281 |
| **Taxonomy** | **M+E2** | **OVX** | **Difference** | **P value** | **FDR value** |
| Proteobacteria | 0.515200 | 34.620000 | -34.100000 | 0.000000 | 0.000000 |
| Bacteroidetes | 36.420000 | 15.100000 | 21.320000 | 0.000244 | 0.000385 |
| Deferribacteres | 9.384000 | 4.123000 | 5.261000 | 0.004333 | 0.004550 |
| Actinobacteria | 0.008808 | 0.000000 | 0.008808 | 0.020260 | 0.015955 |
| Candidatus_Saccharibacteria | 0.297600 | 0.039420 | 0.258100 | 0.042455 | 0.026747 |
| **Taxonomy** | **M+E2** | **OVX+E2** | **Difference** | **P value** | **FDR value** |
| Actinobacteria | 0.008808 | 2.244000 | -2.235000 | 0.000003 | 0.000020 |
| **Taxonomy** | **OVX** | **OVX+E2** | **Difference** | **P value** | **FDR value** |
| Proteobacteria | 34.620000 | 0.450200 | 34.170000 | 0.000000 | 0.000000 |
| Actinobacteria | 0.000000 | 2.244000 | -2.244000 | 0.000003 | 0.000005 |
| Firmicutes | 23.780000 | 36.830000 | -13.050000 | 0.000012 | 0.000012 |
| Deferribacteres | 4.123000 | 8.249000 | -4.126000 | 0.014710 | 0.011584 |
| Bacteroidetes | 15.100000 | 28.870000 | -13.770000 | 0.039527 | 0.024902 |

CLASS

|  | Mean1 | Mean2 |  |  |  |
| --- | --- | --- | --- | --- | --- |
| **Taxonomy** | **Male (M)** | **Female (F)** | **Difference** | **P value** | **FDR value** |
| γ-Proteobacteria | 22.610000 | 0.108200 | 22.500000 | 0.000000 | 0.000000 |
| δ-Proteobacteria | 1.137000 | 9.683000 | -8.546000 | 0.000068 | 0.000176 |
| Lentisphaeria | 0.074860 | 0.000000 | 0.074860 | 0.000119 | 0.000176 |
| Actinobacteria | 0.012790 | 0.398200 | -0.385400 | 0.000134 | 0.000176 |
| Verrucomicrobiae | 12.400000 | 30.970000 | -18.570000 | 0.000279 | 0.000293 |
| β-Proteobacteria | 0.000000 | 0.320000 | -0.320000 | 0.003352 | 0.002520 |
| Flavobacteriia | 0.265900 | 0.000000 | 0.265900 | 0.003359 | 0.002520 |
| α-Proteobacteria | 0.003355 | 0.760200 | -0.756800 | 0.004433 | 0.002778 |
| Clostridia | 21.710000 | 11.920000 | 9.788000 | 0.004763 | 0.002778 |
| Negativicutes | 0.025580 | 0.003355 | 0.022230 | 0.008836 | 0.004639 |
| **Taxonomy** | **Male (M)** | **M+E2** | **Difference** | **P value** | **FDR value** |
| γ-Proteobacteria | 22.610000 | 0.010070 | 22.600000 | 0.000000 | 0.000001 |
| Lentisphaeria | 0.074860 | 0.000000 | 0.074860 | 0.000119 | 0.000437 |
| Verrucomicrobiae | 12.400000 | 25.710000 | -13.310000 | 0.003043 | 0.006173 |
| Flavobacteriia | 0.265900 | 0.000000 | 0.265900 | 0.003359 | 0.006173 |
| ε-Proteobacteria | 0.006711 | 0.000000 | 0.006711 | 0.005355 | 0.007872 |
| α-Proteobacteria | 0.003355 | 0.356100 | -0.352700 | 0.014279 | 0.016586 |
| δ-Proteobacteria | 1.137000 | 0.052430 | 1.084000 | 0.015796 | 0.016586 |
| Deferribacteres | 5.904000 | 9.384000 | -3.480000 | 0.022101 | 0.018162 |
| unclassified | 0.000000 | 0.297600 | -0.297600 | 0.022239 | 0.018162 |
| β-Proteobacteria | 0.000000 | 0.096670 | -0.096670 | 0.052891 | 0.038875 |
| **Taxonomy** | **Male (M)** | **OVX** | **Difference** | **P value** | **FDR value** |
| γ-Proteobacteria | 22.610000 | 34.470000 | -11.860000 | 0.000061 | 0.000110 |
| Actinobacteria | 0.012790 | 0.000000 | 0.012790 | 0.000078 | 0.000110 |
| Bacteroidia | 35.530000 | 15.100000 | 20.430000 | 0.000079 | 0.000110 |
| Lentisphaeria | 0.074860 | 0.000000 | 0.074860 | 0.000119 | 0.000125 |
| Β-Proteobacteria | 0.000000 | 0.030410 | -0.030410 | 0.001310 | 0.001101 |
| Flavobacteriia | 0.265900 | 0.000000 | 0.265900 | 0.003359 | 0.002037 |
| Negativicutes | 0.025580 | 0.000000 | 0.025580 | 0.003395 | 0.002037 |
| ε-Proteobacteria | 0.006711 | 0.000000 | 0.006711 | 0.005355 | 0.002811 |
| Clostridia | 21.710000 | 19.050000 | 2.659000 | 0.007057 | 0.003293 |
| Bacilli | 0.216000 | 4.639000 | -4.423000 | 0.010679 | 0.004485 |
| δ-Proteobacteria | 1.137000 | 0.059980 | 1.077000 | 0.016334 | 0.006237 |
| Deferribacteres | 5.904000 | 4.123000 | 1.781000 | 0.025648 | 0.008977 |
| Verrucomicrobiae | 12.400000 | 22.330000 | -9.931000 | 0.040036 | 0.012935 |
| unclassified | 0.000000 | 0.039420 | -0.039420 | 0.080771 | 0.024231 |
| α-Proteobacteria | 0.003355 | 0.053680 | -0.050330 | 0.090530 | 0.025348 |
| **Taxonomy** | **Male (M)** | **OVX+E2** | **Difference** | **P value** | **FDR value** |
| γ-Proteobacteria | 22.610000 | 0.243900 | 22.360000 | 0.000000 | 0.000001 |
| Clostridia | 21.710000 | 35.950000 | -14.240000 | 0.000003 | 0.000006 |
| Actinobacteria | 0.012790 | 2.244000 | -2.231000 | 0.000003 | 0.000006 |
| Lentisphaeria | 0.074860 | 0.000000 | 0.074860 | 0.000119 | 0.000156 |
| Flavobacteriia | 0.265900 | 0.000000 | 0.265900 | 0.003359 | 0.002970 |
| Negativicutes | 0.025580 | 0.000000 | 0.025580 | 0.003395 | 0.002970 |
| ε-Proteobacteria | 0.006711 | 0.000000 | 0.006711 | 0.005355 | 0.004016 |
| unclassified | 0.000000 | 0.033760 | -0.033760 | 0.008006 | 0.004686 |
| β-Proteobacteria | 0.000000 | 0.018240 | -0.018240 | 0.008033 | 0.004686 |
| δ-Proteobacteria | 1.137000 | 0.149100 | 0.987700 | 0.024362 | 0.012790 |
| α-Proteobacteria | 0.003355 | 0.039010 | -0.035650 | 0.026898 | 0.012838 |
| Bacilli | 0.216000 | 0.740300 | -0.524300 | 0.053874 | 0.023570 |
| Deferribacteres | 5.904000 | 8.249000 | -2.344000 | 0.091508 | 0.036955 |
| **Taxonomy** | **F** | **M+E2** | **Difference** | **P value** | **FDR value** |
| δ-Proteobacteria | 9.683000 | 0.052430 | 9.630000 | 0.000020 | 0.000124 |
| Actinobacteria | 0.398200 | 0.008808 | 0.389400 | 0.000125 | 0.000395 |
| γ-Proteobacteria | 0.108200 | 0.010070 | 0.098140 | 0.003222 | 0.006747 |
| Negativicutes | 0.003355 | 0.035860 | -0.032500 | 0.004446 | 0.006747 |
| ε-Proteobacteria | 0.006711 | 0.000000 | 0.006711 | 0.005355 | 0.006747 |
| Clostridia | 11.920000 | 27.190000 | -15.270000 | 0.010076 | 0.010580 |
| Deferribacteres | 5.090000 | 9.384000 | -4.294000 | 0.020658 | 0.017513 |
| unclassified | 0.000000 | 0.297600 | -0.297600 | 0.022239 | 0.017513 |
| β-Proteobacteria | 0.320000 | 0.096670 | 0.223300 | 0.035785 | 0.025050 |
| Erysipelotrichia | 0.150400 | 0.067730 | 0.082620 | 0.056686 | 0.035712 |
| **Taxonomy** | **F** | **OVX** | **Difference** | **P value** | **FDR value** |
| γ-Proteobacteria | 0.108200 | 34.470000 | -34.360000 | 0.000000 | 0.000000 |
| δ-Proteobacteria | 9.683000 | 0.059980 | 9.623000 | 0.000020 | 0.000052 |
| Actinobacteria | 0.398200 | 0.000000 | 0.398200 | 0.000106 | 0.000172 |
| Bacteroidia | 40.430000 | 15.100000 | 25.330000 | 0.000131 | 0.000172 |
| ε-Proteobacteria | 0.006711 | 0.000000 | 0.006711 | 0.005355 | 0.005055 |
| β-Proteobacteria | 0.320000 | 0.030410 | 0.289600 | 0.005916 | 0.005055 |
| α-Proteobacteria | 0.760200 | 0.053680 | 0.706500 | 0.006741 | 0.005055 |
| Bacilli | 0.156000 | 4.639000 | -4.483000 | 0.010001 | 0.006563 |
| Clostridia | 11.920000 | 19.050000 | -7.128000 | 0.026005 | 0.015169 |
| Verrucomicrobiae | 30.970000 | 22.330000 | 8.641000 | 0.055296 | 0.029031 |
| unclassified | 0.000000 | 0.039420 | -0.039420 | 0.080771 | 0.038550 |
| Negativicutes | 0.003355 | 0.000000 | 0.003355 | 0.089277 | 0.039059 |
| **Taxonomy** | **F** | **OVX+E2** | **Difference** | **P value** | **FDR value** |
| Actinobacteria | 0.398200 | 2.244000 | -1.846000 | 0.000018 | 0.000063 |
| δ-Proteobacteria | 9.683000 | 0.149100 | 9.534000 | 0.000021 | 0.000063 |
| Clostridia | 11.920000 | 35.950000 | -24.030000 | 0.000026 | 0.000063 |
| β-Proteobacteria | 0.320000 | 0.018240 | 0.301800 | 0.004709 | 0.007144 |
| ε-Proteobacteria | 0.006711 | 0.000000 | 0.006711 | 0.005355 | 0.007144 |
| α-Proteobacteria | 0.760200 | 0.039010 | 0.721200 | 0.005832 | 0.007144 |
| unclassified | 0.000000 | 0.033760 | -0.033760 | 0.008006 | 0.008406 |
| Bacilli | 0.156000 | 0.740300 | -0.584200 | 0.037119 | 0.034103 |
| **Taxonomy** | **M+E2** | **OVX** | **Difference** | **P value** | **FDR value** |
| γ-Proteobacteria | 0.010070 | 34.470000 | -34.460000 | 0.000000 | 0.000000 |
| Bacteroidia | 36.420000 | 15.100000 | 21.320000 | 0.000244 | 0.000898 |
| Negativicutes | 0.035860 | 0.000000 | 0.035860 | 0.002234 | 0.005474 |
| Deferribacteres | 9.384000 | 4.123000 | 5.261000 | 0.004333 | 0.007962 |
| Bacilli | 0.367600 | 4.639000 | -4.271000 | 0.013178 | 0.019372 |
| Actinobacteria | 0.008808 | 0.000000 | 0.008808 | 0.020260 | 0.024819 |
| α-Proteobacteria | 0.356100 | 0.053680 | 0.302400 | 0.031388 | 0.032957 |
| unclassified | 0.297600 | 0.039420 | 0.258100 | 0.042455 | 0.039006 |
| **Taxonomy** | **M+E2** | **OVX+E2** | **Difference** | **P value** | **FDR value** |
| Actinobacteria | 0.008808 | 2.244000 | -2.235000 | 0.000003 | 0.000037 |
| Negativicutes | 0.035860 | 0.000000 | 0.035860 | 0.002234 | 0.012902 |
| **Taxonomy** | **OVX** | **OVX+E2** | **Difference** | **P value** | **FDR value** |
| γ-Proteobacteria | 34.470000 | 0.243900 | 34.230000 | 0.000000 | 0.000000 |
| Clostridia | 19.050000 | 35.950000 | -16.900000 | 0.000002 | 0.000008 |
| Actinobacteria | 0.000000 | 2.244000 | -2.244000 | 0.000003 | 0.000009 |
| Deferribacteres | 4.123000 | 8.249000 | -4.126000 | 0.014710 | 0.030892 |
| Bacilli | 4.639000 | 0.740300 | 3.899000 | 0.020625 | 0.034650 |
| δ-Proteobacteria | 0.059980 | 0.149100 | -0.089120 | 0.037458 | 0.047432 |
| Bacteroidia | 15.100000 | 28.870000 | -13.770000 | 0.039527 | 0.047432 |

ORDER

|  | Mean1 | Mean2 |  |  |  |
| --- | --- | --- | --- | --- | --- |
| **Taxonomy** | **Male (M)** | **Female (F)** | **Difference** | **P value** | **FDR value** |
| Desulfovibrionales | 0.0000000 | 7.5500000 | -7.5500000 | 0.0000000 | 0.0000004 |
| Enterobacteriales | 22.6100000 | 0.1082000 | 22.5000000 | 0.0000001 | 0.0000004 |
| Victivallales | 0.0748600 | 0.0000000 | 0.0748600 | 0.0001189 | 0.0003590 |
| Bifidobacteriales | 0.0033550 | 0.3921000 | -0.3888000 | 0.0001520 | 0.0003590 |
| Verrucomicrobiales | 12.4000000 | 30.9700000 | -18.5700000 | 0.0002793 | 0.0005280 |
| Burkholderiales | 0.0000000 | 0.3200000 | -0.3200000 | 0.0033522 | 0.0045352 |
| Flavobacteriales | 0.2659000 | 0.0000000 | 0.2659000 | 0.0033594 | 0.0045352 |
| Rhodospirillales | 0.0000000 | 0.7602000 | -0.7602000 | 0.0043255 | 0.0050006 |
| Clostridiales | 21.7100000 | 11.9200000 | 9.7880000 | 0.0047625 | 0.0050006 |
| Selenomonadales | 0.0255800 | 0.0033550 | 0.0222300 | 0.0088359 | 0.0083499 |
| **Taxonomy** | **Male (M)** | **M+E2** | **Difference** | **P value** | **FDR value** |
| Enterobacteriales | 22.6100000 | 0.0100700 | 22.6000000 | 0.0000001 | 0.0000009 |
| Victivallales | 0.0748600 | 0.0000000 | 0.0748600 | 0.0001189 | 0.0006241 |
| Verrucomicrobiales | 12.4000000 | 25.7100000 | -13.3100000 | 0.0030433 | 0.0088184 |
| Flavobacteriales | 0.2659000 | 0.0000000 | 0.2659000 | 0.0033594 | 0.0088184 |
| Campylobacterales | 0.0067110 | 0.0000000 | 0.0067110 | 0.0053548 | 0.0112451 |
| Rhodospirillales | 0.0000000 | 0.3527000 | -0.3527000 | 0.0146615 | 0.0228509 |
| Bdellovibrionales | 1.1370000 | 0.0436200 | 1.0930000 | 0.0152340 | 0.0228509 |
| Desulfovibrionales | 0.0000000 | 0.0088080 | -0.0088080 | 0.0202601 | 0.0233511 |
| Deferribacterales | 5.9040000 | 9.3840000 | -3.4800000 | 0.0221011 | 0.0233511 |
| unclassified | 0.0000000 | 0.2976000 | -0.2976000 | 0.0222391 | 0.0233511 |
| **Taxonomy** | **Male (M)** | **OVX** | **Difference** | **P value** | **FDR value** |
| Enterobacteriales | 22.6100000 | 34.4700000 | -11.8600000 | 0.0000600 | 0.0003309 |
| Bacteroidales | 35.5300000 | 15.1000000 | 20.4300000 | 0.0000788 | 0.0003309 |
| Victivallales | 0.0748600 | 0.0000000 | 0.0748600 | 0.0001189 | 0.0003328 |
| Burkholderiales | 0.0000000 | 0.0304100 | -0.0304100 | 0.0013101 | 0.0027513 |
| Flavobacteriales | 0.2659000 | 0.0000000 | 0.2659000 | 0.0033594 | 0.0047525 |
| Selenomonadales | 0.0255800 | 0.0000000 | 0.0255800 | 0.0033947 | 0.0047525 |
| Campylobacterales | 0.0067110 | 0.0000000 | 0.0067110 | 0.0053548 | 0.0064258 |
| Clostridiales | 21.7100000 | 19.0500000 | 2.6590000 | 0.0070574 | 0.0074103 |
| Lactobacillales | 0.2160000 | 4.6390000 | -4.4230000 | 0.0106789 | 0.0099670 |
| Bdellovibrionales | 1.1370000 | 0.0532600 | 1.0840000 | 0.0158516 | 0.0133154 |
| Deferribacterales | 5.9040000 | 4.1230000 | 1.7810000 | 0.0256477 | 0.0195855 |
| Verrucomicrobiales | 12.4000000 | 22.3300000 | -9.9310000 | 0.0400357 | 0.0280250 |
| Rhodospirillales | 0.0000000 | 0.0536800 | -0.0536800 | 0.0737027 | 0.0441148 |
| unclassified | 0.0000000 | 0.0394200 | -0.0394200 | 0.0807714 | 0.0441148 |
| Bifidobacteriales | 0.0033550 | 0.0000000 | 0.0033550 | 0.0892770 | 0.0441148 |
| Rickettsiales | 0.0033550 | 0.0000000 | 0.0033550 | 0.0892770 | 0.0441148 |
| Desulfovibrionales | 0.0000000 | 0.0067100 | -0.0067100 | 0.0892799 | 0.0441148 |
| **Taxonomy** | **Male (M)** | **OVX+E2** | **Difference** | **P value** | **FDR value** |
| Enterobacteriales | 22.6100000 | 0.2439000 | 22.3600000 | 0.0000001 | 0.0000008 |
| Clostridiales | 21.7100000 | 35.9500000 | -14.2400000 | 0.0000027 | 0.0000090 |
| Bifidobacteriales | 0.0033550 | 2.2370000 | -2.2340000 | 0.0000032 | 0.0000090 |
| Victivallales | 0.0748600 | 0.0000000 | 0.0748600 | 0.0001189 | 0.0002496 |
| Desulfovibrionales | 0.0000000 | 0.0222300 | -0.0222300 | 0.0012379 | 0.0020797 |
| Flavobacteriales | 0.2659000 | 0.0000000 | 0.2659000 | 0.0033594 | 0.0040736 |
| Selenomonadales | 0.0255800 | 0.0000000 | 0.0255800 | 0.0033947 | 0.0040736 |
| Campylobacterales | 0.0067110 | 0.0000000 | 0.0067110 | 0.0053548 | 0.0056226 |
| unclassified | 0.0000000 | 0.0337600 | -0.0337600 | 0.0080058 | 0.0067477 |
| Burkholderiales | 0.0000000 | 0.0182400 | -0.0182400 | 0.0080329 | 0.0067477 |
| Rhodospirillales | 0.0000000 | 0.0390100 | -0.0390100 | 0.0174743 | 0.0133440 |
| Bdellovibrionales | 1.1370000 | 0.1269000 | 1.0100000 | 0.0220871 | 0.0154610 |
| Lactobacillales | 0.2160000 | 0.7403000 | -0.5243000 | 0.0538739 | 0.0348108 |
| **Taxonomy** | **F** | **M+E2** | **Difference** | **P value** | **FDR value** |
| Desulfovibrionales | 7.5500000 | 0.0088080 | 7.5410000 | 0.0000000 | 0.0000003 |
| Bifidobacteriales | 0.3921000 | 0.0000000 | 0.3921000 | 0.0001428 | 0.0005996 |
| Enterobacteriales | 0.1082000 | 0.0100700 | 0.0981400 | 0.0032218 | 0.0089961 |
| Selenomonadales | 0.0033550 | 0.0358600 | -0.0325000 | 0.0044459 | 0.0089961 |
| Campylobacterales | 0.0067110 | 0.0000000 | 0.0067110 | 0.0053548 | 0.0089961 |
| Clostridiales | 11.9200000 | 27.1900000 | -15.2700000 | 0.0100761 | 0.0141066 |
| Deferribacterales | 5.0900000 | 9.3840000 | -4.2940000 | 0.0206582 | 0.0216821 |
| unclassified | 0.0000000 | 0.2976000 | -0.2976000 | 0.0222391 | 0.0216821 |
| Bdellovibrionales | 2.1330000 | 0.0436200 | 2.0890000 | 0.0232308 | 0.0216821 |
| Burkholderiales | 0.3200000 | 0.0966700 | 0.2233000 | 0.0357852 | 0.0300595 |
| Erysipelotrichales | 0.1504000 | 0.0677300 | 0.0826200 | 0.0566860 | 0.0432875 |
| **Taxonomy** | **F** | **OVX** | **Difference** | **P value** | **FDR value** |
| Enterobacteriales | 0.1082000 | 34.4700000 | -34.3600000 | 0.0000000 | 0.0000000 |
| Desulfovibrionales | 7.5500000 | 0.0067100 | 7.5430000 | 0.0000000 | 0.0000001 |
| Bacteroidales | 40.4300000 | 15.1000000 | 25.3300000 | 0.0001310 | 0.0002249 |
| Bifidobacteriales | 0.3921000 | 0.0000000 | 0.3921000 | 0.0001428 | 0.0002249 |
| Campylobacterales | 0.0067110 | 0.0000000 | 0.0067110 | 0.0053548 | 0.0060665 |
| Burkholderiales | 0.3200000 | 0.0304100 | 0.2896000 | 0.0059158 | 0.0060665 |
| Rhodospirillales | 0.7602000 | 0.0536800 | 0.7065000 | 0.0067406 | 0.0060665 |
| Coriobacteriales | 0.0060820 | 0.0000000 | 0.0060820 | 0.0080329 | 0.0063259 |
| Lactobacillales | 0.1560000 | 4.6390000 | -4.4830000 | 0.0100011 | 0.0070008 |
| Bdellovibrionales | 2.1330000 | 0.0532600 | 2.0800000 | 0.0236912 | 0.0148935 |
| Clostridiales | 11.9200000 | 19.0500000 | -7.1280000 | 0.0260045 | 0.0148935 |
| Verrucomicrobiales | 30.9700000 | 22.3300000 | 8.6410000 | 0.0552963 | 0.0290306 |
| unclassified | 0.0000000 | 0.0394200 | -0.0394200 | 0.0807714 | 0.0391431 |
| Selenomonadales | 0.0033550 | 0.0000000 | 0.0033550 | 0.0892770 | 0.0401747 |
| **Taxonomy** | **F** | **OVX+E2** | **Difference** | **P value** | **FDR value** |
| Desulfovibrionales | 7.5500000 | 0.0222300 | 7.5270000 | 0.0000000 | 0.0000004 |
| Bifidobacteriales | 0.3921000 | 2.2370000 | -1.8450000 | 0.0000183 | 0.0000808 |
| Clostridiales | 11.9200000 | 35.9500000 | -24.0300000 | 0.0000256 | 0.0000808 |
| Burkholderiales | 0.3200000 | 0.0182400 | 0.3018000 | 0.0047094 | 0.0091854 |
| Campylobacterales | 0.0067110 | 0.0000000 | 0.0067110 | 0.0053548 | 0.0091854 |
| Rhodospirillales | 0.7602000 | 0.0390100 | 0.7212000 | 0.0058320 | 0.0091854 |
| unclassified | 0.0000000 | 0.0337600 | -0.0337600 | 0.0080058 | 0.0108078 |
| Bdellovibrionales | 2.1330000 | 0.1269000 | 2.0060000 | 0.0276830 | 0.0327005 |
| Lactobacillales | 0.1560000 | 0.7403000 | -0.5842000 | 0.0371194 | 0.0389754 |
| **Taxonomy** | **M+E2** | **OVX** | **Difference** | **P value** | **FDR value** |
| Enterobacteriales | 0.0100700 | 34.4700000 | -34.4600000 | 0.0000000 | 0.0000000 |
| Bacteroidales | 36.4200000 | 15.1000000 | 21.3200000 | 0.0002442 | 0.0014104 |
| Selenomonadales | 0.0358600 | 0.0000000 | 0.0358600 | 0.0022341 | 0.0086013 |
| Deferribacterales | 9.3840000 | 4.1230000 | 5.2610000 | 0.0043330 | 0.0125114 |
| Lactobacillales | 0.3676000 | 4.6390000 | -4.2710000 | 0.0131784 | 0.0304420 |
| Coriobacteriales | 0.0088080 | 0.0000000 | 0.0088080 | 0.0202601 | 0.0390008 |
| **Taxonomy** | **M+E2** | **OVX+E2** | **Difference** | **P value** | **FDR value** |
| Bifidobacteriales | 0.0000000 | 2.2370000 | -2.2370000 | 0.0000032 | 0.0000466 |
| Selenomonadales | 0.0358600 | 0.0000000 | 0.0358600 | 0.0022341 | 0.0164207 |
| **Taxonomy** | **OVX** | **OVX+E2** | **Difference** | **P value** | **FDR value** |
| Enterobacteriales | 34.4700000 | 0.2439000 | 34.2300000 | 0.0000000 | 0.0000000 |
| Clostridiales | 19.0500000 | 35.9500000 | -16.9000000 | 0.0000019 | 0.0000101 |
| Bifidobacteriales | 0.0000000 | 2.2370000 | -2.2370000 | 0.0000032 | 0.0000111 |
| Coriobacteriales | 0.0000000 | 0.0067110 | -0.0067110 | 0.0053548 | 0.0140564 |
| Deferribacterales | 4.1230000 | 8.2490000 | -4.1260000 | 0.0147103 | 0.0308916 |
| Lactobacillales | 4.6390000 | 0.7403000 | 3.8990000 | 0.0206252 | 0.0360940 |
| Desulfovibrionales | 0.0067100 | 0.0222300 | -0.0155200 | 0.0267969 | 0.0401953 |

FAMILY

|  | Mean1 | Mean2 |  |  |  |
| --- | --- | --- | --- | --- | --- |
| **Taxonomy** | **Male (M)** | **Female (F)** | **Difference** | **P value** | **FDR value** |
| Desulfovibrionaceae | 0.000000 | 7.550000 | -7.550000 | 0.000000 | 0.000001 |
| Enterobacteriaceae | 22.610000 | 0.108200 | 22.500000 | 0.000000 | 0.000001 |
| Peptococcaceae_1 | 5.760000 | 0.117400 | 5.642000 | 0.000057 | 0.000338 |
| Victivallaceae | 0.074860 | 0.000000 | 0.074860 | 0.000119 | 0.000530 |
| Bifidobacteriaceae | 0.003355 | 0.392100 | -0.388800 | 0.000152 | 0.000543 |
| Verrucomicrobiaceae | 12.400000 | 30.970000 | -18.570000 | 0.000279 | 0.000831 |
| Sutterellaceae | 0.000000 | 0.320000 | -0.320000 | 0.003352 | 0.007496 |
| Cryomorphaceae | 0.265900 | 0.000000 | 0.265900 | 0.003359 | 0.007496 |
| Rhodospirillaceae | 0.000000 | 0.760200 | -0.760200 | 0.004325 | 0.008579 |
| Incertae_Sedis_XI | 0.006711 | 0.000000 | 0.006711 | 0.005355 | 0.009558 |
| Porphyromonadaceae | 24.420000 | 15.490000 | 8.924000 | 0.007562 | 0.012271 |
| Veillonellaceae | 0.025580 | 0.003355 | 0.022230 | 0.008836 | 0.013143 |
| Rikenellaceae | 1.749000 | 4.363000 | -2.614000 | 0.013632 | 0.018718 |
| Bacteroidaceae | 9.367000 | 20.510000 | -11.140000 | 0.015102 | 0.019255 |
| Ruminococcaceae | 5.541000 | 3.008000 | 2.534000 | 0.016279 | 0.019322 |
| Eubacteriaceae | 0.451900 | 1.686000 | -1.234000 | 0.017320 | 0.019322 |
| **Taxonomy** | **Male (M)** | **M+E2** | **Difference** | **P value** | **FDR value** |
| Enterobacteriaceae | 22.610000 | 0.010070 | 22.600000 | 0.000000 | 0.000002 |
| Defluviitaleaceae | 0.000000 | 0.012790 | -0.012790 | 0.000078 | 0.000740 |
| Victivallaceae | 0.074860 | 0.000000 | 0.074860 | 0.000119 | 0.000749 |
| Porphyromonadaceae | 24.420000 | 13.620000 | 10.790000 | 0.000449 | 0.002121 |
| Bacteroidaceae | 9.367000 | 19.560000 | -10.190000 | 0.002118 | 0.007860 |
| Eubacteriaceae | 0.451900 | 1.563000 | -1.111000 | 0.002940 | 0.007860 |
| Verrucomicrobiaceae | 12.400000 | 25.710000 | -13.310000 | 0.003043 | 0.007860 |
| Cryomorphaceae | 0.265900 | 0.000000 | 0.265900 | 0.003359 | 0.007860 |
| Rikenellaceae | 1.749000 | 3.238000 | -1.489000 | 0.003743 | 0.007860 |
| Incertae_Sedis_XI | 0.006711 | 0.000000 | 0.006711 | 0.005355 | 0.009201 |
| Helicobacteraceae | 0.006711 | 0.000000 | 0.006711 | 0.005355 | 0.009201 |
| Marinilabiliaceae | 0.000000 | 0.006082 | -0.006082 | 0.008033 | 0.011844 |
| Streptococcaceae | 0.049070 | 0.002726 | 0.046340 | 0.008147 | 0.011844 |
| Rhodospirillaceae | 0.000000 | 0.352700 | -0.352700 | 0.014661 | 0.019195 |
| Bdellovibrionaceae | 1.137000 | 0.043620 | 1.093000 | 0.015234 | 0.019195 |
| Desulfovibrionaceae | 0.000000 | 0.008808 | -0.008808 | 0.020260 | 0.023351 |
| Deferribacteraceae | 5.904000 | 9.384000 | -3.480000 | 0.022101 | 0.023351 |
| unclassified | 0.000000 | 0.297600 | -0.297600 | 0.022239 | 0.023351 |
| **Taxonomy** | **Male (M)** | **OVX** | **Difference** | **P value** | **FDR value** |
| Porphyromonadaceae | 24.420000 | 5.497000 | 18.920000 | 0.000010 | 0.000194 |
| Enterobacteriaceae | 22.610000 | 34.470000 | -11.860000 | 0.000060 | 0.000599 |
| Victivallaceae | 0.074860 | 0.000000 | 0.074860 | 0.000119 | 0.000790 |
| Sutterellaceae | 0.000000 | 0.030410 | -0.030410 | 0.001310 | 0.006534 |
| Cryomorphaceae | 0.265900 | 0.000000 | 0.265900 | 0.003359 | 0.011287 |
| Veillonellaceae | 0.025580 | 0.000000 | 0.025580 | 0.003395 | 0.011287 |
| Helicobacteraceae | 0.006711 | 0.000000 | 0.006711 | 0.005355 | 0.013354 |
| Incertae_Sedis_XI | 0.006711 | 0.000000 | 0.006711 | 0.005355 | 0.013354 |
| Lactobacillaceae | 0.166900 | 4.618000 | -4.451000 | 0.010478 | 0.023227 |
| Rikenellaceae | 1.749000 | 0.639400 | 1.109000 | 0.013871 | 0.024326 |
| Eubacteriaceae | 0.451900 | 0.069830 | 0.382100 | 0.014161 | 0.024326 |
| Peptococcaceae_1 | 5.760000 | 3.494000 | 2.266000 | 0.015283 | 0.024326 |
| Bdellovibrionaceae | 1.137000 | 0.053260 | 1.084000 | 0.015852 | 0.024326 |
| Deferribacteraceae | 5.904000 | 4.123000 | 1.781000 | 0.025648 | 0.036548 |
| **Taxonomy** | **Male (M)** | **OVX+E2** | **Difference** | **P value** | **FDR value** |
| Enterobacteriaceae | 22.610000 | 0.243900 | 22.360000 | 0.000000 | 0.000002 |
| Bifidobacteriaceae | 0.003355 | 2.237000 | -2.234000 | 0.000003 | 0.000024 |
| Lachnospiraceae | 9.497000 | 20.360000 | -10.860000 | 0.000003 | 0.000024 |
| Victivallaceae | 0.074860 | 0.000000 | 0.074860 | 0.000119 | 0.000624 |
| Ruminococcaceae | 5.541000 | 10.110000 | -4.568000 | 0.000601 | 0.002525 |
| Desulfovibrionaceae | 0.000000 | 0.022230 | -0.022230 | 0.001238 | 0.004333 |
| Porphyromonadaceae | 24.420000 | 11.780000 | 12.640000 | 0.001897 | 0.005690 |
| Cryomorphaceae | 0.265900 | 0.000000 | 0.265900 | 0.003359 | 0.007921 |
| Veillonellaceae | 0.025580 | 0.000000 | 0.025580 | 0.003395 | 0.007921 |
| Helicobacteraceae | 0.006711 | 0.000000 | 0.006711 | 0.005355 | 0.010223 |
| Incertae_Sedis_XI | 0.006711 | 0.000000 | 0.006711 | 0.005355 | 0.010223 |
| unclassified | 0.000000 | 0.033760 | -0.033760 | 0.008006 | 0.012049 |
| Prevotellaceae | 0.000000 | 0.006082 | -0.006082 | 0.008033 | 0.012049 |
| Sutterellaceae | 0.000000 | 0.018240 | -0.018240 | 0.008033 | 0.012049 |
| Rhodospirillaceae | 0.000000 | 0.039010 | -0.039010 | 0.017474 | 0.024464 |
| Bdellovibrionaceae | 1.137000 | 0.126900 | 1.010000 | 0.022087 | 0.028989 |
| Clostridiales_Incertae_Sedis_XII | 0.002726 | 0.020130 | -0.017410 | 0.025446 | 0.031434 |
| **Taxonomy** | **F** | **M+E2** | **Difference** | **P value** | **FDR value** |
| Desulfovibrionaceae | 7.550000 | 0.008808 | 7.541000 | 0.000000 | 0.000001 |
| Defluviitaleaceae | 0.000000 | 0.012790 | -0.012790 | 0.000078 | 0.000777 |
| Bifidobacteriaceae | 0.392100 | 0.000000 | 0.392100 | 0.000143 | 0.000777 |
| Peptococcaceae_1 | 0.117400 | 7.569000 | -7.451000 | 0.000148 | 0.000777 |
| Enterobacteriaceae | 0.108200 | 0.010070 | 0.098140 | 0.003222 | 0.013531 |
| Veillonellaceae | 0.003355 | 0.035860 | -0.032500 | 0.004446 | 0.015129 |
| Helicobacteraceae | 0.006711 | 0.000000 | 0.006711 | 0.005355 | 0.015129 |
| Ruminococcaceae | 3.008000 | 6.025000 | -3.018000 | 0.005763 | 0.015129 |
| Marinilabiliaceae | 0.000000 | 0.006082 | -0.006082 | 0.008033 | 0.018743 |
| Streptococcaceae | 0.033550 | 0.002726 | 0.030830 | 0.009232 | 0.019387 |
| Deferribacteraceae | 5.090000 | 9.384000 | -4.294000 | 0.020658 | 0.037527 |
| unclassified | 0.000000 | 0.297600 | -0.297600 | 0.022239 | 0.037527 |
| Bdellovibrionaceae | 2.133000 | 0.043620 | 2.089000 | 0.023231 | 0.037527 |
| **Taxonomy** | **F** | **OVX** | **Difference** | **P value** | **FDR value** |
| Enterobacteriaceae | 0.108200 | 34.470000 | -34.360000 | 0.000000 | 0.000000 |
| Peptococcaceae_1 | 0.117400 | 3.494000 | -3.377000 | 0.000000 | 0.000000 |
| Desulfovibrionaceae | 7.550000 | 0.006710 | 7.543000 | 0.000000 | 0.000000 |
| Bifidobacteriaceae | 0.392100 | 0.000000 | 0.392100 | 0.000143 | 0.000450 |
| Porphyromonadaceae | 15.490000 | 5.497000 | 9.994000 | 0.000488 | 0.001231 |
| Rikenellaceae | 4.363000 | 0.639400 | 3.723000 | 0.001395 | 0.002930 |
| Eubacteriaceae | 1.686000 | 0.069830 | 1.616000 | 0.003577 | 0.006439 |
| Helicobacteraceae | 0.006711 | 0.000000 | 0.006711 | 0.005355 | 0.007454 |
| Clostridiales_Incertae_Sedis_XIII | 0.000000 | 0.019500 | -0.019500 | 0.005660 | 0.007454 |
| Sutterellaceae | 0.320000 | 0.030410 | 0.289600 | 0.005916 | 0.007454 |
| Rhodospirillaceae | 0.760200 | 0.053680 | 0.706500 | 0.006741 | 0.007721 |
| Coriobacteriaceae | 0.006082 | 0.000000 | 0.006082 | 0.008033 | 0.008435 |
| Lactobacillaceae | 0.122500 | 4.618000 | -4.496000 | 0.009984 | 0.009676 |
| Bacteroidaceae | 20.510000 | 8.968000 | 11.540000 | 0.018106 | 0.015769 |
| Ruminococcaceae | 3.008000 | 5.497000 | -2.489000 | 0.018773 | 0.015769 |
| Bdellovibrionaceae | 2.133000 | 0.053260 | 2.080000 | 0.023691 | 0.018657 |
| Prevotellaceae | 0.068570 | 0.000000 | 0.068570 | 0.052802 | 0.038707 |
| Verrucomicrobiaceae | 30.970000 | 22.330000 | 8.641000 | 0.055296 | 0.038707 |
| **Taxonomy** | **F** | **OVX+E2** | **Difference** | **P value** | **FDR value** |
| Desulfovibrionaceae | 7.550000 | 0.022230 | 7.527000 | 0.000000 | 0.000001 |
| Peptococcaceae_1 | 0.117400 | 4.965000 | -4.847000 | 0.000009 | 0.000085 |
| Bifidobacteriaceae | 0.392100 | 2.237000 | -1.845000 | 0.000018 | 0.000115 |
| Ruminococcaceae | 3.008000 | 10.110000 | -7.101000 | 0.000042 | 0.000198 |
| Lachnospiraceae | 6.800000 | 20.360000 | -13.560000 | 0.000233 | 0.000883 |
| Sutterellaceae | 0.320000 | 0.018240 | 0.301800 | 0.004709 | 0.012247 |
| Helicobacteraceae | 0.006711 | 0.000000 | 0.006711 | 0.005355 | 0.012247 |
| Eubacteriaceae | 1.686000 | 0.204300 | 1.481000 | 0.005750 | 0.012247 |
| Rhodospirillaceae | 0.760200 | 0.039010 | 0.721200 | 0.005832 | 0.012247 |
| unclassified | 0.000000 | 0.033760 | -0.033760 | 0.008006 | 0.015131 |
| Bdellovibrionaceae | 2.133000 | 0.126900 | 2.006000 | 0.027683 | 0.046410 |
| Clostridiales_Incertae_Sedis_XII | 0.003355 | 0.020130 | -0.016780 | 0.029467 | 0.046410 |
| **Taxonomy** | **M+E2** | **OVX** | **Difference** | **P value** | **FDR value** |
| Enterobacteriaceae | 0.010070 | 34.470000 | -34.460000 | 0.000000 | 0.000000 |
| Porphyromonadaceae | 13.620000 | 5.497000 | 8.123000 | 0.000001 | 0.000009 |
| Rikenellaceae | 3.238000 | 0.639400 | 2.599000 | 0.000004 | 0.000021 |
| Defluviitaleaceae | 0.012790 | 0.000000 | 0.012790 | 0.000078 | 0.000329 |
| Eubacteriaceae | 1.563000 | 0.069830 | 1.493000 | 0.000242 | 0.000815 |
| Veillonellaceae | 0.035860 | 0.000000 | 0.035860 | 0.002234 | 0.006256 |
| Deferribacteraceae | 9.384000 | 4.123000 | 5.261000 | 0.004333 | 0.009408 |
| Bacteroidaceae | 19.560000 | 8.968000 | 10.590000 | 0.004480 | 0.009408 |
| Peptococcaceae_1 | 7.569000 | 3.494000 | 4.074000 | 0.006335 | 0.010956 |
| Streptococcaceae | 0.002726 | 0.020970 | -0.018240 | 0.006521 | 0.010956 |
| Marinilabiliaceae | 0.006082 | 0.000000 | 0.006082 | 0.008033 | 0.012268 |
| Lactobacillaceae | 0.364900 | 4.618000 | -4.253000 | 0.013599 | 0.019039 |
| Coriobacteriaceae | 0.008808 | 0.000000 | 0.008808 | 0.020260 | 0.026182 |
| Rhodospirillaceae | 0.352700 | 0.053680 | 0.299000 | 0.033552 | 0.040263 |
| unclassified | 0.297600 | 0.039420 | 0.258100 | 0.042455 | 0.047550 |
| **Taxonomy** | **M+E2** | **OVX+E2** | **Difference** | **P value** | **FDR value** |
| Bifidobacteriaceae | 0.000000 | 2.237000 | -2.237000 | 0.000003 | 0.000073 |
| Defluviitaleaceae | 0.012790 | 0.000000 | 0.012790 | 0.000078 | 0.000604 |
| Streptococcaceae | 0.002726 | 0.028310 | -0.025580 | 0.000078 | 0.000604 |
| Eubacteriaceae | 1.563000 | 0.204300 | 1.359000 | 0.000451 | 0.002603 |
| Ruminococcaceae | 6.025000 | 10.110000 | -4.084000 | 0.000991 | 0.004580 |
| Veillonellaceae | 0.035860 | 0.000000 | 0.035860 | 0.002234 | 0.008601 |
| Lachnospiraceae | 11.700000 | 20.360000 | -8.660000 | 0.006241 | 0.020596 |
| Marinilabiliaceae | 0.006082 | 0.000000 | 0.006082 | 0.008033 | 0.023195 |
| **Taxonomy** | **OVX** | **OVX+E2** | **Difference** | **P value** | **FDR value** |
| Enterobacteriaceae | 34.470000 | 0.243900 | 34.230000 | 0.000000 | 0.000000 |
| Bifidobacteriaceae | 0.000000 | 2.237000 | -2.237000 | 0.000003 | 0.000018 |
| Lachnospiraceae | 9.670000 | 20.360000 | -10.690000 | 0.000005 | 0.000018 |
| Ruminococcaceae | 5.497000 | 10.110000 | -4.612000 | 0.000617 | 0.001780 |
| Coriobacteriaceae | 0.000000 | 0.006711 | -0.006711 | 0.005355 | 0.012370 |
| Prevotellaceae | 0.000000 | 0.006082 | -0.006082 | 0.008033 | 0.015463 |
| Clostridiales_Incertae_Sedis_XII | 0.000000 | 0.020130 | -0.020130 | 0.010851 | 0.017904 |
| Deferribacteraceae | 4.123000 | 8.249000 | -4.126000 | 0.014710 | 0.018359 |
| Clostridiales_Incertae_Sedis_XIII | 0.019500 | 0.002726 | 0.016780 | 0.015822 | 0.018359 |
| Peptococcaceae_1 | 3.494000 | 4.965000 | -1.470000 | 0.018200 | 0.018359 |
| Porphyromonadaceae | 5.497000 | 11.780000 | -6.282000 | 0.018566 | 0.018359 |
| Eubacteriaceae | 0.069830 | 0.204300 | -0.134400 | 0.019467 | 0.018359 |
| Lactobacillaceae | 4.618000 | 0.711900 | 3.906000 | 0.020664 | 0.018359 |
| Desulfovibrionaceae | 0.006710 | 0.022230 | -0.015520 | 0.026797 | 0.020957 |
| Rikenellaceae | 0.639400 | 2.320000 | -1.681000 | 0.027217 | 0.020957 |
| Bdellovibrionaceae | 0.053260 | 0.126900 | -0.073610 | 0.056599 | 0.040857 |

GENUS

|  | Mean1 | Mean2 |  |  |  |
| --- | --- | --- | --- | --- | --- |
| **Taxonomy** | **Male (M)** | **Female (F)** | **Difference** | **P value** | **FDR value** |
| Bilophila | 0.0000000 | 7.5500000 | -7.5500000 | 0.0000000 | 0.000002 |
| Escherichia | 22.5900000 | 0.0033550 | 22.5800000 | 0.0000001 | 0.000002 |
| Anaerotruncus | 0.9388000 | 0.2133000 | 0.7256000 | 0.0000002 | 0.000003 |
| Butyricimonas | 8.4020000 | 0.0000000 | 8.4020000 | 0.0000015 | 0.000017 |
| Acetanaerobacterium | 0.1791000 | 0.0134200 | 0.1657000 | 0.0000094 | 0.000087 |
| Sporobacter | 0.0249500 | 0.0033550 | 0.0216000 | 0.0000219 | 0.000168 |
| Ruminococcus2 | 0.5647000 | 0.1399000 | 0.4249000 | 0.0000256 | 0.000169 |
| Peptococcus | 5.3940000 | 0.0033550 | 5.3910000 | 0.0000498 | 0.000288 |
| Victivallis | 0.0748600 | 0.0000000 | 0.0748600 | 0.0001189 | 0.000610 |
| Bifidobacterium | 0.0033550 | 0.3921000 | -0.3888000 | 0.0001520 | 0.000702 |
| Parasporobacterium | 0.1795000 | 0.0000000 | 0.1795000 | 0.0001839 | 0.000772 |
| Akkermansia | 12.4000000 | 30.9700000 | -18.5700000 | 0.0002793 | 0.001038 |
| Clostridium_IV | 2.1830000 | 0.5580000 | 1.6250000 | 0.0002922 | 0.001038 |
| Shuttleworthia | 1.0080000 | 0.0394200 | 0.9684000 | 0.0006140 | 0.002026 |
| Parasutterella | 0.0000000 | 0.3200000 | -0.3200000 | 0.0033522 | 0.009700 |
| Wandonia | 0.2659000 | 0.0000000 | 0.2659000 | 0.0033594 | 0.009700 |
| Insolitispirillum | 0.0000000 | 0.7602000 | -0.7602000 | 0.0043255 | 0.011380 |
| Clostridium_XlVa | 3.7530000 | 1.7130000 | 2.0400000 | 0.0044337 | 0.011380 |
| Clostridium_III | 0.0067110 | 0.0000000 | 0.0067110 | 0.0053548 | 0.011781 |
| Holdemania | 0.0067110 | 0.0000000 | 0.0067110 | 0.0053548 | 0.011781 |
| Dethiosulfatibacter | 0.0067110 | 0.0000000 | 0.0067110 | 0.0053548 | 0.011781 |
| Enterobacter | 0.0201300 | 0.1021000 | -0.0819900 | 0.0075800 | 0.015463 |
| Pseudobutyrivibrio | 0.2810000 | 0.0000000 | 0.2810000 | 0.0078908 | 0.015463 |
| Alkalibacter | 0.0060820 | 0.0000000 | 0.0060820 | 0.0080329 | 0.015463 |
| Anaerosinus | 0.0255800 | 0.0033550 | 0.0222300 | 0.0088359 | 0.016329 |
| Roseburia | 0.0689900 | 0.0088080 | 0.0601800 | 0.0125505 | 0.022301 |
| Coprobacillus | 0.0000000 | 0.0188700 | -0.0188700 | 0.0133501 | 0.022493 |
| Alistipes | 1.7490000 | 4.3630000 | -2.6140000 | 0.0136319 | 0.022493 |
| Bacteroides | 9.3670000 | 20.5100000 | -11.1400000 | 0.0151023 | 0.024060 |
| Eubacterium | 0.4458000 | 1.6860000 | -1.2400000 | 0.0169408 | 0.026089 |
| Desulfitibacter | 0.3655000 | 0.1141000 | 0.2514000 | 0.0205918 | 0.030688 |
| Coprococcus | 0.1386000 | 0.0687800 | 0.0698300 | 0.0218521 | 0.031549 |
| Subdoligranulum | 0.0000000 | 0.1533000 | -0.1533000 | 0.0309981 | 0.043397 |
| **Taxonomy** | **Male (M)** | **M+E2** | **Difference** | **P value** | **FDR value** |
| Anaerotruncus | 0.9388000 | 0.2321000 | 0.7067000 | 0.0000000 | 0.000000 |
| Escherichia | 22.5900000 | 0.0067100 | 22.5800000 | 0.0000001 | 0.000002 |
| Barnesiella | 0.0027260 | 8.6700000 | -8.6670000 | 0.0000002 | 0.000003 |
| Butyricimonas | 8.4020000 | 0.0000000 | 8.4020000 | 0.0000015 | 0.000019 |
| Acetanaerobacterium | 0.1791000 | 0.0134200 | 0.1657000 | 0.0000065 | 0.000061 |
| Ruminococcus2 | 0.5647000 | 0.2122000 | 0.3525000 | 0.0000071 | 0.000061 |
| Tannerella | 0.0000000 | 2.2160000 | -2.2160000 | 0.0000193 | 0.000142 |
| Acetivibrio | 0.0127900 | 0.2546000 | -0.2418000 | 0.0000756 | 0.000448 |
| Defluviitalea | 0.0000000 | 0.0127900 | -0.0127900 | 0.0000784 | 0.000448 |
| Victivallis | 0.0748600 | 0.0000000 | 0.0748600 | 0.0001189 | 0.000612 |
| Parabacteroides | 16.0100000 | 2.7350000 | 13.2700000 | 0.0001440 | 0.000674 |
| Bacteroides | 9.3670000 | 19.5600000 | -10.1900000 | 0.0021183 | 0.008411 |
| Clostridium_IV | 2.1830000 | 1.0250000 | 1.1570000 | 0.0021252 | 0.008411 |
| Dorea | 0.0306200 | 0.1462000 | -0.1155000 | 0.0023968 | 0.008808 |
| Shuttleworthia | 1.0080000 | 0.2049000 | 0.8030000 | 0.0026141 | 0.008967 |
| Eubacterium | 0.4458000 | 1.5600000 | -1.1140000 | 0.0028554 | 0.009080 |
| Akkermansia | 12.4000000 | 25.7100000 | -13.3100000 | 0.0030433 | 0.009080 |
| Pseudoflavonifractor | 0.6916000 | 2.2070000 | -1.5150000 | 0.0031765 | 0.009080 |
| Wandonia | 0.2659000 | 0.0000000 | 0.2659000 | 0.0033594 | 0.009097 |
| Coprococcus | 0.1386000 | 0.0425700 | 0.0960400 | 0.0036874 | 0.009170 |
| Alistipes | 1.7490000 | 3.2380000 | -1.4890000 | 0.0037429 | 0.009170 |
| Clostridium_III | 0.0067110 | 0.0000000 | 0.0067110 | 0.0053548 | 0.011020 |
| Dethiosulfatibacter | 0.0067110 | 0.0000000 | 0.0067110 | 0.0053548 | 0.011020 |
| Saccharofermentans | 0.0000000 | 0.0067110 | -0.0067110 | 0.0053548 | 0.011020 |
| Helicobacter | 0.0067110 | 0.0000000 | 0.0067110 | 0.0053548 | 0.011020 |
| Pseudobutyrivibrio | 0.2810000 | 0.0000000 | 0.2810000 | 0.0078908 | 0.014454 |
| Alkalitalea | 0.0000000 | 0.0060820 | -0.0060820 | 0.0080329 | 0.014454 |
| Alkalibacter | 0.0060820 | 0.0000000 | 0.0060820 | 0.0080329 | 0.014454 |
| Lactococcus | 0.0490700 | 0.0027260 | 0.0463400 | 0.0081469 | 0.014454 |
| Flavonifractor | 0.2242000 | 0.4641000 | -0.2399000 | 0.0097756 | 0.016765 |
| Insolitispirillum | 0.0000000 | 0.3527000 | -0.3527000 | 0.0146615 | 0.024333 |
| Vampirovibrio | 1.1370000 | 0.0436200 | 1.0930000 | 0.0152340 | 0.024493 |
| Mucispirillum | 5.9040000 | 9.3840000 | -3.4800000 | 0.0221011 | 0.033653 |
| unclassified | 0.0000000 | 0.2976000 | -0.2976000 | 0.0222391 | 0.033653 |
| Subdoligranulum | 0.0000000 | 0.0127900 | -0.0127900 | 0.0230793 | 0.033927 |
| Marvinbryantia | 2.3850000 | 6.0610000 | -3.6760000 | 0.0253505 | 0.036230 |
| Roseburia | 0.0689900 | 0.0188700 | 0.0501200 | 0.0284046 | 0.039498 |
| Enterobacter | 0.0201300 | 0.0000000 | 0.0201300 | 0.0359174 | 0.048630 |
| **Taxonomy** | **Male (M)** | **OVX** | **Difference** | **P value** | **FDR value** |
| Butyricimonas | 8.4020000 | 0.0000000 | 8.4020000 | 0.0000015 | 0.000065 |
| Tannerella | 0.0000000 | 0.8233000 | -0.8233000 | 0.0000048 | 0.000078 |
| Acetanaerobacterium | 0.1791000 | 0.0155200 | 0.1636000 | 0.0000054 | 0.000078 |
| Barnesiella | 0.0027260 | 3.3600000 | -3.3570000 | 0.0000133 | 0.000143 |
| Anaerotruncus | 0.9388000 | 0.4509000 | 0.4880000 | 0.0000215 | 0.000185 |
| Ruminococcus2 | 0.5647000 | 0.1894000 | 0.3754000 | 0.0000406 | 0.000292 |
| Escherichia | 22.5900000 | 34.4700000 | -11.8800000 | 0.0000607 | 0.000373 |
| Parabacteroides | 16.0100000 | 1.3140000 | 14.7000000 | 0.0000702 | 0.000375 |
| Allobaculum | 0.0127900 | 0.0000000 | 0.0127900 | 0.0000784 | 0.000375 |
| Victivallis | 0.0748600 | 0.0000000 | 0.0748600 | 0.0001189 | 0.000512 |
| Dorea | 0.0306200 | 0.4249000 | -0.3942000 | 0.0003517 | 0.001377 |
| Parasutterella | 0.0000000 | 0.0304100 | -0.0304100 | 0.0013101 | 0.004700 |
| Shuttleworthia | 1.0080000 | 0.2336000 | 0.7742000 | 0.0025465 | 0.008433 |
| Wandonia | 0.2659000 | 0.0000000 | 0.2659000 | 0.0033594 | 0.009743 |
| Anaerosinus | 0.0255800 | 0.0000000 | 0.0255800 | 0.0033947 | 0.009743 |
| Hydrogenoanaerobacterium | 0.3970000 | 0.0910100 | 0.3060000 | 0.0045372 | 0.011270 |
| Holdemania | 0.0067110 | 0.0000000 | 0.0067110 | 0.0053548 | 0.011270 |
| Clostridium_III | 0.0067110 | 0.0000000 | 0.0067110 | 0.0053548 | 0.011270 |
| Dethiosulfatibacter | 0.0067110 | 0.0000000 | 0.0067110 | 0.0053548 | 0.011270 |
| Helicobacter | 0.0067110 | 0.0000000 | 0.0067110 | 0.0053548 | 0.011270 |
| Robinsoniella | 0.0603900 | 0.1537000 | -0.0933200 | 0.0054975 | 0.011270 |
| Roseburia | 0.0689900 | 0.0000000 | 0.0689900 | 0.0058596 | 0.011466 |
| Clostridium_IV | 2.1830000 | 0.9510000 | 1.2320000 | 0.0073321 | 0.013724 |
| Pseudobutyrivibrio | 0.2810000 | 0.0000000 | 0.2810000 | 0.0078908 | 0.013833 |
| Alkalibacter | 0.0060820 | 0.0000000 | 0.0060820 | 0.0080329 | 0.013833 |
| Oscillibacter | 0.8600000 | 2.6280000 | -1.7680000 | 0.0086632 | 0.014344 |
| Lactobacillus | 0.1669000 | 4.6180000 | -4.4510000 | 0.0104783 | 0.016707 |
| Alistipes | 1.7490000 | 0.6394000 | 1.1090000 | 0.0138711 | 0.020977 |
| Parasporobacterium | 0.1795000 | 0.0922700 | 0.0872400 | 0.0141310 | 0.020977 |
| Peptococcus | 5.3940000 | 3.2310000 | 2.1630000 | 0.0151597 | 0.021289 |
| Eubacterium | 0.4458000 | 0.0698300 | 0.3760000 | 0.0153299 | 0.021289 |
| Vampirovibrio | 1.1370000 | 0.0532600 | 1.0840000 | 0.0158516 | 0.021325 |
| Acetivibrio | 0.0127900 | 0.0000000 | 0.0127900 | 0.0230793 | 0.029222 |
| Papillibacter | 0.0000000 | 0.0127900 | -0.0127900 | 0.0230793 | 0.029222 |
| Mucispirillum | 5.9040000 | 4.1230000 | 1.7810000 | 0.0256477 | 0.031547 |
| Enterobacter | 0.0201300 | 0.0000000 | 0.0201300 | 0.0359174 | 0.042951 |
| Moryella | 0.0813700 | 0.0054520 | 0.0759100 | 0.0390676 | 0.045356 |
| Akkermansia | 12.4000000 | 22.3300000 | -9.9310000 | 0.0400357 | 0.045356 |
| **Taxonomy** | **Male (M)** | **OVX+E2** | **Difference** | **P value** | **FDR value** |
| Escherichia | 22.5900000 | 0.2439000 | 22.3400000 | 0.0000001 | 0.000004 |
| Dorea | 0.0306200 | 1.0500000 | -1.0200000 | 0.0000002 | 0.000004 |
| Anaerotruncus | 0.9388000 | 0.3869000 | 0.5519000 | 0.0000015 | 0.000017 |
| Butyricimonas | 8.4020000 | 0.0000000 | 8.4020000 | 0.0000015 | 0.000017 |
| Marvinbryantia | 2.3850000 | 10.5200000 | -8.1370000 | 0.0000024 | 0.000022 |
| Bifidobacterium | 0.0033550 | 2.2370000 | -2.2340000 | 0.0000032 | 0.000025 |
| Acetanaerobacterium | 0.1791000 | 0.0283100 | 0.1508000 | 0.0000160 | 0.000103 |
| Pseudoflavonifractor | 0.6916000 | 2.7360000 | -2.0450000 | 0.0000179 | 0.000103 |
| Allobaculum | 0.0127900 | 0.0000000 | 0.0127900 | 0.0000784 | 0.000402 |
| Victivallis | 0.0748600 | 0.0000000 | 0.0748600 | 0.0001189 | 0.000549 |
| Parabacteroides | 16.0100000 | 2.4550000 | 13.5600000 | 0.0001467 | 0.000616 |
| Bilophila | 0.0000000 | 0.0188700 | -0.0188700 | 0.0002267 | 0.000873 |
| Barnesiella | 0.0027260 | 7.6900000 | -7.6880000 | 0.0002705 | 0.000961 |
| Clostridium_XlVa | 3.7530000 | 5.8170000 | -2.0640000 | 0.0003596 | 0.001159 |
| Flavonifractor | 0.2242000 | 1.4720000 | -1.2480000 | 0.0003762 | 0.001159 |
| Tannerella | 0.0000000 | 1.6340000 | -1.6340000 | 0.0007571 | 0.002186 |
| Robinsoniella | 0.0603900 | 0.3942000 | -0.3338000 | 0.0011173 | 0.003037 |
| Clostridium_XlVb | 0.1468000 | 0.0761200 | 0.0706700 | 0.0014478 | 0.003716 |
| Oscillibacter | 0.8600000 | 3.0500000 | -2.1900000 | 0.0019675 | 0.004784 |
| Sporobacter | 0.0249500 | 0.0100700 | 0.0148900 | 0.0028257 | 0.006527 |
| Wandonia | 0.2659000 | 0.0000000 | 0.2659000 | 0.0033594 | 0.007129 |
| Anaerosinus | 0.0255800 | 0.0000000 | 0.0255800 | 0.0033947 | 0.007129 |
| Clostridium_III | 0.0067110 | 0.0000000 | 0.0067110 | 0.0053548 | 0.009896 |
| Dethiosulfatibacter | 0.0067110 | 0.0000000 | 0.0067110 | 0.0053548 | 0.009896 |
| Helicobacter | 0.0067110 | 0.0000000 | 0.0067110 | 0.0053548 | 0.009896 |
| Roseburia | 0.0689900 | 0.0033550 | 0.0656400 | 0.0077888 | 0.012371 |
| Pseudobutyrivibrio | 0.2810000 | 0.0000000 | 0.2810000 | 0.0078908 | 0.012371 |
| unclassified | 0.0000000 | 0.0337600 | -0.0337600 | 0.0080058 | 0.012371 |
| Prevotella | 0.0000000 | 0.0060820 | -0.0060820 | 0.0080329 | 0.012371 |
| Parasutterella | 0.0000000 | 0.0182400 | -0.0182400 | 0.0080329 | 0.012371 |
| Shuttleworthia | 1.0080000 | 0.2940000 | 0.7138000 | 0.0093417 | 0.013922 |
| Butyricicoccus | 0.0033550 | 0.0161500 | -0.0127900 | 0.0109216 | 0.015768 |
| Insolitispirillum | 0.0000000 | 0.0390100 | -0.0390100 | 0.0174743 | 0.024464 |
| Vampirovibrio | 1.1370000 | 0.1269000 | 1.0100000 | 0.0220871 | 0.030012 |
| Desulfitibacter | 0.3655000 | 0.6461000 | -0.2806000 | 0.0229134 | 0.030246 |
| Acetivibrio | 0.0127900 | 0.0723500 | -0.0595600 | 0.0248387 | 0.031774 |
| Acidaminobacter | 0.0027260 | 0.0201300 | -0.0174100 | 0.0254465 | 0.031774 |
| Enterobacter | 0.0201300 | 0.0000000 | 0.0201300 | 0.0359174 | 0.043668 |
| **Taxonomy** | **F** | **M+E2** | **Difference** | **P value** | **FDR value** |
| Bilophila | 7.5500000 | 0.0054520 | 7.5440000 | 0.0000000 | 0.000002 |
| Barnesiella | 0.0094370 | 8.6700000 | -8.6600000 | 0.0000002 | 0.000005 |
| Tannerella | 0.0000000 | 2.2160000 | -2.2160000 | 0.0000193 | 0.000298 |
| Sporobacter | 0.0033550 | 0.0249500 | -0.0216000 | 0.0000219 | 0.000298 |
| Parasporobacterium | 0.0000000 | 0.1682000 | -0.1682000 | 0.0000415 | 0.000454 |
| Acetivibrio | 0.0100700 | 0.2546000 | -0.2445000 | 0.0000672 | 0.000535 |
| Parabacteroides | 15.4800000 | 2.7350000 | 12.7400000 | 0.0000774 | 0.000535 |
| Defluviitalea | 0.0000000 | 0.0127900 | -0.0127900 | 0.0000784 | 0.000535 |
| Peptococcus | 0.0033550 | 6.9250000 | -6.9220000 | 0.0001282 | 0.000778 |
| Bifidobacterium | 0.3921000 | 0.0000000 | 0.3921000 | 0.0001428 | 0.000779 |
| Dorea | 0.0337600 | 0.1462000 | -0.1124000 | 0.0010667 | 0.005295 |
| Enterobacter | 0.1021000 | 0.0000000 | 0.1021000 | 0.0015349 | 0.006984 |
| Clostridium_IV | 0.5580000 | 1.0250000 | -0.4674000 | 0.0018203 | 0.007645 |
| Desulfitibacter | 0.1141000 | 0.6432000 | -0.5291000 | 0.0027449 | 0.010705 |
| Anaerosinus | 0.0033550 | 0.0358600 | -0.0325000 | 0.0044459 | 0.016183 |
| Saccharofermentans | 0.0000000 | 0.0067110 | -0.0067110 | 0.0053548 | 0.017198 |
| Helicobacter | 0.0067110 | 0.0000000 | 0.0067110 | 0.0053548 | 0.017198 |
| Alkalitalea | 0.0000000 | 0.0060820 | -0.0060820 | 0.0080329 | 0.024367 |
| Lactococcus | 0.0335500 | 0.0027260 | 0.0308300 | 0.0092319 | 0.026530 |
| Oscillibacter | 0.5182000 | 1.3220000 | -0.8038000 | 0.0113334 | 0.030940 |
| Coprobacillus | 0.0188700 | 0.0000000 | 0.0188700 | 0.0133501 | 0.034710 |
| Pseudoflavonifractor | 0.9068000 | 2.2070000 | -1.3000000 | 0.0182104 | 0.045118 |
| Clostridium_XlVa | 1.7130000 | 4.2240000 | -2.5110000 | 0.0199533 | 0.045118 |
| Lactonifactor | 0.0000000 | 0.0088080 | -0.0088080 | 0.0202601 | 0.045118 |
| Mucispirillum | 5.0900000 | 9.3840000 | -4.2940000 | 0.0206582 | 0.045118 |
| unclassified | 0.0000000 | 0.2976000 | -0.2976000 | 0.0222391 | 0.046702 |
| Vampirovibrio | 2.1330000 | 0.0436200 | 2.0890000 | 0.0232308 | 0.046978 |
| **Taxonomy** | **F** | **OVX** | **Difference** | **P value** | **FDR value** |
| Escherichia | 0.0033550 | 34.4700000 | -34.4700000 | 0.0000000 | 0.000000 |
| Peptococcus | 0.0033550 | 3.2310000 | -3.2280000 | 0.0000000 | 0.000000 |
| Bilophila | 7.5500000 | 0.0067100 | 7.5430000 | 0.0000000 | 0.000000 |
| Parasporobacterium | 0.0000000 | 0.0922700 | -0.0922700 | 0.0000000 | 0.000000 |
| Tannerella | 0.0000000 | 0.8233000 | -0.8233000 | 0.0000048 | 0.000040 |
| Barnesiella | 0.0094370 | 3.3600000 | -3.3500000 | 0.0000135 | 0.000092 |
| Parabacteroides | 15.4800000 | 1.3140000 | 14.1600000 | 0.0000361 | 0.000211 |
| Bifidobacterium | 0.3921000 | 0.0000000 | 0.3921000 | 0.0001428 | 0.000731 |
| Dorea | 0.0337600 | 0.4249000 | -0.3911000 | 0.0003197 | 0.001455 |
| Shuttleworthia | 0.0394200 | 0.2336000 | -0.1942000 | 0.0005660 | 0.002318 |
| Alistipes | 4.3630000 | 0.6394000 | 3.7230000 | 0.0013951 | 0.005194 |
| Enterobacter | 0.1021000 | 0.0000000 | 0.1021000 | 0.0015349 | 0.005238 |
| Sporobacter | 0.0033550 | 0.0249500 | -0.0216000 | 0.0022140 | 0.006974 |
| Eubacterium | 1.6860000 | 0.0698300 | 1.6160000 | 0.0035770 | 0.010031 |
| Oscillibacter | 0.5182000 | 2.6280000 | -2.1100000 | 0.0036742 | 0.010031 |
| Helicobacter | 0.0067110 | 0.0000000 | 0.0067110 | 0.0053548 | 0.013458 |
| Anaerovorax | 0.0000000 | 0.0195000 | -0.0195000 | 0.0056599 | 0.013458 |
| Parasutterella | 0.3200000 | 0.0304100 | 0.2896000 | 0.0059158 | 0.013458 |
| Insolitispirillum | 0.7602000 | 0.0536800 | 0.7065000 | 0.0067406 | 0.014528 |
| Enterorhabdus | 0.0060820 | 0.0000000 | 0.0060820 | 0.0080329 | 0.016447 |
| Anaerotruncus | 0.2133000 | 0.4509000 | -0.2376000 | 0.0097704 | 0.018583 |
| Lactobacillus | 0.1225000 | 4.6180000 | -4.4960000 | 0.0099836 | 0.018583 |
| Acetivibrio | 0.0100700 | 0.0000000 | 0.0100700 | 0.0108487 | 0.019315 |
| Coprobacillus | 0.0188700 | 0.0000000 | 0.0188700 | 0.0133501 | 0.022779 |
| Bacteroides | 20.5100000 | 8.9650000 | 11.5400000 | 0.0180732 | 0.029604 |
| Roseburia | 0.0088080 | 0.0000000 | 0.0088080 | 0.0202601 | 0.031910 |
| Syntrophococcus | 0.1678000 | 1.2570000 | -1.0890000 | 0.0214871 | 0.032589 |
| Papillibacter | 0.0000000 | 0.0127900 | -0.0127900 | 0.0230793 | 0.033454 |
| Vampirovibrio | 2.1330000 | 0.0532600 | 2.0800000 | 0.0236912 | 0.033454 |
| Subdoligranulum | 0.1533000 | 0.0033550 | 0.1499000 | 0.0339398 | 0.046328 |
| **Taxonomy** | **F** | **OVX+E2** | **Difference** | **P value** | **FDR value** |
| Bilophila | 7.5500000 | 0.0188700 | 7.5310000 | 0.0000000 | 0.000002 |
| Dorea | 0.0337600 | 1.0500000 | -1.0170000 | 0.0000002 | 0.000004 |
| Bifidobacterium | 0.3921000 | 2.2370000 | -1.8450000 | 0.0000183 | 0.000239 |
| Peptococcus | 0.0033550 | 4.3190000 | -4.3150000 | 0.0000211 | 0.000239 |
| Desulfitibacter | 0.1141000 | 0.6461000 | -0.5320000 | 0.0000404 | 0.000364 |
| Parabacteroides | 15.4800000 | 2.4550000 | 13.0200000 | 0.0000823 | 0.000619 |
| Clostridium_XlVa | 1.7130000 | 5.8170000 | -4.1040000 | 0.0001382 | 0.000792 |
| Ruminococcus2 | 0.1399000 | 0.6597000 | -0.5199000 | 0.0001403 | 0.000792 |
| Barnesiella | 0.0094370 | 7.6900000 | -7.6810000 | 0.0002721 | 0.001365 |
| Johnsonella | 0.0000000 | 0.0350200 | -0.0350200 | 0.0004958 | 0.002212 |
| Clostridium_IV | 0.5580000 | 1.8530000 | -1.2940000 | 0.0005438 | 0.002212 |
| Pseudoflavonifractor | 0.9068000 | 2.7360000 | -1.8300000 | 0.0005879 | 0.002212 |
| Tannerella | 0.0000000 | 1.6340000 | -1.6340000 | 0.0007571 | 0.002630 |
| Oscillibacter | 0.5182000 | 3.0500000 | -2.5320000 | 0.0009049 | 0.002918 |
| Enterobacter | 0.1021000 | 0.0000000 | 0.1021000 | 0.0015349 | 0.004620 |
| Flavonifractor | 0.3592000 | 1.4720000 | -1.1130000 | 0.0017438 | 0.004921 |
| Marvinbryantia | 4.2710000 | 10.5200000 | -6.2500000 | 0.0023081 | 0.006130 |
| Parasutterella | 0.3200000 | 0.0182400 | 0.3018000 | 0.0047094 | 0.011813 |
| Helicobacter | 0.0067110 | 0.0000000 | 0.0067110 | 0.0053548 | 0.012539 |
| Eubacterium | 1.6860000 | 0.2015000 | 1.4840000 | 0.0056940 | 0.012539 |
| Insolitispirillum | 0.7602000 | 0.0390100 | 0.7212000 | 0.0058320 | 0.012539 |
| unclassified | 0.0000000 | 0.0337600 | -0.0337600 | 0.0080058 | 0.015769 |
| Prevotella | 0.0000000 | 0.0060820 | -0.0060820 | 0.0080329 | 0.015769 |
| Syntrophococcus | 0.1678000 | 0.8237000 | -0.6560000 | 0.0093345 | 0.017561 |
| Coprobacillus | 0.0188700 | 0.0000000 | 0.0188700 | 0.0133501 | 0.024110 |
| Coprococcus | 0.0687800 | 0.1919000 | -0.1231000 | 0.0163350 | 0.028366 |
| Acetivibrio | 0.0100700 | 0.0723500 | -0.0622800 | 0.0193482 | 0.032354 |
| Holdemania | 0.0000000 | 0.0088080 | -0.0088080 | 0.0202601 | 0.032669 |
| Parasporobacterium | 0.0000000 | 0.1260000 | -0.1260000 | 0.0215418 | 0.033538 |
| Anaerotruncus | 0.2133000 | 0.3869000 | -0.1736000 | 0.0229391 | 0.034523 |
| Robinsoniella | 0.1342000 | 0.3942000 | -0.2600000 | 0.0241558 | 0.035182 |
| Vampirovibrio | 2.1330000 | 0.1269000 | 2.0060000 | 0.0276830 | 0.039059 |
| Acidaminobacter | 0.0033550 | 0.0201300 | -0.0167800 | 0.0294666 | 0.040316 |
| Subdoligranulum | 0.1533000 | 0.0000000 | 0.1533000 | 0.0309981 | 0.041164 |
| Lactobacillus | 0.1225000 | 0.7119000 | -0.5895000 | 0.0347120 | 0.044778 |
| **Taxonomy** | **M+E2** | **OVX** | **Difference** | **P value** | **FDR value** |
| Escherichia | 0.0067100 | 34.4700000 | -34.4600000 | 0.0000000 | 0.000000 |
| Alistipes | 3.2380000 | 0.6394000 | 2.5990000 | 0.0000037 | 0.000089 |
| Hydrogenoanaerobacterium | 0.4521000 | 0.0910100 | 0.3611000 | 0.0000057 | 0.000091 |
| Barnesiella | 8.6700000 | 3.3600000 | 5.3100000 | 0.0000317 | 0.000383 |
| Acetivibrio | 0.2546000 | 0.0000000 | 0.2546000 | 0.0000488 | 0.000472 |
| Defluviitalea | 0.0127900 | 0.0000000 | 0.0127900 | 0.0000784 | 0.000631 |
| Roseburia | 0.0188700 | 0.0000000 | 0.0188700 | 0.0002267 | 0.001452 |
| Eubacterium | 1.5600000 | 0.0698300 | 1.4900000 | 0.0002405 | 0.001452 |
| Tannerella | 2.2160000 | 0.8233000 | 1.3920000 | 0.0006663 | 0.003576 |
| Anaerosinus | 0.0358600 | 0.0000000 | 0.0358600 | 0.0022341 | 0.010791 |
| Dorea | 0.1462000 | 0.4249000 | -0.2787000 | 0.0033039 | 0.014507 |
| Mucispirillum | 9.3840000 | 4.1230000 | 5.2610000 | 0.0043330 | 0.016590 |
| Bacteroides | 19.5600000 | 8.9650000 | 10.5900000 | 0.0044652 | 0.016590 |
| Anaerotruncus | 0.2321000 | 0.4509000 | -0.2187000 | 0.0051534 | 0.017779 |
| Lactococcus | 0.0027260 | 0.0209700 | -0.0182400 | 0.0065212 | 0.019089 |
| Parabacteroides | 2.7350000 | 1.3140000 | 1.4210000 | 0.0067003 | 0.019089 |
| Peptococcus | 6.9250000 | 3.2310000 | 3.6940000 | 0.0067186 | 0.019089 |
| Parasporobacterium | 0.1682000 | 0.0922700 | 0.0759100 | 0.0075295 | 0.020204 |
| Alkalitalea | 0.0060820 | 0.0000000 | 0.0060820 | 0.0080329 | 0.020421 |
| Flavonifractor | 0.4641000 | 0.2055000 | 0.2586000 | 0.0086628 | 0.020921 |
| Clostridium_XVIII | 0.0507500 | 0.0977200 | -0.0469700 | 0.0117586 | 0.027045 |
| Syntrophococcus | 0.0576700 | 1.2570000 | -1.1990000 | 0.0135031 | 0.028559 |
| Lactobacillus | 0.3649000 | 4.6180000 | -4.2530000 | 0.0135995 | 0.028559 |
| Lactonifactor | 0.0088080 | 0.0000000 | 0.0088080 | 0.0202601 | 0.039143 |
| Enterorhabdus | 0.0088080 | 0.0000000 | 0.0088080 | 0.0202601 | 0.039143 |
| Desulfitibacter | 0.6432000 | 0.2632000 | 0.3800000 | 0.0228586 | 0.042464 |
| Pseudoflavonifractor | 2.2070000 | 1.1080000 | 1.0990000 | 0.0260003 | 0.046512 |
| Oscillibacter | 1.3220000 | 2.6280000 | -1.3060000 | 0.0275601 | 0.047541 |
| **Taxonomy** | **M+E2** | **OVX+E2** | **Difference** | **P value** | **FDR value** |
| Dorea | 0.1462000 | 1.0500000 | -0.9042000 | 0.0000005 | 0.000030 |
| Bifidobacterium | 0.0000000 | 2.2370000 | -2.2370000 | 0.0000032 | 0.000087 |
| Lactococcus | 0.0027260 | 0.0249500 | -0.0222300 | 0.0000192 | 0.000349 |
| Defluviitalea | 0.0127900 | 0.0000000 | 0.0127900 | 0.0000784 | 0.001070 |
| Ruminococcus2 | 0.2122000 | 0.6597000 | -0.4475000 | 0.0001731 | 0.001890 |
| Eubacterium | 1.5600000 | 0.2015000 | 1.3580000 | 0.0004421 | 0.004023 |
| Johnsonella | 0.0027260 | 0.0350200 | -0.0322900 | 0.0010681 | 0.008331 |
| Acetivibrio | 0.2546000 | 0.0723500 | 0.1822000 | 0.0015028 | 0.010256 |
| Flavonifractor | 0.4641000 | 1.4720000 | -1.0080000 | 0.0016981 | 0.010302 |
| Roseburia | 0.0188700 | 0.0033550 | 0.0155200 | 0.0020150 | 0.011002 |
| Anaerosinus | 0.0358600 | 0.0000000 | 0.0358600 | 0.0022341 | 0.011089 |
| Sporobacter | 0.0249500 | 0.0100700 | 0.0148900 | 0.0028257 | 0.012857 |
| Syntrophococcus | 0.0576700 | 0.8237000 | -0.7660000 | 0.0036413 | 0.015293 |
| Saccharofermentans | 0.0067110 | 0.0000000 | 0.0067110 | 0.0053548 | 0.019669 |
| Oscillibacter | 1.3220000 | 3.0500000 | -1.7280000 | 0.0054036 | 0.019669 |
| Coprococcus | 0.0425700 | 0.1919000 | -0.1493000 | 0.0058526 | 0.019972 |
| Clostridium_IV | 1.0250000 | 1.8530000 | -0.8271000 | 0.0063260 | 0.020318 |
| Alkalitalea | 0.0060820 | 0.0000000 | 0.0060820 | 0.0080329 | 0.024367 |
| Anaerotruncus | 0.2321000 | 0.3869000 | -0.1548000 | 0.0102841 | 0.029553 |
| Butyricicoccus | 0.0033550 | 0.0161500 | -0.0127900 | 0.0109216 | 0.029816 |
| Marvinbryantia | 6.0610000 | 10.5200000 | -4.4610000 | 0.0167789 | 0.043625 |
| Moryella | 0.0142600 | 0.0383800 | -0.0241200 | 0.0190683 | 0.046092 |
| Bilophila | 0.0054520 | 0.0188700 | -0.0134200 | 0.0200232 | 0.046092 |
| Lactonifactor | 0.0088080 | 0.0000000 | 0.0088080 | 0.0202601 | 0.046092 |
| **Taxonomy** | **OVX** | **OVX+E2** | **Difference** | **P value** | **FDR value** |
| Escherichia | 34.4700000 | 0.2439000 | 34.2300000 | 0.0000000 | 0.000000 |
| Bifidobacterium | 0.0000000 | 2.2370000 | -2.2370000 | 0.0000032 | 0.000067 |
| Marvinbryantia | 3.8420000 | 10.5200000 | -6.6800000 | 0.0000789 | 0.000837 |
| Hydrogenoanaerobacterium | 0.0910100 | 0.4792000 | -0.3882000 | 0.0000882 | 0.000837 |
| Dorea | 0.4249000 | 1.0500000 | -0.6255000 | 0.0000996 | 0.000837 |
| Ruminococcus2 | 0.1894000 | 0.6597000 | -0.4704000 | 0.0002362 | 0.001653 |
| Flavonifractor | 0.2055000 | 1.4720000 | -1.2660000 | 0.0003546 | 0.002128 |
| Pseudoflavonifractor | 1.1080000 | 2.7360000 | -1.6290000 | 0.0004393 | 0.002306 |
| Johnsonella | 0.0000000 | 0.0350200 | -0.0350200 | 0.0004958 | 0.002314 |
| Moryella | 0.0054520 | 0.0383800 | -0.0329200 | 0.0007966 | 0.003346 |
| Clostridium_XlVa | 3.1280000 | 5.8170000 | -2.6890000 | 0.0011481 | 0.004384 |
| Butyricicoccus | 0.0000000 | 0.0161500 | -0.0161500 | 0.0016407 | 0.005742 |
| Desulfitibacter | 0.2632000 | 0.6461000 | -0.3829000 | 0.0019894 | 0.006427 |
| Robinsoniella | 0.1537000 | 0.3942000 | -0.2405000 | 0.0052403 | 0.014993 |
| Enterorhabdus | 0.0000000 | 0.0067110 | -0.0067110 | 0.0053548 | 0.014993 |
| Prevotella | 0.0000000 | 0.0060820 | -0.0060820 | 0.0080329 | 0.021086 |
| Acetivibrio | 0.0000000 | 0.0723500 | -0.0723500 | 0.0090234 | 0.022293 |
| Barnesiella | 3.3600000 | 7.6900000 | -4.3300000 | 0.0102721 | 0.023968 |
| Acidaminobacter | 0.0000000 | 0.0201300 | -0.0201300 | 0.0108507 | 0.023986 |
| Clostridium_XlVb | 0.1919000 | 0.0761200 | 0.1158000 | 0.0133529 | 0.028041 |
| Mucispirillum | 4.1230000 | 8.2490000 | -4.1260000 | 0.0147103 | 0.029421 |
| Anaerovorax | 0.0195000 | 0.0027260 | 0.0167800 | 0.0158223 | 0.030206 |
| Holdemania | 0.0000000 | 0.0088080 | -0.0088080 | 0.0202601 | 0.035809 |
| Lactobacillus | 4.6180000 | 0.7119000 | 3.9060000 | 0.0206636 | 0.035809 |
| Eubacterium | 0.0698300 | 0.2015000 | -0.1317000 | 0.0213152 | 0.035809 |
| Clostridium_IV | 0.9510000 | 1.8530000 | -0.9015000 | 0.0227053 | 0.036678 |
| Sporobacter | 0.0249500 | 0.0100700 | 0.0148900 | 0.0265428 | 0.040825 |
| Alistipes | 0.6394000 | 2.3200000 | -1.6810000 | 0.0272169 | 0.040825 |
| Bilophila | 0.0067100 | 0.0188700 | -0.0121600 | 0.0289365 | 0.041908 |
| Tannerella | 0.8233000 | 1.6340000 | -0.8111000 | 0.0347866 | 0.048701 |

M, male; F, female; E2, 17β-estradiol; OVX, ovariectomized; n=5/ group. Pair wise differential abundance analysis (Wilcoxon signed-rank test with false discovery rate (FDR) corrected p<0.05 for multiple comparisons) between groups was conducted using relative abundance (RA) calculated from OTUs table at the taxonomic levels of phylum, class, order, family and genus. Table shows the mean RA for each group and difference between two groups’ means. Taxa with FDR corrected p value of >0.05 are considered non significant.

**Table S**4. Primer sets used for real-time quantitative PCR

| **Gene** | **Forward primer (Sequence 5′- 3′)** | **Reverse primer (Sequence 5′- 3′)** |
| --- | --- | --- |
| **AKP3** | ACATTGCTACACAACTCATCTCC | TCCTGCCATCCAATCTGGTTC |
| **AKP6** | AGGATCCATCTGTCCTTTGGT | CAGCTGCCTTCTTGTTCC |
| **KLF4** | CCTATACGAAGAGTTCTCATCTCA | GTAGTGCCTGGTCAGTTCA |
| **CDX1** | CTGGCTGCTAACTTGGGTCTC | CTGCTGCTGCTGCTGCTG |
| **ER-α** | GGCACGACATTCTTGCATTTC | CTGGCCCAGCTCCTCCTC |
| **ER-β** | CCCACCATTAGCACCTCCAT | GATGATGTCCCTCACTAAGCTGG |
| **All bacteria (16s)** | ACTCCTACGGGAGGCAGCAGT | ATTACCGCGGCTGCTGGC |
| ***Firmicutes*** | GGAGYATGTGGTTTAATTCGAAGCA | AGCTGACGACAACCATGCAC |
| ***Bacteroidetes*** | GGARCATGTGGTTTAATTCGATGAT | AGCTGACGACAACCATGCAG |
| ***Proteobacteria*** | CATGACGTTACCCGCAGAAGAAG | CTCTACGAGACTCAAGCTTGC |
| ***Enterobacteriacea*** | GTGCCAGCAGCCGCGGTAA | GCCTCAAGGGCACAACCTCCAAG |
| ***Bifidobacterium*** | CGGGTGAGTAATGCGTGACC | TGATAGGACGCGACCCCA |
| ***Akkermansia muciniphila*** | CAGCACGTGAAGGTGGGGAC | CCTTGCGGTTGGCTTCAGAT |
| **β-actin** | GAGAAGATCTGGCACCACACC | GCATACAGGGACAGCACAGC |

AKP, Alkaline phosphatase; KLF4, gut-enriched Krüppel-like factor; Cdx1 (caudal-type homeobox-1) transcription factor; ER, estrogen receptor.

**Supplementary references**

1. Kaliannan K, Wang B, Li XY, Kim KJ, Kang JX: **A host-microbiome interaction mediates the opposing effects of omega-6 and omega-3 fatty acids on metabolic endotoxemia**. *Sci Rep* 2015, **5**:11276.

2. Kaliannan K, Hamarneh SR, Economopoulos KP, Alam SN, Moaven O, Patel P, Malo NS, Ray M, Abtahi SM, Muhammad N *et al*: **Intestinal alkaline phosphatase prevents metabolic syndrome in mice**. *Proceedings of the National Academy of Sciences of the United States of America* 2013, **110**(17):7003-7008.

3. Romanatto T, Fiamoncini J, Wang B, Curi R, Kang JX: **Elevated tissue omega-3 fatty acid status prevents age-related glucose intolerance in fat-1 transgenic mice**. *Biochim Biophys Acta* 2014, **1842**(2):186-191.

4. Lv PP, Tian S, Feng C, Li JY, Yu DQ, Jin L, Shen Y, Yu TT, Meng Y, Ding GL *et al*: **Maternal High Estradiol Exposure is Associated with Elevated Thyroxine and Pax8 in Mouse Offspring**. *Sci Rep* 2016, **6**:36805.

5. Lalles JP, Orozco-Solis R, Bolanos-Jimenez F, de Coppet P, Le Drean G, Segain JP: **Perinatal undernutrition alters intestinal alkaline phosphatase and its main transcription factors KLF4 and Cdx1 in adult offspring fed a high-fat diet**. *J Nutr Biochem* 2012, **23**(11):1490-1497.

6. Louie TJ, Cannon K, Byrne B, Emery J, Ward L, Eyben M, Krulicki W: **Fidaxomicin preserves the intestinal microbiome during and after treatment of Clostridium difficile infection (CDI) and reduces both toxin reexpression and recurrence of CDI**. *Clin Infect Dis* 2012, **55 Suppl 2**:S132-142.
